# Chloroplastic metabolic engineering coupled with isoprenoid pool enhancement for committed taxanes biosynthesis in *Nicotiana benthamiana*

**DOI:** 10.1038/s41467-019-12879-y

**Published:** 2019-10-24

**Authors:** Jianhua Li, Ishmael Mutanda, Kaibo Wang, Lei Yang, Jiawei Wang, Yong Wang

**Affiliations:** 1 Key Laboratory of Synthetic Biology, CAS Center for Excellence in Molecular Plant Sciences, Institute of Plant Physiology and Ecology, Shanghai Institutes for Biological Sciences, Chinese Academy of Sciences, Shanghai, 200032 China; 2 Henan Key Laboratory of Plant Stress Biology, Henan University, Kaifeng, 475004 China; 3 Shanghai Key Laboratory of Plant Functional Genomics and Resources, Plant Science Research Center, Shanghai Chenshan Botanical Garden, Shanghai, 201602 China; 4 National Key Laboratory of Plant Molecular Genetics, CAS Center for Excellence in Molecular Plant Sciences, Institute of Plant Physiology and Ecology, Chinese Academy of Sciences, Shanghai, 200032 China

**Keywords:** Metabolic engineering, Molecular engineering in plants, Secondary metabolism

## Abstract

Production of the anticancer drug Taxol and its precursors in heterologous hosts is more sustainable than extraction from tissues of yew trees or chemical synthesis. Although attempts to engineer the Taxol pathway in microbes have made significant progress, challenges such as functional expression of plant P450 enzymes remain to be addressed. Here, we introduce taxadiene synthase, taxadiene-5α-hydroxylase, and cytochrome P450 reductase in a high biomass plant *Nicotiana benthamiana*. Using a chloroplastic compartmentalized metabolic engineering strategy, combined with enhancement of isoprenoid precursors, we show that the engineered plants can produce taxadiene and taxadiene-5α-ol, the committed taxol intermediates, at 56.6 μg g^−1^ FW and 1.3 μg g^−1^ FW, respectively. In addition to the tools and strategies reported here, this study highlights the potential of *Nicotiana spp*. as an alternative platform for Taxol production.

## Abstract (web summary)

Engineering Taxol pathway in microbes needs to overcome the difficulty of expressing plant P450 enzymes. Here, the authors use a compartmentalized metabolic engineering strategy to construct the taxanes production pathway in chloroplasts of *Nicotiana benthamiana* and realize the production of taxadience-5α-ol.

## Introduction

The diterpenoid alkaloid Taxol (paclitaxel), originally isolated from the bark of the Pacific yew tree, is a highly effective anti-cancer agent widely used in the treatment of a multitude of cancers^1–4^. Despite its huge commercial success as a blockbuster anticancer drug and its promising efficacy against other non-cancer diseases, Taxol supply remains limited due to the poor extraction yields caused by extremely low accumulation levels in yew plants^5,6^. The past two decades saw considerable efforts being channeled toward development of alternative production methods to improve yields and build reliable, cost-effective production systems. Chemical synthesis and synthesis from cell and tissue cultures from *Taxus* species have been attempted^7–10^. However, productivity of these approaches cannot meet the increasing demand or the production pipelines are either too complicated or economically unfeasible. An alternative approach is production by semi-synthesis from two key intermediates of Taxol: baccatin III and 10-deacetylbaccatin III, both still extracted from renewable needles of *Taxus* trees or cell cultures^11,12^.

**Fig. 1:**
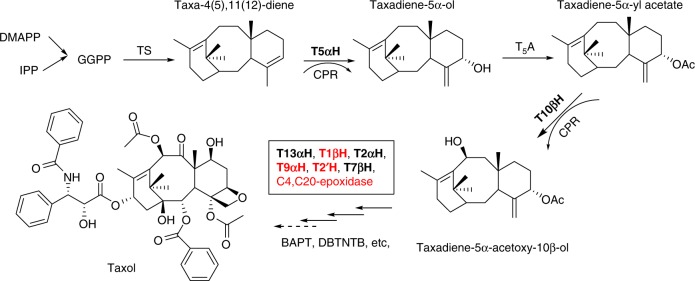
Scheme of Taxol biosynthesis. DMAPP, dimethylallyl diphosphate; IPP, isopentenyl diphosphate; GGPP, geranylgeranyl diphosphate; TS, taxadiene synthase; T5αH, taxadiene-5α-hydroxylase; CPR, cytochrome P450 reductase; T5A, taxadien-5α-ol-O-acetyltransferase; T10βH, taxane-10β-hydroxylase; T13αH, taxane-13α-hydroxylase; T1βH, taxane-1β-hydroxylase; T2αH, taxane-2α-hydroxylase; T9αH, taxane-9α-hydroxylase; T2′H, taxane-2′-hydroxylase; T7βH, taxane-7β-hydroxylase; BAPT, Baccatin III-3-amino, 13-phenyl-proanoyl-CoA transferase; DBTNBT, 3′-N-debenzoyltaxol-N-benzoyltransferase. P450 genes are marked in bold and unknown P450 genes are marked in red

It is against this backdrop that great efforts have been directed toward synthetic production of committed Taxol intermediates through optimization of microbial hosts^13–17^. Resources to complement these efforts have come from studies on Taxol pathway elucidation^18–21^, gene cloning^22,23^ and characterization of enzyme mechanisms^24–26^. Despite these significant gains, the failure to achieve total biotechnological production of Taxol lies in the non-effective expression of known pathway enzymes and the dearth of knowledge on its complex biosynthetic pathway. The Taxol biosynthetic pathway consists of at least 19 steps from GGPP (geranylgeranyl pyrophosphate)^18,27^, including a number of cytochrome P450 (CYP) mediated modifications^28,29^ (Fig. 1). Taxadiene, the first committed intermediate of the pathway^25,26^ has been produced in heterologous hosts with some degree of success, however, attempts to produce the next intermediate, taxadiene-5α-ol (a product of a cytochrome P450 enzyme, taxadiene-5α-hydroxylase, T5αH) have been met with disappointing results. Breakthrough work in *Escherichia coli* that employed a multivariate-modular approach towards metabolic pathway engineering achieved taxadiene yield of 1.0 g l^−1^, but this optimality was lost and titers fell considerably when T5αH was introduced into the same strain (only 50 mg l^−1^ of taxadiene-5α-ol)^17^. A possible hypothesis to explain this was that *E. coli* was not a tractable host for P450 chemistry, leading to the subsequent design of a microbial consortia of *E. coli* and *Saccharomyces cerevisiae*^14^. Unfortunately, the microbial competition in that hybrid system could not be completely avoided. Recently, through a rather extensive optimization of CYP expression, reductase-partner interactions and N-terminal modifications, this obstacle seemed to have been partially overcome in *E. coli*^16^, achieving almost 570 mg l^−1^ of oxygenated taxanes, but the extensive construction of vectors in this approach was noted to be too laborious, and fine-tuned taxoid production would still be perturbed by introduction of an additional CYP. Considering that there are nine P450 enzymes involved in the Taxol biosynthesis pathway, including four that remain to be uncovered (Fig. 1), huge challenges remain to be addressed to successfully engineer microbes as chassis for this pathway.

Compared with aforementioned microbial systems, plant systems present a more preferable host for terpenoid production^30–35^. Plants have inherent photosynthetic and carbon utilization machinery that confers plant-based systems many advantages as hosts for production of complex natural products. They can use atmospheric CO_2_ as carbon source rather than consuming exogenous sugars^36^, and can harness photosynthetic reducing power to drive P450 chemistry. Not only that, the effective expression of CYPs that poses a huge hurdle in microbial hosts seems much improved in plant platforms^37^. Moreover, given the presently incomplete Taxol pathway, an effective plant system is not only desirable for taxane engineering, but might present a better screening platform for the urgently needed discovery of remaining P450 genes to complete the Taxol pathway (Fig. 1). So far, there are few reports of taxadiene engineering in divergent plant cells via stable transformation of taxadiene synthase (TS). However, productivities of taxadiene in these systems are low^38–42^. Furthermore, synthesis of taxadiene-5α-ol has not been achieved in plant systems to date. Unlike in aforementioned microbial systems where at least three oxygenated taxoids including 5(13)-oxa-3(11)-cyclotaxane (iso-OCT), 5(12)-oxa-3(11)-cyclotaxane (OCT), and taxadiene-5α-ol have been produced in *E. coli*, co-expression of T5αH with TS in tobacco trichomes produced an unexpected OCT rather than taxadience-5α-ol^43^.

*Nicotiana benthamiana* is a rapid growing, high biomass, non-food crop that could be a viable alternative to microbial-based production systems. The use of *Nicotiana spp*. as a vehicle for engineering Taxol intermediates has been attempted before^42,43^. Those earlier efforts, however, achieved low taxadiene accumulation and failed to detect taxadiene-5α-ol. Here, we engineer two Taxol intermediates in *N. benthamiana* by chloroplastic metabolic engineering coupled with isoprenoid pool enhancement. By compartmentalizing TS, T5αH, and cytochrome P450 reductase (CPR) in the chloroplast, combined with increasing isoprenoid precursor pool size, we achieve high-level production of taxadiene and taxadiene-5α-ol in *N. benthamiana* leaves.

## Results

### Expression of yew-originated enzymes in *N. benthamiana*

To evaluate the suitability of *N. benthamiana* as a potential platform for the production of taxoids, *Agrobacterium*-mediated transient expression of selected Taxol biosynthetic pathway genes was performed. Full-length encoding sequences of TS, T5αH, and CPR were amplified from the needles of *Taxus chinensis* and fused with FLAG, HA and c-Myc tags at their C-terminals, respectively (primers are listed in Supplementary Table 1). Each sequence was inserted into the plant expression vector pEAQ-HT, which employs virus mechanisms to enhance heterologous protein expression and accumulation in plants^44^. *Agrobacterium* strain GV3101 harboring a single aforementioned construct was infiltrated into the leaves of 4-week old *N. benthamiana* to assess recombinant protein expression of each individual protein.

**Fig. 2:**
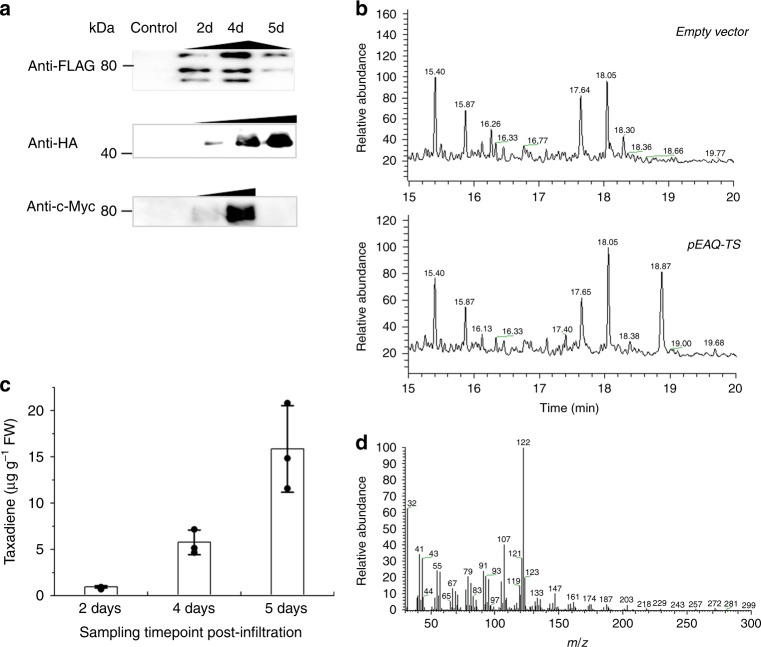
Expression of Taxus-originated proteins and committed taxadiene production in *N. benthamiana*. **a** Accumulation of TS, T5aH and CPR in plants by immunoblot analysis after agroinfiltration. Leaves infiltrated with *A. tumefaciens* cultures harboring the empty vector were set as controls. **b** GC–MS analysis of hexane extracts from infiltrated *N. benthamiana* leaves at 5 dpi (days post-infiltration); upper panel is control and bottom panel is TS-expression leaves. Accumulation of taxadiene (the peak at the retention time 18.8 min) was detected both in TS-expression leaves and TS, T5αH and CPR co-expressing leaves. No taxadiene-5α-ol was detected in the TS, T5αH, and CPR co-expression leaves. **c** Accumulation of taxadiene in agro-infiltrated leaves at 2, 4, and 5 dpi. **d** Mass spectra profile of the compound at 18.8 min matched exactly with the taxadiene mass spectra. Data in **c** represent the mean of *n* = 3 biologically independent samples (closed circles) and error bars show standard deviation. The source data of **a**, **c** are provided as a Source Data file

Leaf discs from the infiltrated parts were sampled at 2–5 days-post-infiltration (dpi) and analyzed for protein accumulation by immunoblotting using specific monoclonal antibodies. All three recombinant proteins could be readily detected in the assayed time period, but with different expression trends (Fig. 2a). TS was detectable at 2 dpi and accumulated to its highest levels at 4 dpi followed by a slow decrease afterwards. CPR showed a similar accumulation profile to that of TS. On the other hand, T5αH was detectable at 2 dpi and continuously increased through 5 dpi (Fig. 2a). These results demonstrate that the three proteins involved in Taxol biosynthesis could be successfully expressed in *N. benthamiana*.

### Heterologous production of taxadiene and its derivatives

Following the efficient expression of individual proteins, we next focused on construction of the Taxol synthetic pathway in *N. benthamiana*. GV3101-pEAQ-TS was firstly infiltrated into *N. benthamiana* leaves to synthesize taxadiene. At 5 dpi, leaf samples were extracted by hexane and analyzed by GC–MS. As expected, the presence of a new peak was observed at the retention time of 18.8 min, which was identified by selective ion monitoring at *m/z* 272 and 122 as taxa-4(5),11(12)-diene (Fig. 2b), and the mass spectrum (Fig. 2d) matched exactly with published literature^45^. A time-course analysis demonstrated that taxa-4(5),11(12)-diene initially accumulated at 2 dpi (about 0.8 μg g^−1^ fresh weight (FW) of tissues), significantly increased to almost 5.6 ± 1.3 μg g^−1^ at 4 dpi, and to 15.7 ± 4.7 μg g^−1^ at 5 dpi (Fig. 2c, Supplementary Fig. 1). These results demonstrate de novo production of taxadiene in *N. benthamiana* by utilization of the endogenous isoprenoid precursors. In addition, the compound at 18.8 min was isolated from the TS transient tobacco (Supplementary Methods, Supplementary Fig. 2) and the structure was confirmed to be taxa-4(5),11(12)-diene by ^1^H-NMR and ^13^C-NMR spectroscopy (Supplementary Fig. 13-16).

To extend our method to the biosynthesis of the desired taxadiene-5α-ol, *Agrobacterium* carrying TS, T5αH and CPR were mixed at 1:1:1 ratio for co-expression and infiltrated into *N. benthamiana* leaves. Leaves at 5 dpi were collected and hexane extracts were analyzed by GC–MS. However, no obvious taxadiene-5α-ol was detected, but a trace amount of the cyclic ether OCT was detected, consistent with previous co-expression of both TS and T5αH in *N. Sylvestris* trichome cells^43^.

### Organelle localization of heterologous proteins

Higher plants are complex multicellular organisms that utilize complex innate machinery for metabolism in compartmentalized intracellular organelles that have distinct cell/tissue specificities. We rudimentarily reasoned that the failure to produce taxadiene-5α-ol in tobacco was due to intracellular organelle separation of upstream and downstream enzymes. We speculated that limitations in taxadiene transport machinery from plastid to cytoplasm could be the main cause for non-production of taxadiene-5α-ol in heterologous plant cells.

**Fig. 3:**
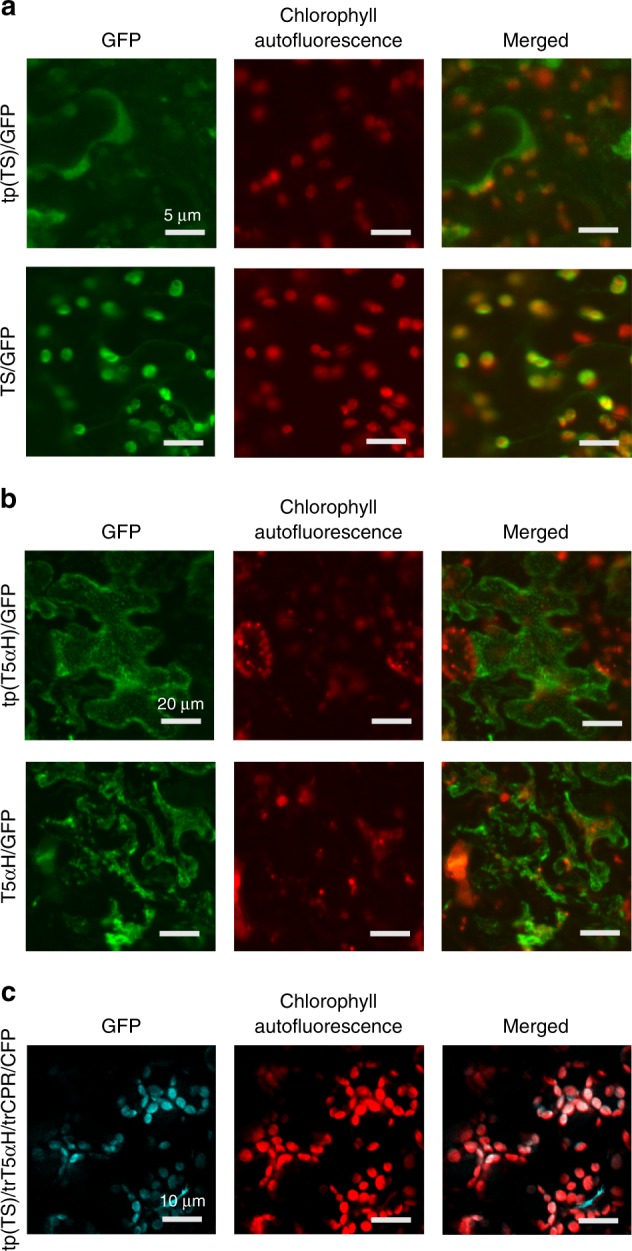
Intracellular organelle localization and targeting of heterologous proteins. Fluorescence labeling of the fusion proteins; **a** tp(TS)/GFP, TS/GFP; **b** tp(T5αH)/GFP, T5αH/GFP, and **c** tp(TS)/trT5αH/trCPR/CFP. *Agrobacterium* containing individual plasmids were introduced into the leaf lower epidermis of *N. benthamiana*. Leaf discs were harvested 3 days post infiltration and epidermal strips were observed by a confocal microscope. Scale bar indicates 5 μm in **a**, 20 μm in **b**, and 10 μm in **c**. *GFP* green fluorescent protein, *CFP* Cyan Fluorescent Protein

To confirm intracellular localization of the yew-originated proteins, amino acid sequences of TS, T5αH, and CPR were analyzed by TargetP, ChloroP, TMHMM, and Plant-Ploc (Supplementary Table 2). Results showed that TS possesses a putative chloroplastic targeting sequence with 58 amino acid residues at the N-terminus, while T5αH and CPR were predicted as membrane proteins that probably locate in the endoplasmic reticulum (ER), with 42 and 74 amino acid residues transmembrane region at their N-terminals, respectively (Supplementary Table 2). The full-length TS and the predicted peptide tp(TS) were fused with GFP (Green Fluorescent Protein), respectively, and transiently expressed in tobacco leaves. Indeed, the green fluorescence chimera derived from TS/GFP and tp(TS)/GFP completely matched the spontaneous red fluorescence derived from plant chloroplasts, confirming that the diterpene synthase TS localizes in chloroplasts, and that the N-terminal 58 amino acid residues are the determinant sorting peptide (Fig. 3a). In a similar way, the full length T5αH and its predicted transmembrane signal peptide were also fused with GFP and expressed in tobacco leaves. Indeed, fluorescence from both T5αH/GFP and tp(T5αH)/GFP was clearly separated from the red fluorescence of plant chloroplasts (Fig. 3b). These results confirm that TS and T5αH are localized in separate organelles in plant cells, which might present a barrier that separates taxadiene from its tailoring enzymes.

### Chloroplastic engineering for oxygenated taxanes production

**Fig. 4:**
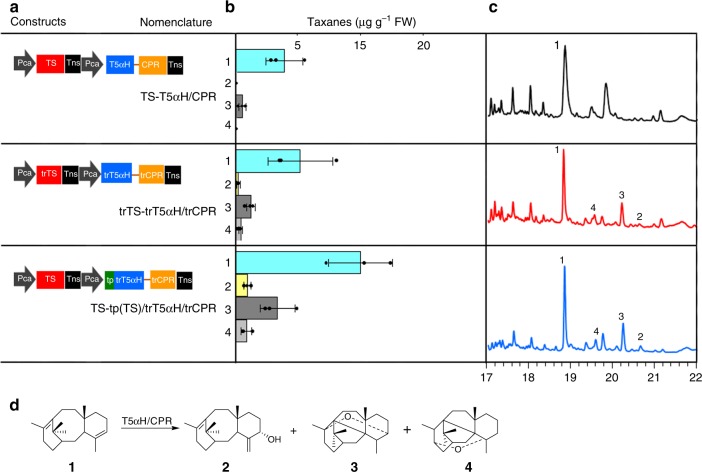
Production of mono-oxygenated taxanes by compartmentalized metabolic engineering. **a** Gene constructs used to engineer oxygenated taxanes in cytoplasm and in the chloroplast. Taxadiene 5α-hydroxylase and its electron donating cytochrome P450 reductase were fused to form T5αH/CPR, and the truncated protein version (trT5αH/trCPR) were also generated. For cytosolic engineering, truncated TS was co-expressed with trT5αH/trCPR under the same strong, constitutive promoter Pca; For chloroplastic engineering, the plastid targeting signal sequence, identified from TS, was fused to the 5′ end of trT5αH/trCPR and co-expressed with native TS in a single construct TS-tp(TS)/trT5αH/trCPR. Pca, 35 S cauliflower mosaic viral promoter; Tns, NOS terminator; n.d., no detected. **b** Enzymatic catalysis of T5αH on taxa-4(5),11(12)-diene (**1**) for generation of three mono-oxygenated taxanes; 5(12)-oxa-3(11)-cyclotaxane (OCT, **3**), 5(13)-oxa-3(11)-cyclotaxane (iso-OCT, **4**) and taxadiene-5α-ol (**2**) in the presence of CPR. **c** GC–MS analysis of extracts of selected transient leaves: upper panel - TS-T5αH/CPR; middle panel - trTS-trT5αH/trCPR; lower panel - TS-tp(TS)trT5αH/trCPR. **d** The enzymatic catalysis scheme of T5αH/CPR on taxa-4(5),11(12)-diene for biosynthesis of mono-oxygenated taxanes. Data in **b** represent the mean of *n* = 3 biologically independent samples (closed circles) and error bars show standard deviation. The source data of **b** are provided as a Source Data file

In order to explore the hypothesis that there is possible separation of T5αH from its substrate, two engineering strategies were undertaken: (1) redirection of TS into the cytosol, and (2) exportation of the T5αH-CPR fusion protein into the plastid (constructs are shown in Fig. 4a). For the first strategy, the signal peptides of TS and T5αH-CPR were completely removed, and the proteins were co-expressed in tobacco leaves. Compared to their native forms, such truncations did not significantly increase taxadiene levels, but slightly enabled accumulation of taxadiene-5α-ol at a low level of 0.21 μg g^−1^ FW (Fig. 4b, first and second panels), demonstrating that precise localization of enzymes and availability of substrate metabolite might facilitate generation of the desired oxygenated product.

Next, to redirect the reaction into the plastid, the chimeric truncated P450-CPR was fused with the 58-amino acid signal peptide of TS (resulting in tp(TS)/trT5αH/trCPR), and was co-expressed with full-length TS. In order to ascertain the cellular location of this chimera, CFP (Cyan Fluorescent Protein) was introduced in the carboxy terminus of the chimera, resulting in tp(TS)/trT5αH/trCPR/CFP. Upon expression in the leaves, the cyan fluorescence chimera derived from tp(TS)/trT5αH/trCPR/CFP completely matched the spontaneous red fluorescence derived from plant chloroplasts, confirming the success of compartmentalization of TS and T5αH/CPR in chloroplasts (Fig. 3c). After co-expression with TS, there was an increase in the amount of taxadiene to 9.9 μg g^−1^ FW (about 2.0-fold higher than engineering in the cytoplasm), and a remarkable increase in taxadiene-5α-ol (Supplementary Fig. 3) to 0.90 μg g^−1^ FW (almost 4.3-fold increase as compared to yields from cytoplasmic engineering) in tobacco leaves (Fig. 4b, third panel and Fig. 4c). These results demonstrate that proper, targeted localization of enzymes is critical to heterologous reconstruction of Taxol biosynthetic pathway, and co-localization in the plastid can lead to higher yields of the oxygenated product, possibly due to the richer isoprenoid precursor pool supplied by the MEP pathway. However, consistent with previous reports on T5αH engineering in *E.coli*^15,16^, we also identified OCT (Supplementary Fig. 5) and iso-OCT (Supplementary Fig. 7) that accumulated with taxadiene-5α-ol (Supplementary Fig. 3). We purified the oxidized taxane products from engineered tobacco leaves for NMR analysis (Supplementary Methods, Supplementary Note 1). Although giving some impurity signals probably due to the trace of mono-oxygenated taxoids in engineered tobacco leaves, we achieved a high level of purity of the products (Supplementary Fig. 4, 6, and 8) and the ^1^H-NMR data spectrum still matched well to the expected compounds (Supplementary Fig. 17-22). Though chloroplastic engineering boosted the overall yields of mono-oxygenated taxanes compared to cytosolic constructions, the ratio of taxadiene-5α-ol to OCT and iso-OCT were relatively unchanged at 1:5:1.5, with OCT as the major peak.

### Exploration of the isoprenoid precursor pathways of taxanes

**Fig. 5:**
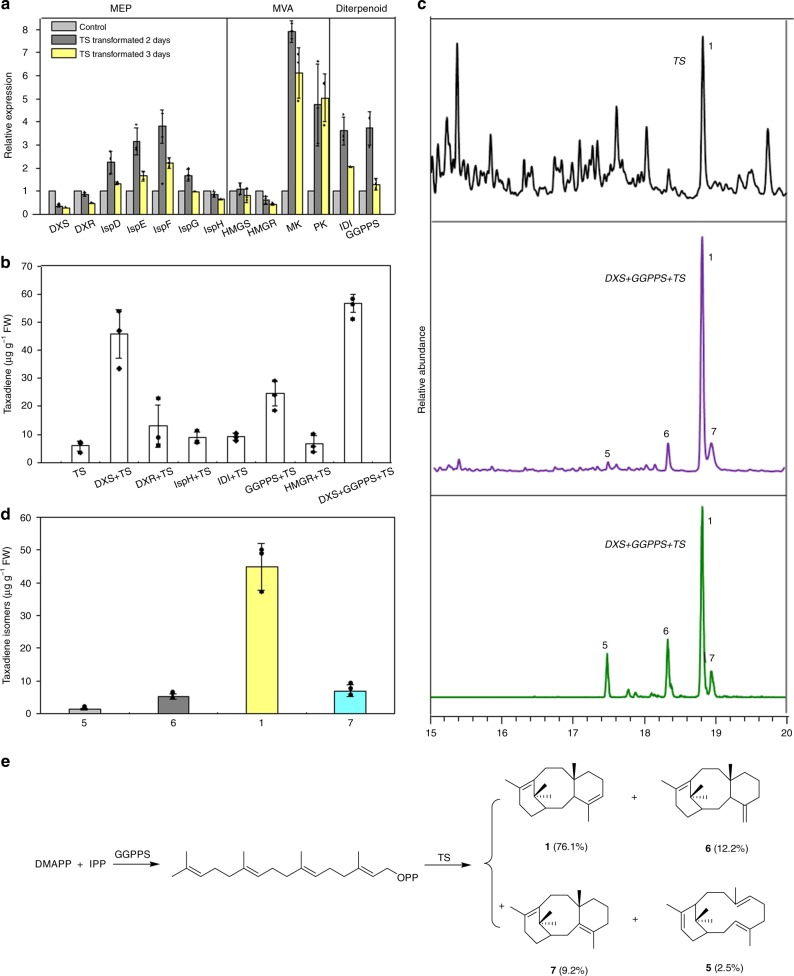
Identification of rate-limiting steps in isoprenoid pathway and precursor enhancement for taxadiene biosynthesis. **a** qRT-PCR analysis of MEP and MVA pathway enzymes in control and 2- or 3- days post infiltration transient leaves. **b** Taxadiene accumulation levels of plants engineered for isoprenoid precursor enhancement. **c** GC-MS analysis of leaf extracts of selected transient leaves. Upper panel - total ion chromatography (TIC) of TS; middle panel - TIC of DXS-GGPPS-TS; lower panel - selected ion monitoring (SIM) of specific m/z 272 for taxadiene from DXS-GGPPS-TS. The compounds taxa-4(5),11(12)-diene (**1**), verticillene (**5**), taxa-4(20),11(12)-diene (**6**), and taxa-3(4),11(12)-diene (**7**) represent various taxadiene isomers. **d** Accumulation of 4 taxadiene isomers in DXS-GGPPS-TS co-expression leaves; **e** The enzymatic catalysis scheme of taxadiene synthase on GGPP for generation of taxadiene isomers. Data in **a**, **b** and **d** represent the mean of n = 3 biologically independent samples (closed circles) and error bars show standard deviation. The source data of Figs. 5a, 5b, and 5d are provided as a Source Data file

*In planta*, the common precursors for terpenoid biosynthesis, isopentenyl diphosphate (IPP) and dimethyallyl diphosphate (DMAPP), are produced by the cytosolic MVA pathway and the plastidic MEP pathway^46,47^. To identify the limiting steps of these two routes with respect to the synthesis of taxoids, comparative transcriptional analysis of *N. benthamiana* MEP and MVA pathway genes in TS and empty vector transient leaves was performed. The transcript abundances of *IspD*, *IspE* and *IspF* of the MEP pathway, as well as *MK* and *PK* of the MVA pathway, were increased significantly in TS expressing leaves (Fig. 5a). Expressions of *IDI* and *GGPPS*, which encode proteins that regulate inter-conversion of DMAPP and IPP and condensation of IPP/DMAPP units to produce GGPP, respectively, were also upregulated after infiltration. However, transcriptional levels of *DXS*, *DXR* and *HMGR*, which encode enzymes that have been previously implicated to catalyze rate-limiting steps in the MEP and MVA pathways^48,49^, gradually decreased over the time taxa-4(5),11(12)-diene was accumulating (Fig. 5a), pointing to these tightly regulated genes as being bottlenecks in precursor supply. Such down regulation of genes encoding initial steps of pathways leads to decreased entry of metabolites, ultimately resulting in reduced precursor pools despite that intermediate steps were strengthened. We therefore focused on overexpressing these rate-limiting enzymes, together with *IDI*, *IspH*, and *GGPPS* to enhance IPP/DMAPP precursor pools.

To assess the extent to which these genes can perturb biosynthesis of taxanes, each sequence was separately assembled with *TS* to result in several two-cassette constructions that were subsequently transformed into the *Agrobacterium* strain. After expression in *N. benthamiana* (Fig. 5b), co-expression of *DXR*, *IspH* or *IDI* with *TS* led to only slight improvements in taxadiene production compared to TS alone, while DXS increased taxadiene production by more than 8-folds, up to 45.9 μg g^−1^ FW (Supplementary Fig. 24). Co-expression of GGPPS also increased taxadiene production by 4-folds (24.5 μg g^−1^ FW). This yield elevation demonstrates that overexpression of DXS could promote and help channel the flux of isoprenoid precursors towards downstream pathways, and overexpression of GGPPS could adjust the balance of precursors in favor of diterpenoid metabolism. The co-expression of HMGR with TS did not result in the expected improvement of taxadiene yield in tobacco, but significantly improved a compound that had a very similar retention time to taxadiene (nearly eluting as a single peak with taxadiene at low concentrations) at 18.75 min (Supplementary Fig. 25) with a mass spectrum showing m/z of 272, 257, 122 and 107, similar to taxadiene (Supplementary Fig. 24, 25) but with additional m/z of 218, 203, 175, and 147. To confirm whether the contaminant was not taxadiene or its derivative, we once again overexpressed HMGR and its truncated version (tHMGR) in stable TS transformed tobacco lines (Supplementary Fig. 28, 29). Extracts from these lines showed that HMGR could produce the contaminant (tR = 18.67 min), which is not obviously detectable in TS transgenic leaves. The yield of this compound improved by 10-folds in tHMGR extracts compared to HMGR overexpression (Supplementary Fig. 29C). At such high concentrations of the contaminating metabolite, taxa-4(5),11(12)-diene could be slightly separated from the contaminant, and detailed analysis revealed that only this peak was dramatically enhanced by overexpressing tHMGR, while the yield of taxadiene was not enhanced by overexpressing either HMGR or tHMGR. To examine if this compound was related to the introduced taxadiene pathway, we also overexpressed HMGR/tHMGR in wild-type tobacco and we still could detect the peak (Supplementary Fig. 30), clearly demonstrating that the compound originated from the native plant. We attempted to identify this compound by NMR analysis, but unfortunately failed to purify enough high quality amounts for structure analysis. However, based on the mass spectra, we speculated it to be a terpenoid derivative natively produced by *N. benthamiana* possibly in response to agrobacterium infection.

### Taxanes are predominantly derived from the MEP pathway

It has been widely known that diterpenoids are synthesized via the plastidic pathway in plants, but there are contrasting reports on the precursor supply pathway involved in the biosynthesis of taxanes. Glucose labeling experiments revealed that the taxane carbon skeleton is of MEP origin in *T. chinensis* cultured cells^50^. However, another independent study concluded that both the non-mevalonate and mevalonate pathways are involved in the biosynthesis of taxanes in cultured cells of *T. baccata*^51^. In the current study, over expression of HMGR/tHMGR, the bottleneck step of MVA pathway, failed to improve the production of taxadiene whereas co-expression of DXS significantly improved the yield of taxadiene by 8-folds. These results support that the taxane skeleton predominantly originates from the MEP pathway.

To gain a better understanding of the origin of the taxane carbon skeleton in higher plants, we separately blocked the MEP and MVA pathways by supplementing the MS media of stably-transformed transgenic line NTS1-T2 plants (Supplementary Fig. 26) with fosmidomycin (an inhibitor of the MEP branch of the terpenoid pathway) and lovastatin (an inhibitor of the mevalonate branch). The phenotypes of lovastatin-inhibited NTS1-T2 plants showed significant reduction in stem and adventitious root length, but the synthesis of chlorophylls and carotenoids was not significantly altered (Supplementary Fig. 27A-D). On the other hand, transgenic plants growing on fosmidomycin did not manifest similar growth deformities, but their leaves were chlorotic, apparently due to a generalized decrease in chlorophylls and carotenoids (Supplementary Fig. 27B–D). We also examined taxadiene production in control and inhibitor-treated plants after 28 days of growth, and the results showed that the NTS1-T2 control lines could produce taxadiene at levels of 15.7 ± 3.2 μg g^−1^ FW while the MVA-inhibited plants decreased taxadiene production by 40.1% to the level of 9.7 ± 2.4 μg g^−1^ FW (Supplementary Fig. 27E). Inhibition of the MEP pathway by fosmidomycin totally suppressed taxadiene biosynthesis in NTS1-T2 plants. These results suggest that in plants, the taxane scaffold predominantly originates from the MEP pathway and not the MVA pathway, and also supports that chlorophylls and carotenoids are primarily synthesized from MEP-derived precursors while the MVA pathway is the predominant source of sterols that play a crucial role in the regulation of root growth.

### Synergistic expression of upstream bottleneck enzymes

Next, we tested the synergistic effect of co-expression of DXS, and GGPPS on taxadiene production. Expression cassettes of each of these genes were assembled with TS in single constructs and transiently expressed in tobacco leaves. At 5 dpi, the taxadiene yield was up to 56.6 ± 3.2 μg g^−1^ FW, about 10-fold increase as compared to the single TS engineered plants (Fig. 5b). Interestingly, in addition to taxa-4(5),11(12)-diene, three other byproducts of TS were also detected (Fig. 5c–e), including verticillene (1.4 ± 0.2 μg g^−1^ FW, 2.5%, Supplementary Fig. 9), taxa-4(20),11(12)-diene (6.9 ± 1.8 μg g^−1^ FW, 12.2%, Supplementary Fig. 10), and taxa-3(4),11(12)-diene (5.2 ± 0.9 μg g^−1^ FW, 9.2%, Supplementary Fig. 11), consistent with a prior study^45^.

**Fig. 6:**
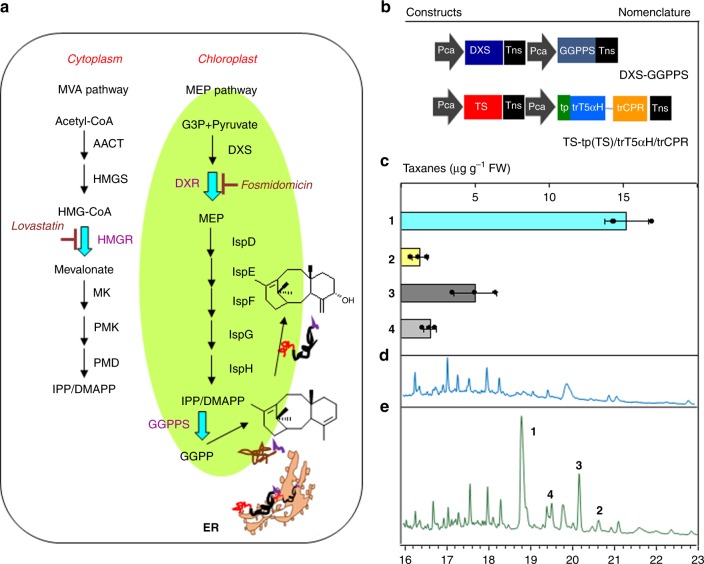
Chloroplastic metabolic engineering coupled with isoprenoid pool enhancement for committed Taxol intermediates in *N. benthamiana* cells. **a** Schematic representation of Taxol intermediate engineering in plant cells. Cytosolic engineering did not direct flux towards taxane biosynthesis, but enhanced a compound with similar retention time to taxadiene. The compound was speculated to be a native tobacco terpenoid. Chloroplastic engineering remarkably increased production of taxadiene and oxygenated taxanes, including taxadiene-5α-ol. MVA, mevalonate; AACT, acetoacetyl CoA thiolase; HMGS, hydroxymethylglutaryl coenzyme A synthase; HMGR, hydroxy 3 methylglutaryl coenzyme A reductase; MK, mevalonate kinase; PK, phosphomevalonate kinase; PMD, mevalonate pyrophosphate decarboxylase; MEP, 2-C-methyl-D-erythritol 4-phosphate; DXS, 1-deoxy-D-xylulose 5-phosphate synthase; DXR, 1-deoxy-D-xylulose-5-phosphate reductoisomerase; IDI, IPP isomerase; T5αH, taxadiene 5α hydroxylase; CPR, *Taxus cuspidata* cytochrome P450 reductase; IspD, 2-C-methyl-D-erythritol 4-phosphate cytidyltransferase; IspE, 4-diphosphocytidyl-2-C-methyl-D-erythritol kinase; IspF, 2-C-methyl-D-erythritol 2,4-cyclodiphosphate synthase; IspG, (E)-4-hydroxy-3-methylbut-2-enyl diphosphate synthase; IspH, isopentenyl/dimethylallyl diphosphate synthase. GGPPS, Geranylgeranyl diphosphate synthase. Lovastatin (10 μM) and Fosmidomycin (150 μM) were used to inhibit the MVA and the MEP pathways, respectively. **b** Gene constructs used to engineer oxygenated taxanes in the chloroplast. **c** The yield of taxanes produced in transient leaves at 5 dpi. **d** GC–MS analysis of tobacco leaf metabolites with GV3101 harboring empty vector; **e** GC–MS analysis of tobacco leaf metabolites with GV3101 harboring TS-tp(TS)/trT5αH-trCPR and DXS-GGPPS. Taxa-4(5),11(12)-diene (**1**) and three mono-oxygenated taxanes; taxadiene-5α-ol (**2**), OCT (**3**), and iso-OCT (**4**) are marked. Data in **c** represent the mean of *n* = 3 biologically independent samples (closed circles) and error bars show standard deviation. The source data of **c** are provided as a Source Data file

To further improve taxadiene-5α-ol yields, we then combined this precursor supply enhancement with chloroplastic engineering strategy, together with upregulation of GGPPS to channel isoprenoids towards diterpenoid biosynthesis (Fig. 6a). We mixed two *Agrobacterium* strains harboring TS-tp(TS)/trT5αH/trCPR and DXS-GGPPS (Fig. 6b) and infiltrated into *N. benthamiana*. There was an improvement of taxadiene-5α-ol accumulation to 1.3 μg g^−1^ FW when chloroplast engineering was combined with enhancement of precursor supply (Fig. 6c, e). Compared to chloroplastic engineering alone, we also noted an increase in OCT and iso-OCT that could have limited accumulation of taxadiene 5α-ol. The ratio of taxadiene-5α-ol:OCT:iso-OCT in chloroplast-engineered lines (1:4:1.6) was nearly similar to that in cytosolic engineered-plants, demonstrating that T5αH directed into the chloroplast could achieve a comparable product profile with T5αH in its natural environment on the ER membrane.

Following the successful engineering of taxadiene-5α-ol in tobacco leaves, we then attempted to engineer further downstream Taxol intermediates. We infiltrated tobacco leaves with the *Agrobacterium* harboring pEAQ-TS-tp(TS)/trT5αH/trCPR-tp(TS)/T5A-tp(TS)/trT10H (with added taxane-10β-hydroxylase; T10βH and taxadiene-5α-ol-*O*-acetyltransferase; T5A) and combined with DXS-GGPPS strain, expecting production of taxadiene-5α-acetoxy-10β-ol^19,52^. Unfortunately, this metabolite was not observed in the GC-MS analysis of leaf extracts. However, a metabolite was observed at the retention time of 23.5 min (Supplementary Fig. 12). Further analysis of this peak showed dominant masses of m/z 304, 287, 221, 189, and 147 (Supplementary Fig. 12D), which probably represents taxadiene-5α,10β-diol. We could not pursue total identification of the compound due to low yields in the leaf and the absence of an authentic standard.

## Discussion

Production of the anticancer drug Taxol and its intermediates in heterologous hosts is a promising alternative to current methods that employ extractions from twigs and cell cultures of the yew plants. Here, we engineered the partial Taxol pathway via compartmentalizing TS, T5αH and CPR in the chloroplasts of *Nicotiana benthamiana*. Combining this compartmentalization strategy with enhancement of precursors and diversion of carbon towards taxane biosynthesis resulted in accumulation of relatively high levels of taxadiene (56.6 ± 3.2 μg g^−1^ FW), and most importantly, successful accumulation of taxadiene-5α-ol (1.3 ± 0.5 μg g^−1^ FW) in tobacco leaves. This work in a high-biomass plant establishes a starting point for future optimizations of taxol production in heterologous plants. It also presents a plant platform for screening missing enzymes of the taxol pathway. Further, it provides tools and strategies that could be useful in guiding future efforts for production of other natural products in plant hosts.

*Nicotiana benthamiana* is a high biomass, non-food crop that is cheap and has a rapid growth rate and a well-established system. It became our platform of choice in this study because Taxol is a plant natural product and many plant terpene synthases and cytochrome P450s have been successfully expressed in this plant before^35,53,54^. What’s even more attractive, a number of technological improvements have been developed for *N. benthamiana*^55,56^, including improvements in scalability of agroinfiltration using vacuum infiltration recently demonstrated to be powerful in large-scale production of oxidized triterpenes^53,57^.

Low expression and poor functionality of CYPs involved in decoration of the taxadiene olefin ring is the major stumbling block hampering efforts to further construct the pathway past taxadiene-5α-ol in yeast and *E. coli*^13,15–17^. Compared to classical microbial and yeast chassis, plants seem more compatible with heterologous expression of plant enzymes like cytochrome P450s in terms of availability of suitable endomembranes, cofactor availability and protein folding machinery^37^. However, despite these merits, the complex plant cell presents several drawbacks for it to be used as a successful chassis for heterologous production of natural compounds. It is highly compartmentalized, and transport mechanisms of metabolites are not fully understood. It also has both the MEP and MVA pathways and a repertoire of native enzymes and pathways that are difficult to control, predict and optimize. Thus, despite being preferred hosts for plant P450s, past attempts to construct the Taxol pathway for production of taxadiene-5α-ol in plants were completely unsuccessful, while only low taxadiene accumulation was achieved^38,41–43^.

Recently, compartmentalized metabolic engineering in *Nicotiana spp*. has emerged as a promising strategy to overcome some of these problems and improve yields of terpenoids^31,34,58^. Recent engineering efforts to target P450s to the thylakoid membranes through the pH-dependent twin-arginine translocation (Tat) complex have demonstrated the competitiveness of the chloroplast as a site for light driven P450-mediated hydroxylation^59^. In this work, we confirmed that TS possesses a plastid targeting signal peptide and that T5αH and CPR possess ER-localization signal peptides (Fig. 3, Supplementary Table 2), implying there is a physical barrier between the site of production of taxadiene and the site of its subsequent oxygenations. We thus employed a compartmentalized metabolic engineering strategy to introduce T5αH and CPR into the chloroplast (Fig. 6). Given that TS is natively targeted to the chloroplast, we considered mimicking TS post-translational processing and localization in the chloroplast in our importation strategy of T5H-CPR fusion construct into the chloroplast compartment. Therefore, the use of the native TS signal peptide as a chloroplast targeting peptide was considered more optimal compared to the Tat complex in our engineering, to ensure similar translocation strategies and close proximity of TS and the P450-CPR tailoring enzymes.

Another longstanding challenge in bioengineering terpenes in heterologous hosts is the limited supply of IPP and DMAPP, the universal precursors of all terpenes. We compared the expression profiles of MVA and MEP genes in control (empty vector) and TS-transgenic plants and identified, in the context of engineering taxane pathway, that DXS, DXR (MEP pathway) and HMGR (MVA) were downregulated in TS-expressing plants (Fig. 5a). We speculated that this could have been caused by an inherent mechanism to limit carbon flow through the pathways in response to the introduced gene. To re-direct carbon flow towards diterpenoid synthesis, we upregulated GGPPS together with the upstream MEP and MVA genes. Some or all of these genes have been targeted before for production of taxadiene and other diterpenoids, achieving varying degrees of success^13,41,49^. Compared to overexpressing TS alone, co-expression with GGPPS and DXS resulted in a significant increase in taxadiene, and our chloroplastic engineering of oxygenated taxanes improved the levels of taxadiene-5α-ol from 0.9 μg g^−1^ FW to 1.3 μg g^−1^ FW when combined with precursor enhancement.

Co-expression of HMGR with TS increased accumulation of a metabolite with a very similar retention time to taxa-4(5),11(12)-diene. We sought to explore this further and possibly leverage on the MVA pathway for improving production of taxanes. Overexpression of a better performing tHMGR clearly improved the closely related contaminating peak, but not taxa-4(5),11(12)-diene. There are numerous conflicting reports regarding the role of each pathway in supplying carbon for Taxol synthesis: evidence exist that there is little or no crosstalk between the pathways^60^, but inhibitors of each pathway have been shown to inhibit Taxol biosynthesis^51,61^. To further address the issue of the source of precursors for Taxol biosynthesis in a heterologous plant, we generated stable TS-transgenic plants and grew them on media with either fosmidomycin or lovastatin, the inhibitors of each pathway. In agreement with the DXS and HMGR overexpression result, pathway inhibition data showed that only fosmidomycin completely blocked taxadiene production in tobacco leaves while lovastatin-inhibited plants accumulated substantial amounts of taxadiene. The observed differences in the phenotypes in treated plants demonstrated the effectiveness of the inhibition treatments of both the MVA and the MEP pathways, giving weight to the inference that the MEP pathway supplies the bulk of the isoprenoid precursors for the taxane carbon backbone in plants.

Together with taxa-4(5),11(12)-diene, we also identified verticillene, taxa-4(20),11(12)-diene and taxa-3(4), 11(12)-diene in hexane extracts of transgenic plants. Furthermore, though the engineered plants accumulated taxadiene-5α-ol, we also identified two other mono-oxygenated taxanes OCT, and iso-OCT. The appearance of these latter compounds instead of the desired taxadiene-5α-ol was observed in other studies^15,43,62^ and led to different proposals for the mechanism of catalysis by T5αH. Based on several lines of evidence, a proposal was forwarded that T5αH catalyzes taxadiene to an epoxide intermediate that decomposes to many products like taxadiene-5α-ol, OCT and iso-OCT^62^ rather than through the earlier proposed radical abstraction mechanism^21,24^.

Our attempt to construct the pathway further by adding T10βH and T5A did not yield expected results of taxadiene-5α-yl acetate and taxadiene-5α-acetoxy-10β-ol, but we observed a peak at 23.5 min (with dominant masses at m/z 304, probably representing taxadiene-5α,10β-diol) that accumulated to only low levels. There was a marked decrease in the taxadiene-5α-ol peak following introduction of T10βH and T5A (Supplementary Fig. 12), and given that there were no observed changes in the expression of TS, T5aH and CPR protein levels, this demonstrates consumption of taxadiene-5α-ol in transgenic leaves. It remains to be seen if this peak we identified is one of the committed mono-oxygenated taxane intermediates that deserves to be optimized for increased production, or if it is one of the many dead-end products from spontaneous degradation of the epoxide intermediate.

Metabolic flux in plants is tightly controlled by complex regulatory mechanisms, and an improved understanding of gene and protein expression landscape is fundamental for achieving precise control and fine-tuning of engineered pathways. Gene expression analysis of native MVA and MEP pathway genes in the early period following agroinfiltration demonstrated varying trends, implying internal adjustment and transcriptional regulation of native precursor pathways. We targeted genes that decreased mRNA expression levels for overexpression and achieved an eight folds improvement in taxadiene and oxidized terpenes, demonstrating the importance of an improved understanding of temporal regulation.

Altogether, this study presents *N. benthamiana* as an attractive and amenable alternative platform to microbial systems for engineering production of taxanes. Compared to well-established microbial systems, heterologous plant systems present challenges as biosynthetic machineries due to their complex cell structure and difficulties in optimizing growth conditions following genetic manipulation. Our approach using compartmentalized engineering coupled with precursor enhancement resulted in comparatively high taxa-4(5),11(12)-diene yields and most importantly, accumulation of taxadiene-5α-ol in a plant platform. Given the attractiveness of plant systems as safe, cost-effective platforms that harness sunlight and natural carbon to synthesize heterologous compounds, the taxane yields in fresh tobacco leaf tissue reported here are comparable to other reports in microbial and yeast platforms. Moreover, with the successful production of committed taxadiene-5α-ol in plant cells, it is highly expected that the convenient expression of P450s in plant systems as demonstrated here might help establish a fast screening platform to complete the Taxol pathway in the foreseeable future. In conclusion, this study presents a significant and crucial step for future biotechnological efforts towards Taxol production, and the strategies and tools employed here could be valuable in circumventing bottlenecks in plant systems and in guiding ongoing efforts to bioengineer Taxol production in plants.

## Methods

### Plant material and growth conditions

*Nicotiana benthamiana* plants were grown from seeds in soil in a growth chamber with a 16-h light and 8-h dark photo-cycle at 25 °C. Leaves of four-week-old *N. benthamiana* plants were used for *Agrobacterium*-mediated transient transformation using standard protocols.

### Construction of plant transformation vectors

Total RNA was extracted from the needles of cultivated yew (*Taxus chinensis*) using Omini Plant RNA Kit (cwbiotech, China) and reverse transcribed to cDNA using PrimeScript RT reagent kit with gDNA eraser (Takara, Japan). ORFs of *TS* (Taxadiene synthase, accession no. U48796), *T5*α*H* (Taxadiene 5α-hydroxylase, AY289209), *CPR* (*Taxus cuspidata* Cytochrome P450 reductase, AY571340), *HMGR* (*Taxus* *×* *media* 3-hydroxy-3-methyl glutaryl coenzyme A reductase, AY277740), *DXS* (*Taxus* *×* *media* 1-deoxy-D-xylulose-5-phosphate synthase, AY505129), *DXR* (*Taxus* *×* *media* 1-deoxy-D-xylulose-5-phosphate reductoisomerase, AY588482), *IDI* (*Taxus* *×* *media* isopentenyl diphosphate isomerase, KP970677), *GGPPS* (*Taxus canadensis* GGPP synthase, AF081514) were amplified with PrimerStar Max DNA polymerase (Takara, Japan). Each sequence was ligated into the digested vector pEAQ-HT (kindly provided by Dr. George Lemonossoff, John Innes Centre, UK) using One-step-directed cloning kit (Novoprotein, Shanghai). The primers used are listed in Supplementary Table 1.

For expression of multiple genes, cassettes were assembled using Golden Gate assembly method^63^ (Supplementary Fig. 9). A golden gate plasmid pEAQ-HT-GG was constructed by PCR amplification and Gibson assembly to remove the region containing promoter, 5′UTR, multi-cloning site, 3′UTR and terminator sequences, and to insert two Bsa-I restriction sites linked to two distinct recognition sites separated by a linker sequence (Supplementary Fig. 9). ORFs were amplified from pEAQ-HT vectors carrying the gene of interest starting from promoter and ending with terminator and flanked with Bsa-I restriction sites linked with distinct recognition sites. The primers used are listed in Supplementary Table 5. Purified amplicons were inserted into pEAQ-HT-GG by Golden Gate assembly to construct DXS-TS, DXR-TS, IspH-TS, HMGR-TS, DXS-HMGR-GGPPS, and DXS-GGPPS-TS. After transformation into *Agrobacterium*, positive clones were identified by Xho I digestion and confirmed by sequencing.

The predicted N-terminal plastid targeting sequence of TS and the transmembrane sequence of T5αH were fused with GFP, to generate tp(TS)/GFP and tp(T5αH)/GFP, respectively. The full length of TS and T5αH were fused with GFP to generate TS/GFP and T5αH/GFP chimeric gene with linker GGGGG. Primers used for fusion constructs are listed in Supplementary Table 6.

For compartmentalized engineering of Taxol biosynthetic pathway in *planta*, the chimera T5αH/CPR was firstly constructed with linker GSTGS. For cytosolic, engineering, truncated TS was fused with truncated T5αH/CPR to produce trTS-trT5αH/trCPR by Gibson assembly method. For chloroplastic engineering, the plastid targeting signal sequence of TS was fused to the 5′ end of trT5αH/trCPR and co-expressed with native TS in a single construct TS-tp(TS)/trT5αH/trCPR (Fig. 5a). Primers used are listed in Supplementary Table 6.

### Transient expression in *N. benthamiana*

All binary plasmids were introduced into *Agrobacterium* strain GV3101 for transient expression in tobacco. *Agrobacterium* strains were grown at 28 °C at 220 rpm for 16 h in yeast extract peptone (YEP) medium with kanamycin (50 mg l^−1^), rifampicillin (25 mg l^−1^) and gentamycin (25 mg l^−1^). Cells were harvested by centrifugation for 10 min at 5000 × g and then suspended in MMA buffer containing 10 mM MES (2-[N-morphollino]-ethanesulfonic acid, Sangon Biotech (Shanghai) Co., Ltd.), 10 mM MgCl_2_ and 100 μM acetosyringone to a final OD_600_ of 0.5, and incubated at room temperature with gentle shaking for 3-4 h. Strains were slowly infiltrated into the abaxial side of leaves of *N. benthamiana* plants using a 1 ml syringe to cover the whole leaf. The plants were then grown under greenhouse conditions until further analysis.

For co-infiltration assays, equal volumes of culture of the *Agrobacterium* strains harboring a respective plasmid were mixed before centrifugation; the pelleted cells were resuspended in MMA buffer to a final OD_600_ of 0.5.

### Western blot

The agro-infiltrated leaf tissues were homogenized in 500 μl of lysis buffer (50 mM Tris-HCl, 150 mM NaCl, 1 mM EDTA, 10% glycerol, 2 mM Na_3_VO_4_, 25 mM β-glycerophosphate, 10 mM NaF, 0.05–0.1% Tween, 1 mM PMSF, pH 7.5). The homogenates were centrifuged at 14,000 × *g* for 20 min at 4 °C, then the supernatants were collected and the protein concentration was determined by the Bradford method^64^. Aliquots of 50 μg of protein were separated on 8% acrylamide gels and were then electro-transferred to PVDF membranes using the Bio-Rad gel transfer apparatus in TBST buffer for 1 h. Membranes were blocked in a 3% BSA/TBST buffer solution at room temperature for 0.5 h, followed by incubation with the appropriate primary antibodies at 4 °C overnight [anti-FLAG, Abmart, cat # M20008 at 1:1000 dilution; anti-HA, Roche, cat # 12013819001 at 1:1000 dilution; anti-c-Myc, Millipore, cat # 05-724 at 1:1000 dilution). Following incubation, the membranes were washed three times with TBST and incubated with an HRP-conjugated goat, anti-mouse secondary antibody (Abmart, lot # M21001 at 1:1000 dilution). The blots were washed again three times with TBST buffer and the immunoreactive bands were detected using an enhanced chemiluminescence method.

### Bioinformatics analysis

Subcellular localization prediction of TS, T5αH and CPR was performed using the plant protein subcellular location prediction tool (Plant-PLoc)^65^. The presence of chloroplast transit peptides and their location was predicted using ChloroP service^66^. The transmembrane regions of T5αH and CPR were predicted using the TMHMM program^67^.

### Fluorescence visualization

At 3 dpi (days post infiltration), the epidermal strips of *N. benthamiana* leaves were mounted in water and added to a 22 × 22-mm cover slip for direct observation by fluorescence microscopy. Observations were made with a confocal microscope (OLYMPUS, FV10i) using an excitation wavelength of 405, 488, 556 nm, and the images were collected by Olympus Fluoview 4.0 software.

### RNA isolation and quantitative real-time PCR

Total RNA was isolated from tobacco leaves using the Ultrapure RNA Kit (Cwbiotech). Purified RNA was used for cDNA synthesis with PrimeScript RT Reagent Kit (Takara). Quantitative real-time PCR was performed using UltraSYBR Mixture (Cwbiotech) on the Realplex Master Cycler (Eppendorf). For querying target candidate genes, the genomic information of *N. benthamiana* was downloaded from https://www.ncbi.nlm.nih.gov/assembly/GCA_000723945.1/. MEP and MVA pathway genes that have already been characterized from *Nicotiana spp*. were used as query sequences for *N. benthamiana* genomic local blast for target candidates (Supplementary Table 3). Primers were designed (Supplementary Table 4) and used to quantify gene expression levels between the control and TS-transformed plants after 2 or 3 dpi. Actin was used for internal normalization in each RT-qPCR. Samples were collected from three biologically independent plants and analyses were conducted using technical replicates.

### GC–MS analyses of taxoids

For analysis of taxadiene and taxadien-5α-ol, 1.0 g of infiltrated leaf tissues were frozen in liquid nitrogen, ground to a fine powder, and extracted three times with 5 ml hexane and 20 min sonication cycles. Samples were then extracted once with hexane:ethyl acetate mixture (4:1 v/v) containing 200 ng of nonadecane as an internal standard. The organic solvent phases were combined and dried under a stream of nitrogen. For GC-MS analysis, the extracts were re-dissolved in 1 ml of hexane: ethyl acetate (4:1, v/v) and 1 μl of each sample was injected directly into a Trace GC Ultra gas chromatograph coupled to ISQ mass spectrometer (Thermo Scientific). Separation was achieved with an HP5ms column (30 m × 250 μm × 0.25 μm thickness) in splitless mode. Ultra helium was used as the carrier gas at a flow rate 1.0 ml/min. The oven temperature was kept constantly at 80 °C for 2 min, and then increased to 200 °C at the increment of 10 °C/min, and finally held for 10 min. The injector and transfer line temperatures were set at 200 °C and 250 °C, respectively. The column effluent was ionized by electron impact ionization at 70 eV. Mass spectra were acquired in the range of m/z 30-300. Taxadienes and mono-oxygenated taxoids were quantified by determination of their total ion count peak area in comparison to the peak area of the internal standard nonadecane. All analyses were conducted using three biologically independent samples. We infiltrated at least three plants in each independent experimental run and a minimum of two independent runs was conducted.

### Reporting Summary

Further information on research design is available in the Nature Research Reporting Summary linked to this article.

## Supplementary information

Supplementary Information

Peer Review

Reporting Summary

Source data

## Data Availability

Data supporting the findings of this work are available within the paper and its Supplementary Information files. A reporting summary for this Article is available as a Supplementary Information file. The datasets generated and analyzed during the current study are available from the corresponding author upon reasonable request. The source data underlying Figs. 2a, c, 4b, 5a, b, d, and 6c, as well as Supplementary Figs. 26j and 27b–e are provided as a Source Data file.
