# Supplementary material for: Chloroplastic metabolic engineering coupled with isoprenoid pool enhancement for committed taxanes biosynthesis in Nicotiana benthamiana
Source: Nat Commun. 2019 Oct 24;10:4850. doi: 10.1038/s41467-019-12879-y (PMC6813417; doi:10.1038/s41467-019-12879-y)
Supplement: Supplementary file 1 — Supplementary Information [file 41467_2019_12879_MOESM1_ESM.pdf]

Chloroplastic metabolic engineering coupled with isoprenoid pool enhancement for committed taxanes biosynthesis in *Nicotiana benthamiana*

Li *et al.*

Supplementary Methods

Purification of taxa-4(5),11(12)-diene (1)

19 *Nicotiana benthamiana* plants were used for *Agrobacterium*-mediated transient transformation with GV3101-pEAQ-DXS-GGPPS-TS. At 5 dpi, a total of 83 leaves were harvested and ground into a fine power in liquid nitrogen. The final leaf powder sample (64 g) was then extracted three times with 200 ml n-hexane. The organic solutions with the crude taxane-containing extracts were dried over anhydrous Na_2SO_4, and concentrated under reduced pressure to give a dark green oil which was then purified by normal phase chromatography (Silica gel 200-300 stationary phase). The fractions were developed with n-hexane by thin layer chromatography (TLC) and observed by spraying with 5% H_2SO_4-vanillic aldehyde. The taxane-containing fractions (fraction 2 - fraction 8) were pooled and concentrated in a vacuum to yield taxa-4(5),11(12)-diene as a colourless oil. The final yield was approximately 1.2 mg.

Purification of iso-OCT (4), OCT (3), and taxadiene-5α-ol (2)

Another 78 agrobacterium-infiltrated *Nicotiana benthamiana* plants (co-infiltrated with GV3101-pEAQ-DXS-GGPPS and GV3101-TS-tp(TS)/trT5H/trCPR) were harvested at 5 dpi. A total of 374 g of plant tissue was ground into a fine power and extracted with organic solution (n-hexane/ethyl acetate, 4/1). The residue was loaded onto a silica gel column (ø = 4 cm, height = 35 cm) and eluted successively with 200 ml of hexane, hexane/ethyl acetate (100:1), hexane/ethyl acetate (100:2), hexane/ethyl acetate (100:3), hexane/ethyl acetate (10:1), and finally hexane/ethyl acetate (4:1). 10 ml of effluent was collected in a glass tube and fractions were monitored by TLC with n-hexane/ethyl acetate (19:1) as the development solution. Four fractions (Fa-Fd) were finally obtained. Fa contained taxadiene and its isomers (tubes 3-10); Fb contained iso-OCT (tubes 41-42), Fc contained OCT (tubes 43-45), and Fd contained taxadiene-5α-ol (tubes 54-57).

Fb and Fc were respectively reloaded on a silica gel column (Silica gel 300-400 stationary phase, ø = 1 cm, height = 30 cm) and eluted successively with 50 ml of hexane/ethyl acetate (100/0.3), hexane/ethyl acetate (100/0.5), hexane/ethyl acetate (100/0.7), hexane/ ethyl acetate (100/1), hexane/ ethyl acetate (100/1.2). Fd was reloaded on a silica gel column and eluted with 50 ml of hexane/ethyl acetate (100/1.0), hexane/ethyl acetate (100/1.2), hexane/ethyl acetate (100/1.5), hexane/ethyl acetate (100/1.8), hexane/ethyl acetate (100/2.0). The focal sub-fractions were combined and further separated by pre-TLC with hexane/ethyl acetate (10:0.6).

Transformation of *N. benthamiana* by *Agrobacterium* LBA4404-pEAQ-TS infection

Transformation of *N. benthamiana* with the TS gene was accomplished by the leaf disc method [1,2], with slight modifications. Briefly, leaves from 1-month old *N. benthamiana* were aseptically cut to approximately 0.5 cm^2 discs. The leaf explants were then precultured in 1/2 MS media containing 3 % sucrose (w/v), 0.05 mg/L NAA (1-Naphthaleneacetic acid), 0.5 mg/L 6BA, and 0.3 % gelzan (w/v), after which they were incubated in the dark at 25°C for 24 h. The *A. tumefaciens* LBA4404 harboring pEAQ-TS was grown overnight at 28°C and

120 rpm in yeast extract peptone (YEP) medium containing 50 mg/L kanamycin, 50 mg/L rifampicin, and 25 mg/L gentamycin, after which it was centrifuged at 5,000 rpm for 10 min. The pellet was then dissolved in 10 mM MES and $MgCl_2$ until an absorbance of 0.5 at OD_{600} was obtained. Acetosyringone was added to the resuspended culture to a final concentration of 150 µM, followed by incubation for 4 h at 22°C with gentle shaking. *A. tumefaciens* infection was carried out by dipping the leaf explants in the suspension for 15 min.

Selection of transformed *N. benthamiana* on antibiotic selection media

Leaf explants infected with *A. tumefaciens* as above were briefly blotted on sterile filter paper and then placed on co-cultivation medium [MS media containing 3 % sucrose (w/v), 0.05 mg/L NAA, 0.5 mg/L 6BA (6-Benzylaminopurine), and 0.3 % (w/v) gelzan overlaid with a sterile Whatman filter paper] for 4 days in the dark at 25°C. The explants were then transferred to regeneration medium [MS media containing 3 % sucrose (w/v), 200 mg/L cefotaxime, 50 mg/L kanamycin, 0.05 mg/L NAA, 0.5 mg/L 6BA, and 0.8 % (w/v) gelzan] and incubated at 25°C under a 16h/8h light/dark regime. After 2 weeks, subcultures of the explants were conducted on the same medium, and the concentration of cefotaxime was adjusted to 100 mg/L. After the generated shoots grew up to 2-3 cm, they were excised from the explants and transferred to the rooting medium [1/2 MS medium containing 3% sucrose (w/v), 50 mg/L kanamycin, 0.05 mg/L NAA, 0.02 mg/L IBA and 0.3 % (w/v) gelzan]. After 1 month, the acclimatized transformed plants were transferred and grown on standard greenhouse soil mix.

Chlorophyll and carotenoid analysis

Extraction and determination of total chlorophylls and carotenoids were conducted as previously described[3]. Extracts were obtained in 80% acetone using 100 mg of fresh leaves from transgenic tobacco plants (pooled from 5 plants). After filtration, spectrophotometric quantifications were carried out using a Shimadzu UV-1203 spectrophotometer based on their absorbance at 470.0 nm, 648.6 nm, 664.2 nm.

Effect of MEP and MVA pathway blocking on taxadiene production in transgenic tobacco plants

Transgenic *N. benthamiana* line NTS-T1 seeds were surface-sterilized and sown on half salt Murashige and Skoog (MS) medium[4] containing 3% sucrose (w/v). Sterilized shoot apices that have grown to a length of 1-2 cm from these transgenic lines were transplanted into 1/2MS medium supplemented with either 10 µM lovastatin or 150 µM fosmidomycin. Eight explants were cultured for each treatment. Leaves were harvested after 28 days of growth and extracted for analyses of chlorophylls and carotenoids as described above, and for taxadiene analyses by GC-MS as described in the methods section in the main paper. Whole plants were uprooted and cleaned for phenotype examination.

Supplementary Note 1

NMR analysis

^{1}H-NMR and ^{13}C- NMR spectra were acquired in $CDCl_3$ on a Bruker 500 MHz spectrometer. The spectra were processed with MestReNova software.

taxa-4(5),11(12)-diene (**1**):^{1}H-NMR ($CDCl_3$, 500 MHz) δ 5.27 (1H, m), 2.6 (1H, ddd, J = 15.0, 10.0, 5.5 Hz,), 2.5 (1H, brs), 2.29 (2H, m), 2.10 (1H, m), 2.10-2.00 (2H, m), 1.9-1.65 (6H, m), 1.69 (3H, s), 1.63 (3H, s), 1.40 (1H, ddd, J = 14.8, 5.5, 5.5 Hz,), 1.31 (3H, s), 1.18 (1H, dd, J = 12.7, 6.0 Hz), 1.0 (3H, s), 0.8 (3H, s). ^{13}C-NMR ($CDCl_3$, 125 MHz) δ 138.8, 137.9, 129.8, 121.4, 44.5, 41.6, 40.0, 39.3, 38.7, 37.6, 30.9, 29.6, 28.6, 26.5, 24.7, 24.2, 23.4, 22.9, 21.9, 21.7. This data is consistent with published literature [5,6].

The ^{1}H-NMR of Iso-OCT (**4**) gave three impurity signals at δ7.53 ppm, 7.35 ppm and 7.11 ppm. ^{1}H-NMR ($CDCl_3$, 400 Hz) δ 4.05 (1H, t, J = 5.0 Hz), 2.41 (1H, quint), 2.10 (1H, m), 2.08 (1H, m), 1.85-1.80 (4H, m, overlapped), 1.77-1.65 (4H, m, overlapped), 1.46 (1H, dd, J = 15.2, 3.1 Hz), 1.38-1.37 (3H, m), 1.07 (3H, d, J = 7.2 Hz), 1.02 (3H, s), 1.00 (3H, s), 0.96 (3H, s), 0.90 (3H, s). This data is consistent with published literature[7].

The ^{1}H-NMR spectrum of purified OCT (**3**) showed a mixture of OCT and iso-OCT due to structural instability of OCT. The same impurity can also be found in the spectrum. ^{1}H-NMR ($CDCl_3$, 500 Hz) δ 3.97(1H, dd, J = 9.1, 3.4 Hz), 2.47 (1H, m), 2.21 (1H, dd, J = 12.9, 5.0 Hz), 2.04-1.71 (8H, overlapped), 1.53-1.30 (5H, m overlapped), 1.22 (3H, s), 1.16 (3H, d, J = 6.0 Hz), 1.06 (3H, s), 1.01 (3H, s), 0.95 (3H, s). This data is consistent with published literature[8].

The ^{1}H-NMR of taxadiene-5α-ol (**2**) gave three impurity signals at δ7.71 ppm, 7.54 ppm and 4.31 ppm. ^{1}H-NMR ($CDCl_3$, 500 Hz) δ 4.93 (1H, s), 4.64 (1H, s), 4.25 (1H, t, J = 3.0 Hz), 3.31 (1H, d, J = 4.8 Hz), 2.84 (1H, td, J= 15.0, 5.0 Hz), 2.34 (1H, m), 2.25 (1H, m), 2.10-1.97 (3H, overlapped), 1.89 (1H, m), 1.83 (3H, s), 1.80-1.62 (5H, overlapped), 1.34 (3H, s), 1.04 (3H, s), 0.61 (3H, s). This data is consistent with published literature[7].

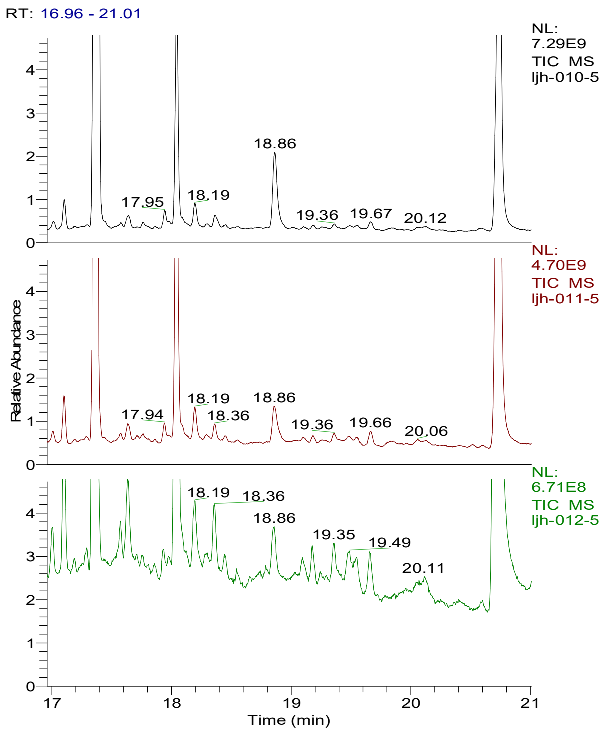

Supplementary Figure 1. **Constitutive production of taxa-4(5),11(12)-diene (1) in the hexane extracts of *N. benthamiana* leaves.** Top panel, 5 dpi (days after infiltration); Middle panel, 4 dpi; Bottom panel, 2 dpi. The peak at retention time 18.86 min was identified as taxa-4(5),11(12)-diene (**1**).

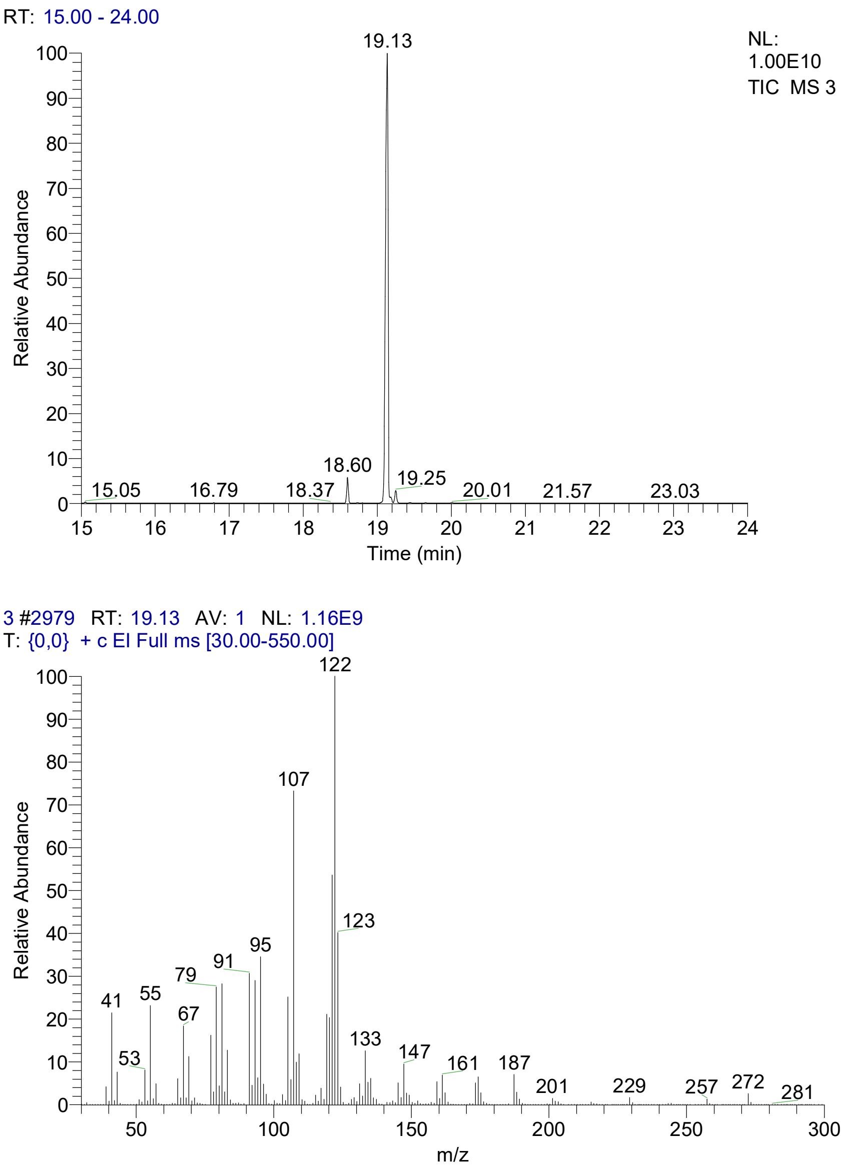

Supplementary Figure 2. **GC-MS analysis of purified taxa-4(5),11(12)-diene (1).** Top panel shows the TIC of purified **1**. The peak at retention time 19.1 min corresponds to **1**. The lower panel shows the mass spectrum of **1**. The retention time of 1 was delayed by 0.3 min due to column regeneration.

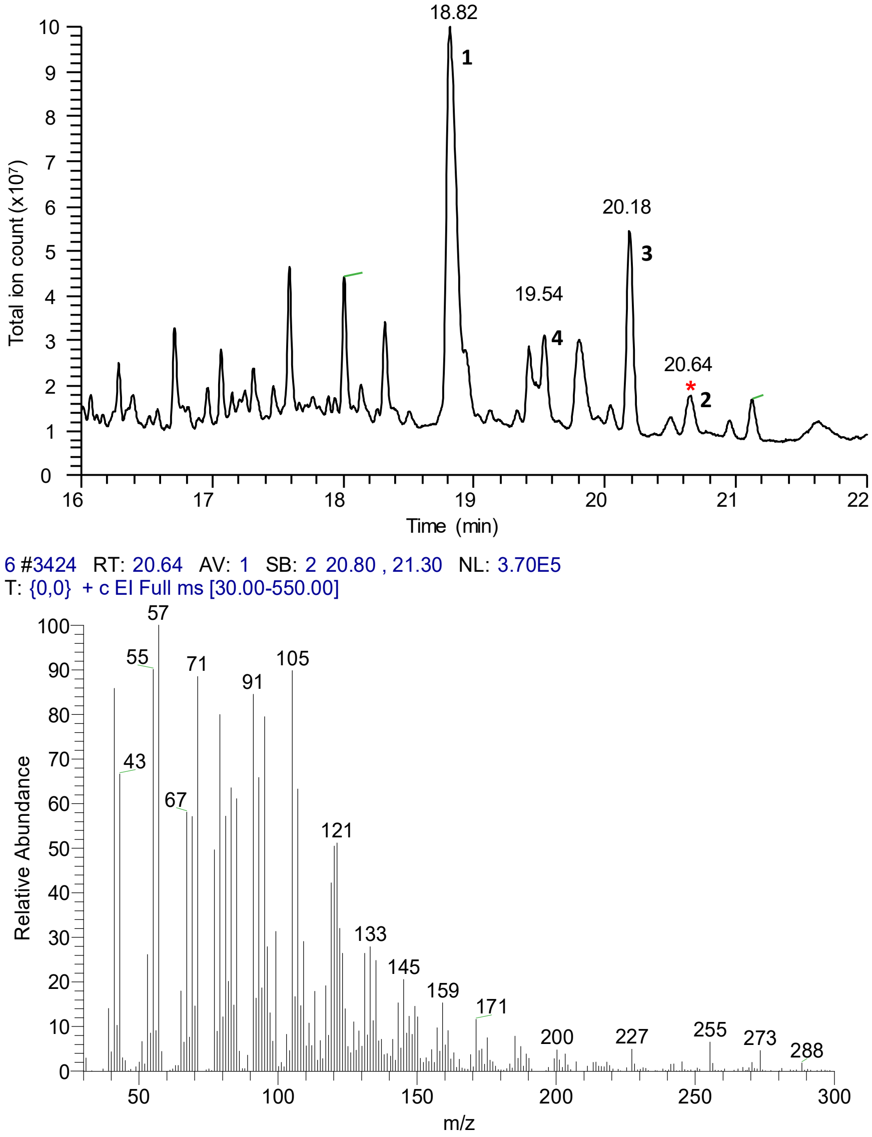

Supplementary Figure 3. **GC-MS analysis of taxadiene-5α-ol.** Top panel shows the total ion chromatogram (TIC) of hexane/ ethyl acetate (4:1) extracts from DXS-GGPPS and TS-tp(TS)/trT5H/trCPR co-transient *N. benthamiana* leaves. The peak at retention time 20.6 min corresponds to taxadiene-5α-ol (**2**). The lower panel shows the mass spectrum of **2**.

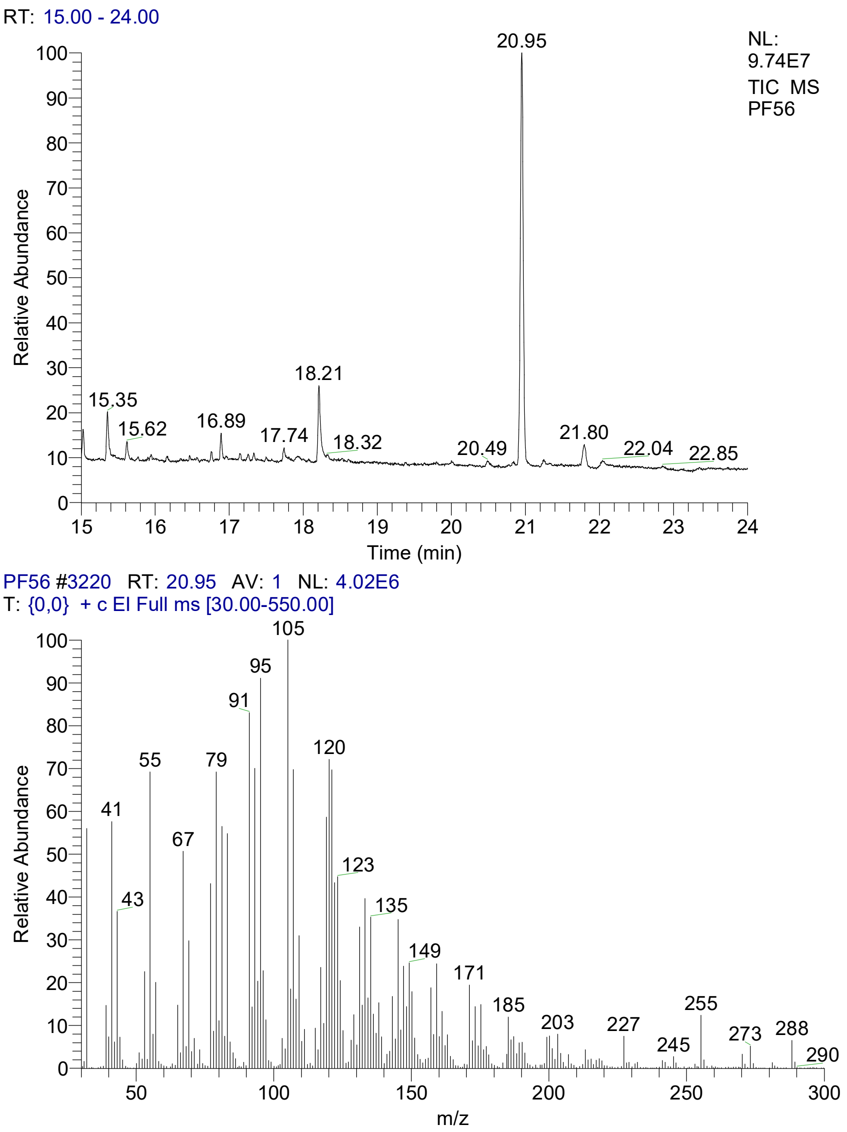

Supplementary Figure 4. GC-MS analysis of purified taxadiene-5α-ol (2). Top panel shows the TIC of purified **2**. The peak at retention time 20.9 min corresponds to desired compound. The lower panel shows the mass spectrum of **2**. The retention time of **2** was delayed by 0.3 min due to column regeneration.

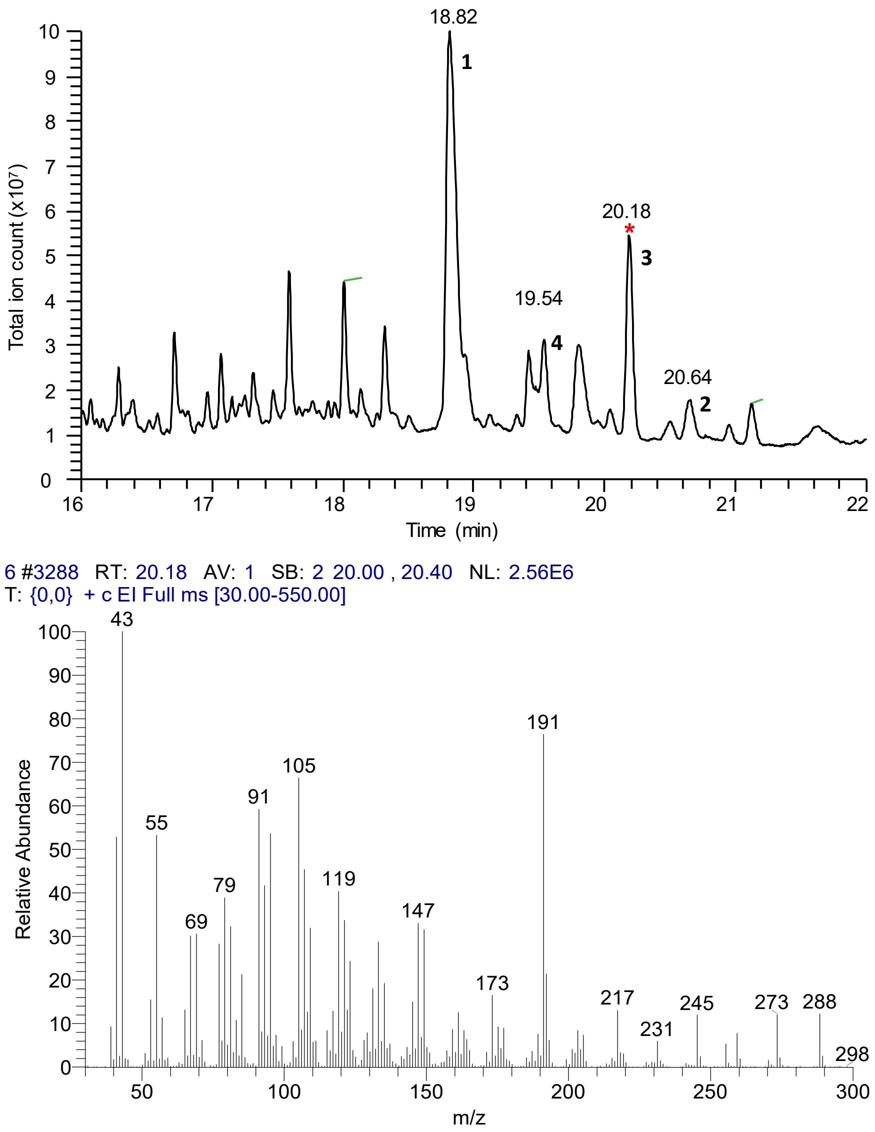

Supplementary Figure 5. **GC-MS analysis of 5(12)-oxa-3(11)-cyclotaxane (OCT).** Top panel shows the total ion chromatogram (TIC) of hexane/ ethyl acetate (4:1) extracts from DXS-GGPPS and TS-tp(TS)/trT5H/trCPR co-transient *N. benthamiana* leaves. The peak at retention time 20.2 min corresponds to cyclic-cyclotaxane (OCT, **3**). The lower panel shows the mass spectrum of **3**.

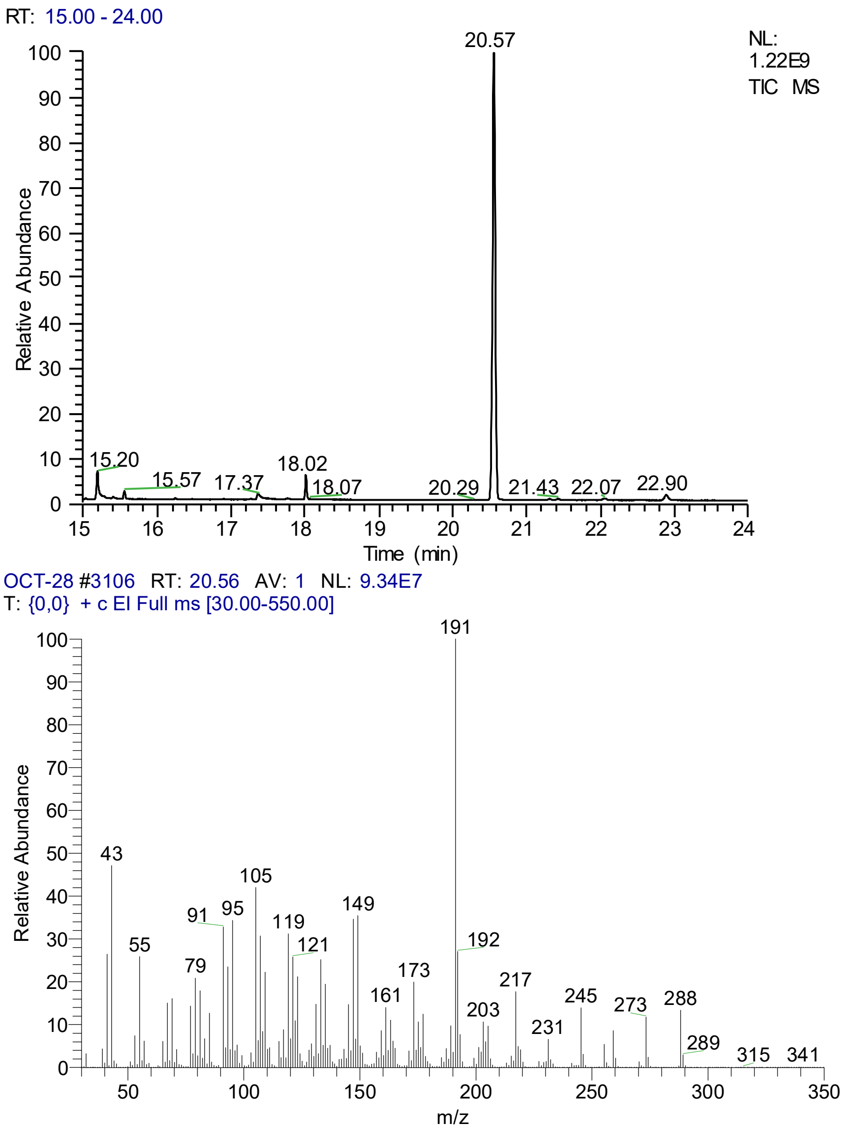

Supplementary Figure 6. GC-MS analysis of purified OCT. Top panel shows the TIC of purified OCT and the peak at retention time 20.5 min corresponds to desired compound. The lower panel shows the mass spectrum of **3**. The retention time of **3** was delayed by 0.4 min due to column regeneration.

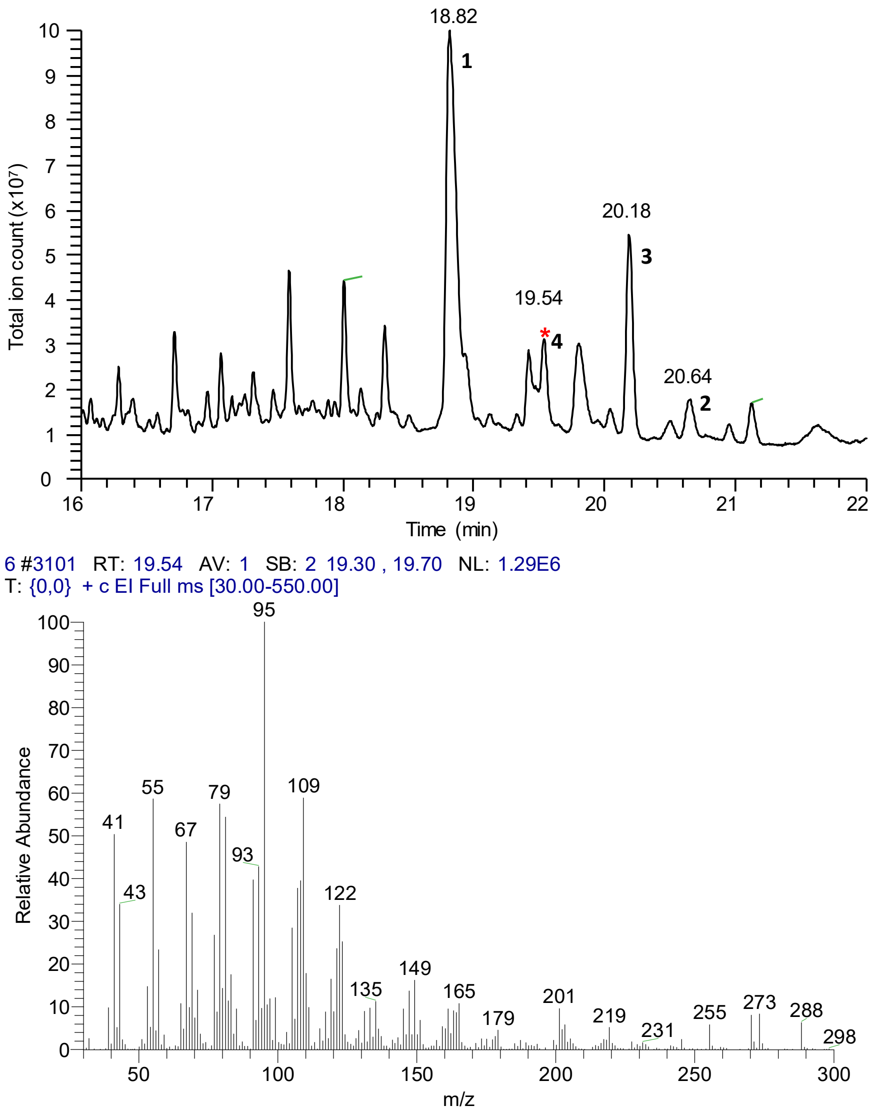

Supplementary Figure 7. GC-MS analysis of 5(13)-oxa-3(11)-cyclotaxane (iso-OCT). The Top panel shows the total ion chromatogram (TIC) of hexane/ ethyl acetate (4:1) extracts from DXS-GGPPS and TS-tp(TS)/trT5H/trCPR co-transient *N. benthamiana* leaves. The peak at retention time 19.5 min corresponds to iso-OCT (**4**). The bottom panel shows the mass spectrum of iso-OCT **4**.

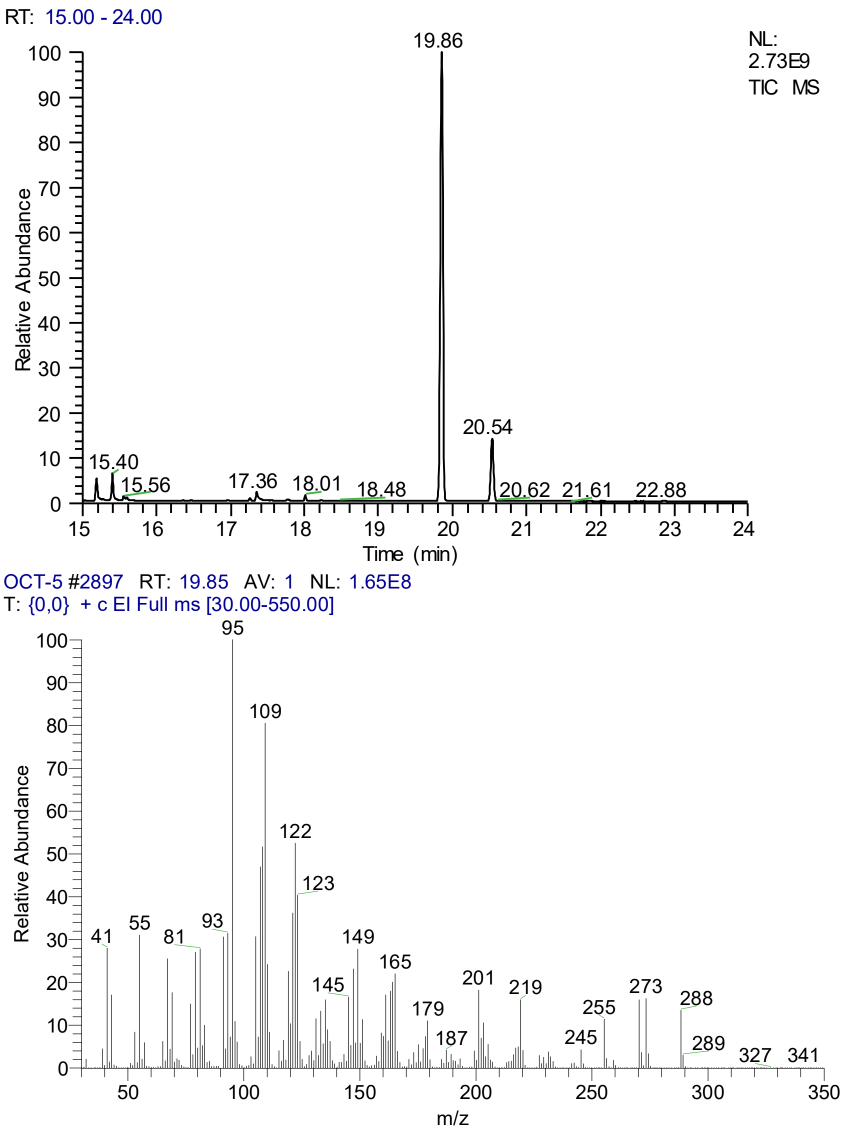

Supplementary Figure 8. **GC-MS analysis of purified iso-OCT.** Top panel shows the TIC of purified iso-OCT and the peak at retention time 19.8 min corresponds to desired compound. The lower panel shows the mass spectrum of **4**. The retention time of **4** was delayed by 0.3 min due to column regeneration.

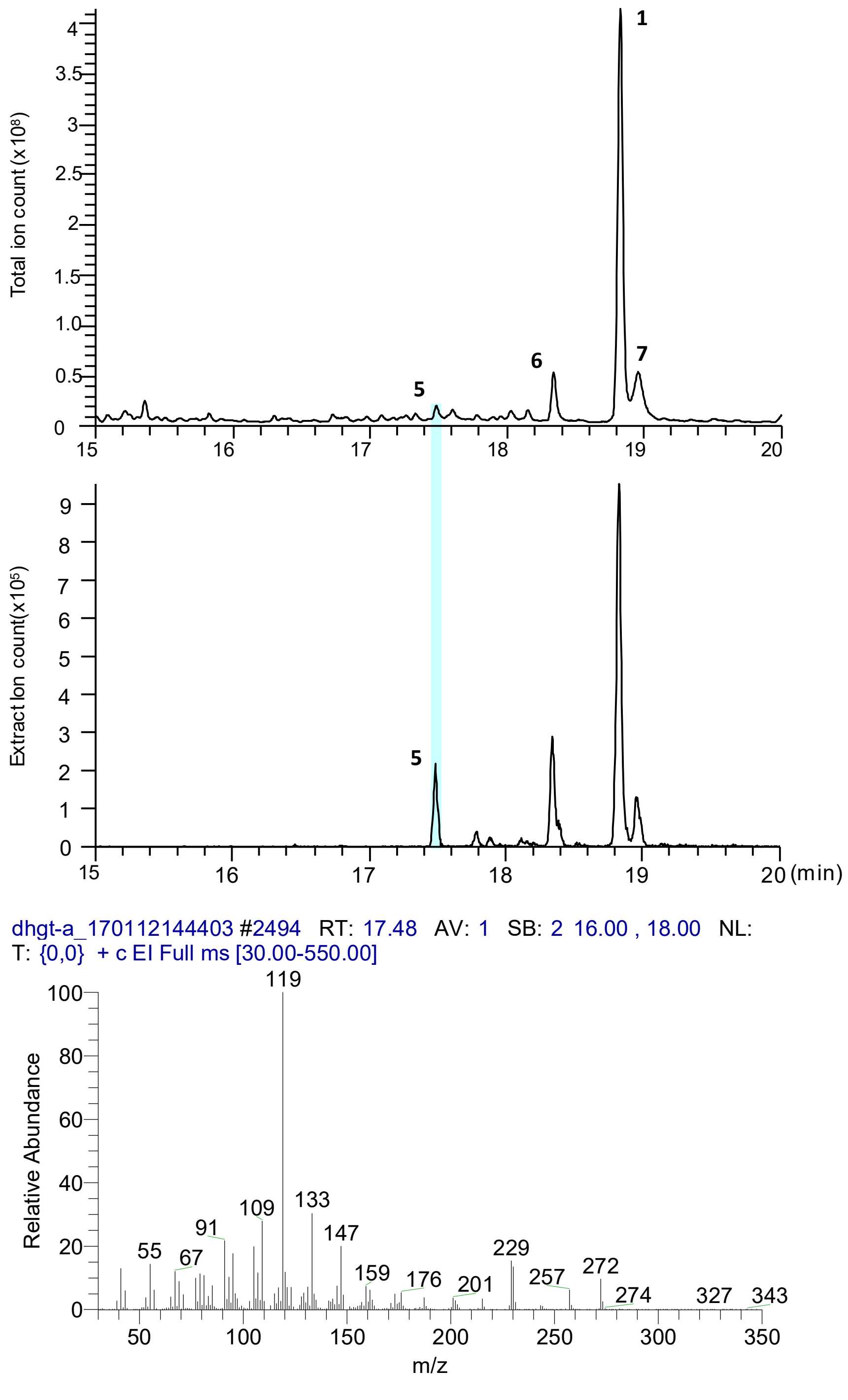

Supplementary Figure 9. GC-MS analysis of verticillene. Top panel shows the total ion chromatogram (TIC) of hexane extracts from DXS-GGPPS-TS transient *N. benthamiana* leaves, the middle panel shows the extracted ion chromatogram (EIC) for m/z 272, the bottom panel shows the mass spectra of verticillene (**5**) at retention time 17.5 min.

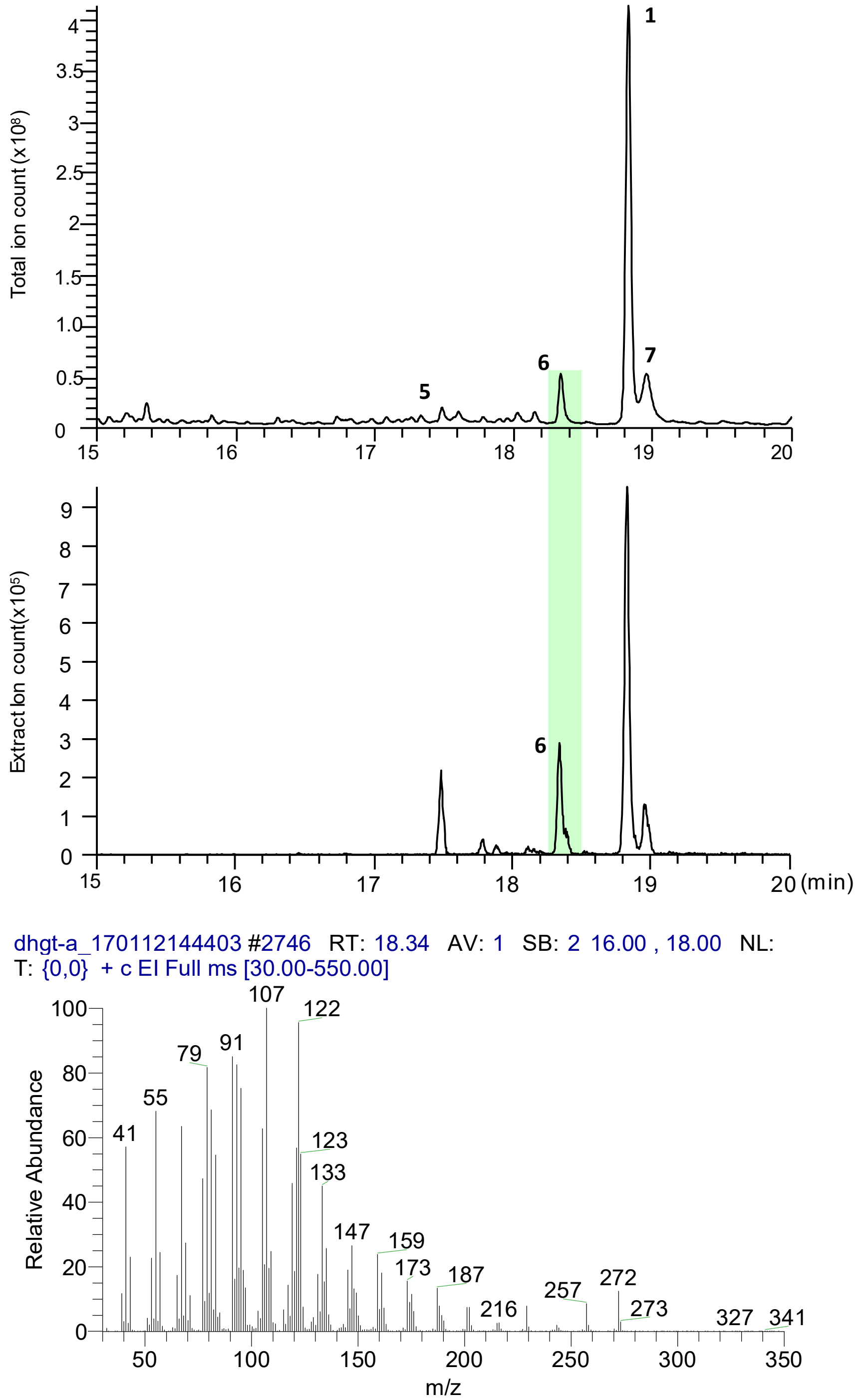

Supplementary Figure 10. GC-MS analysis of taxa-4(20),11(12)-diene. The peak at the retention time 18.3 min corresponds to taxa-4(20),11(12)-diene (**6**). The middle panel shows the EIC and the bottom panel shows the mass spectrum of **6**.

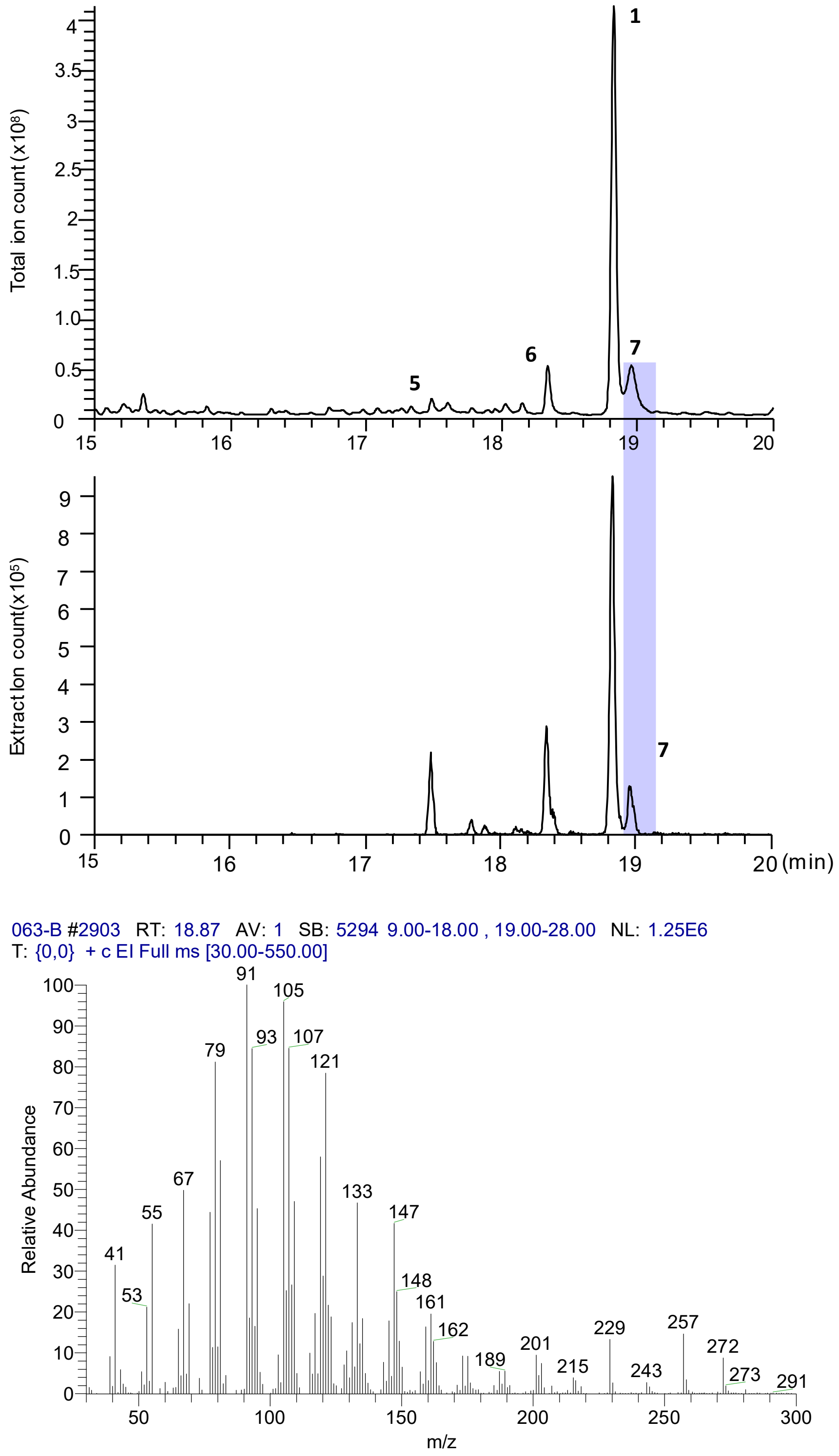

Supplementary Figure 11. GC-MS analysis of taxa-3(4),11(12)-diene. The peak at the retention time 18.9 min corresponds to taxa-3(4),11(12)-diene (**7**). The middle panel shows the EIC and the bottom panel shows the mass spectrum of **7**.

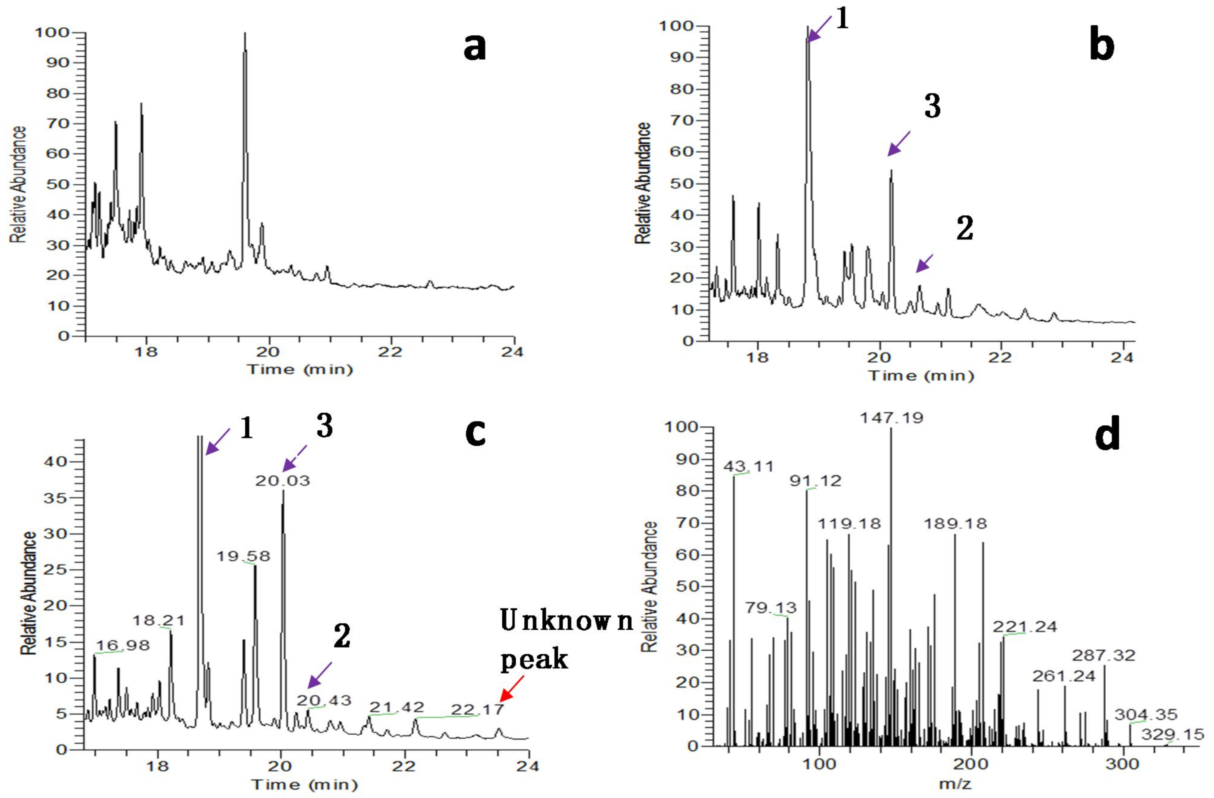

Supplementary Figure 12. **GC-MS analysis of GV3101 harboring pEAQ-TS-tp(TS)/trT5H-trCPR/tp(TS)T5A/tp(TS)trT10H and DXS-GGPPS co-infiltrated leaf extracts.** (**a**) GC-MS spectrum of control; (**b**) GV3101 harboring pEAQ-TS-tp(TS)/trT5H-trCPR co-expressed with DXS-GGPPS; (**c**) GV3101 harboring pEAQ-TS-tp(TS)/trT5H-trCPR/tp(TS)T5A/tp(TS)trT10H co-expressed with DXS-GGPPS; (**d**) Mass spectrum of the unknown peak with retention time at 23.5 min in **c**.

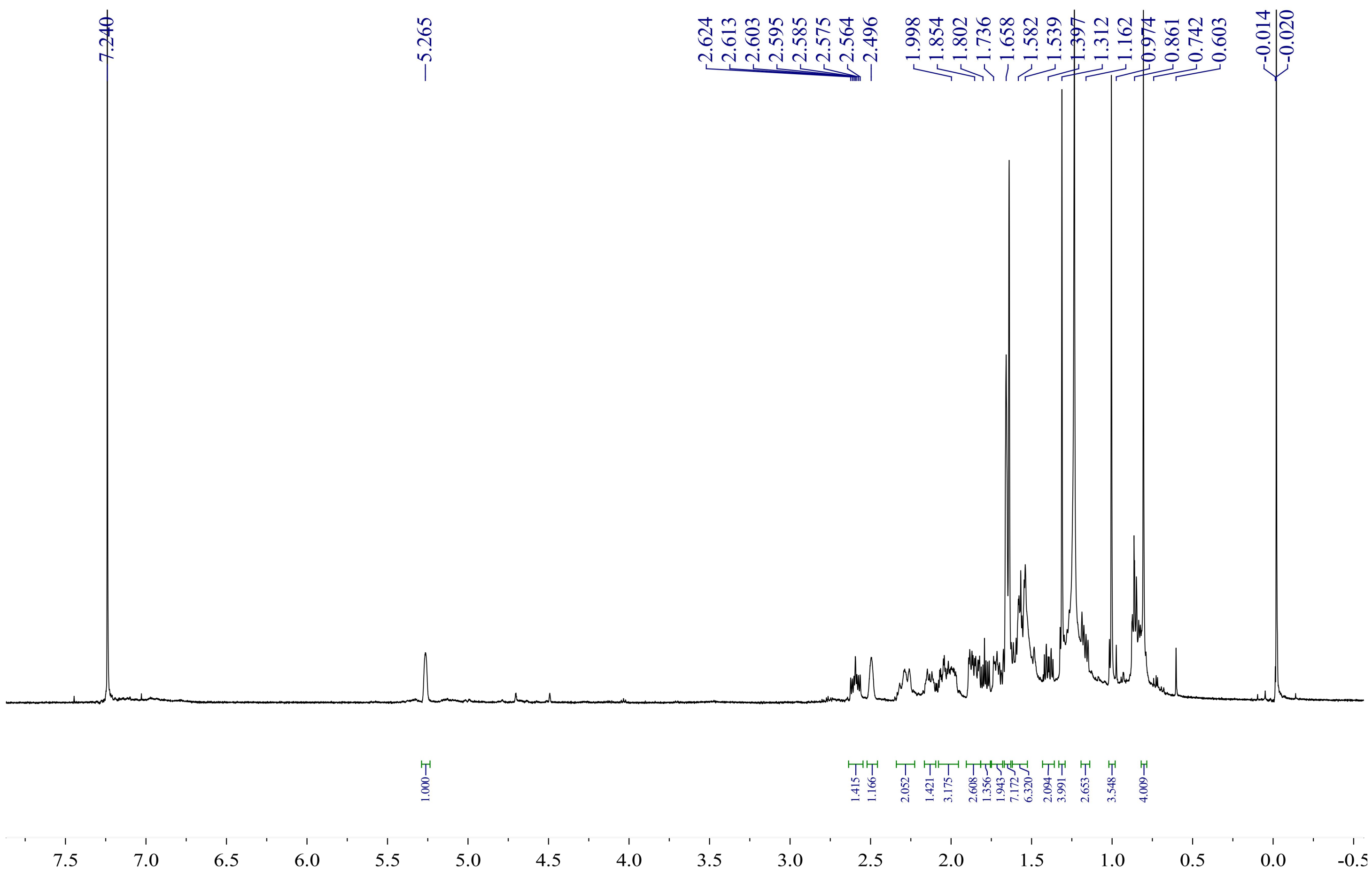

Supplementary Figure 13. **^{1}H-NMR spectrum of purified taxa-4(5),11(12)-diene (1) from transient plant tissues.**

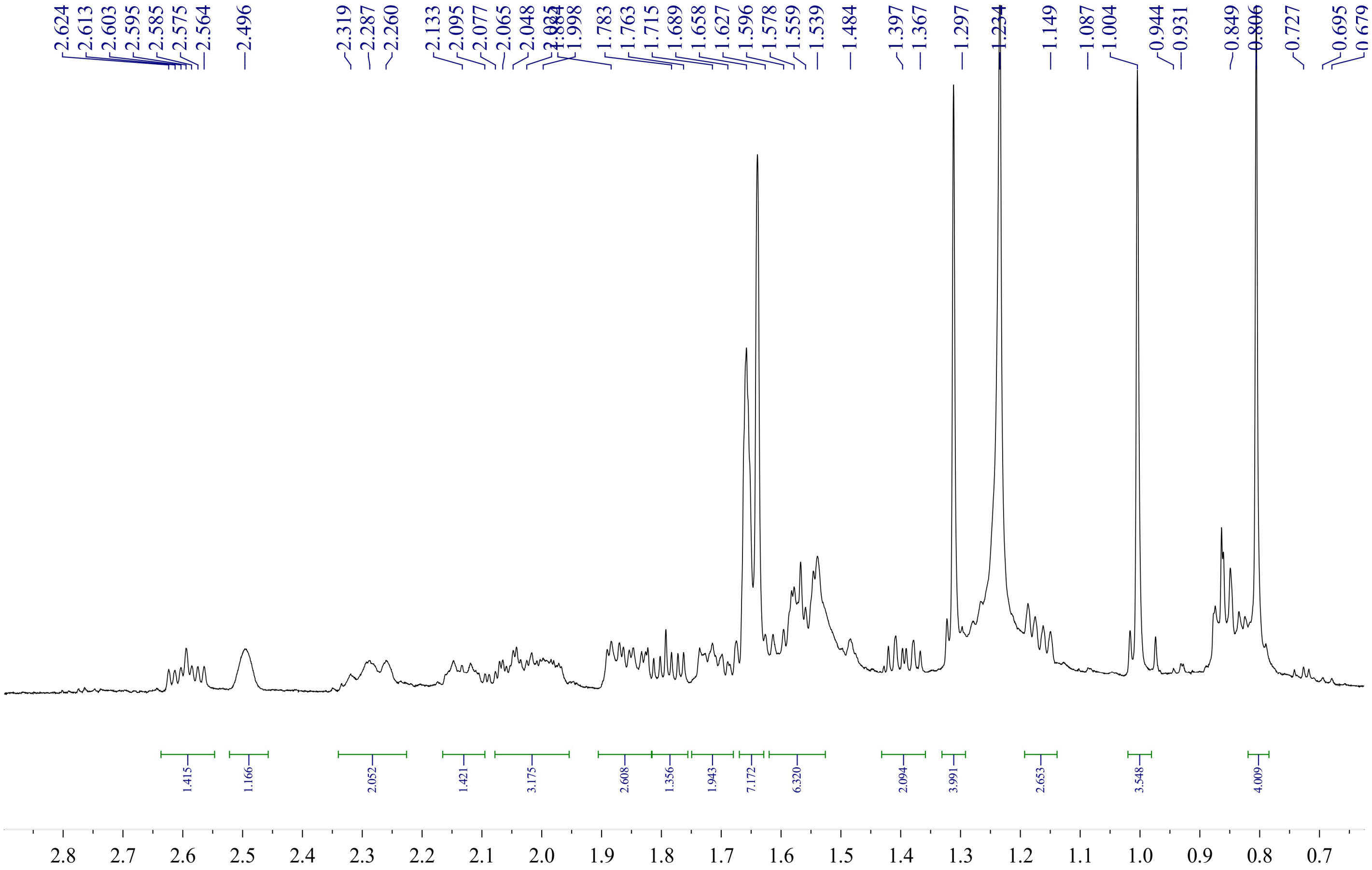

Supplementary Figure 14. Part of ^{1}H-NMR spectrum of purified taxa-4(5),11(12)-diene (1) from transient plant tissues.

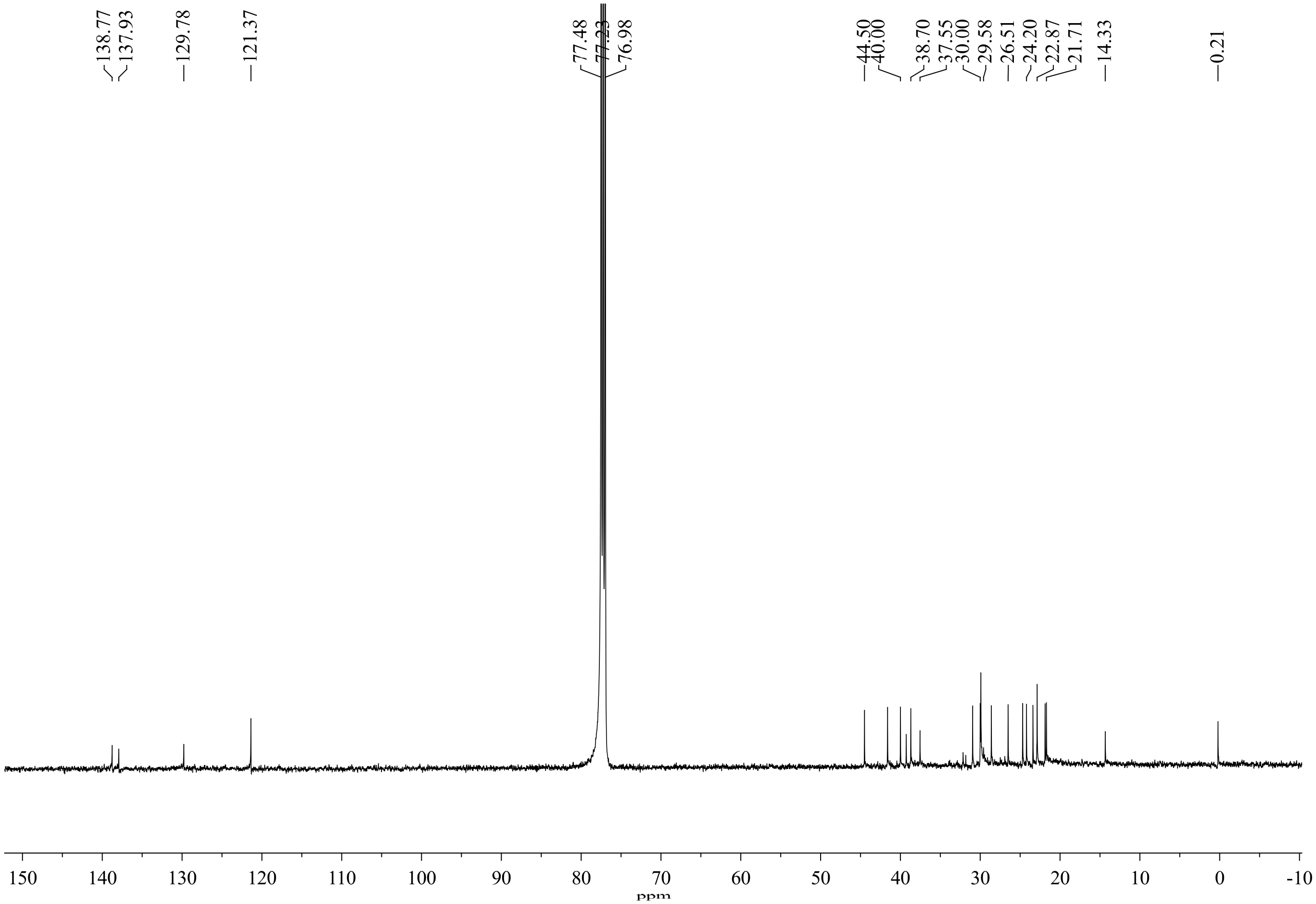

Supplementary Figure 15. ^{13}C-NMR spectrum of purified taxa-4(5),11(12)-diene (1) from transient plant tissues.

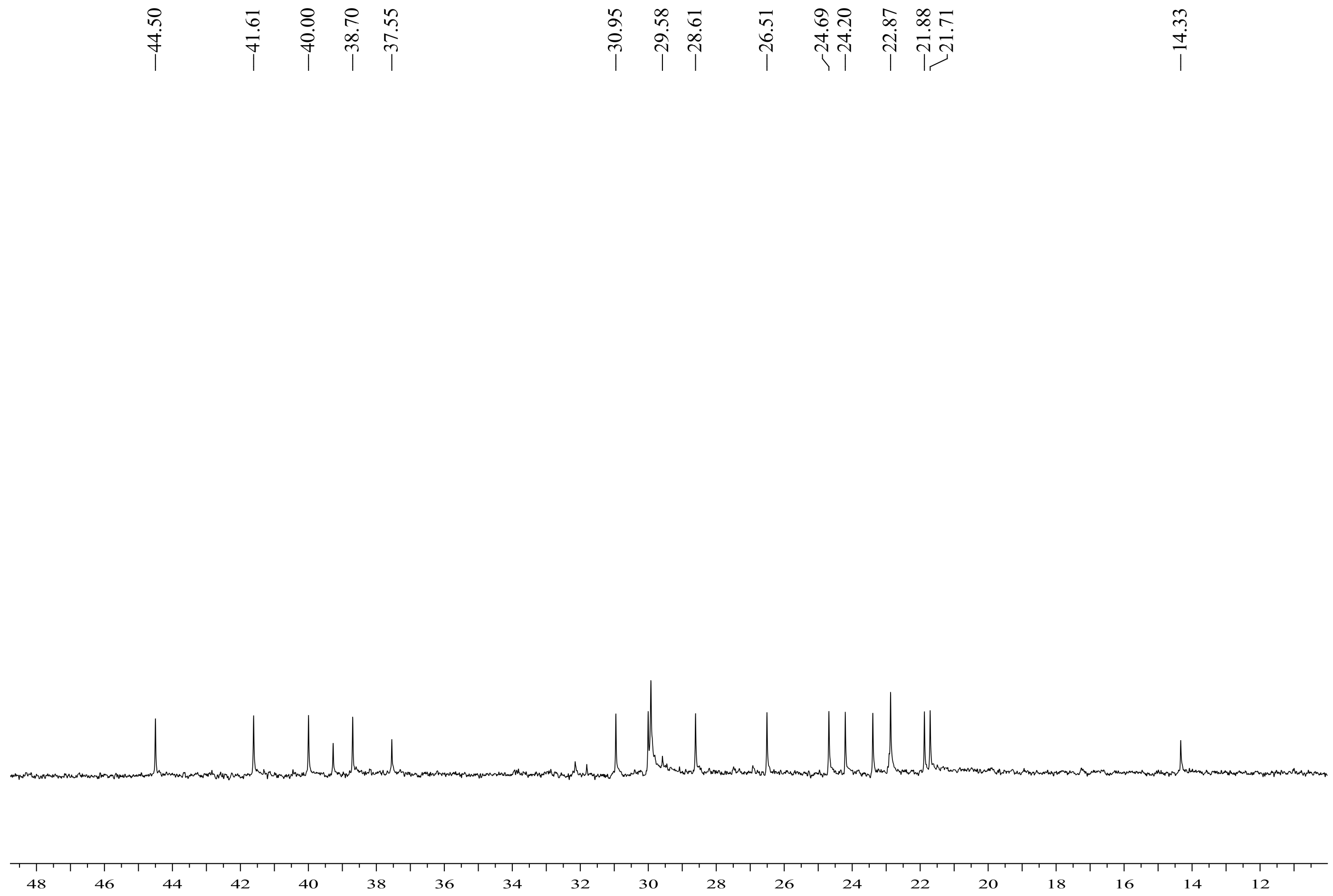

Supplementary Figure 16. Part of 13C-NMR spectrum of purified taxa-4(5),11(12)-diene (1) from transient plant tissues.

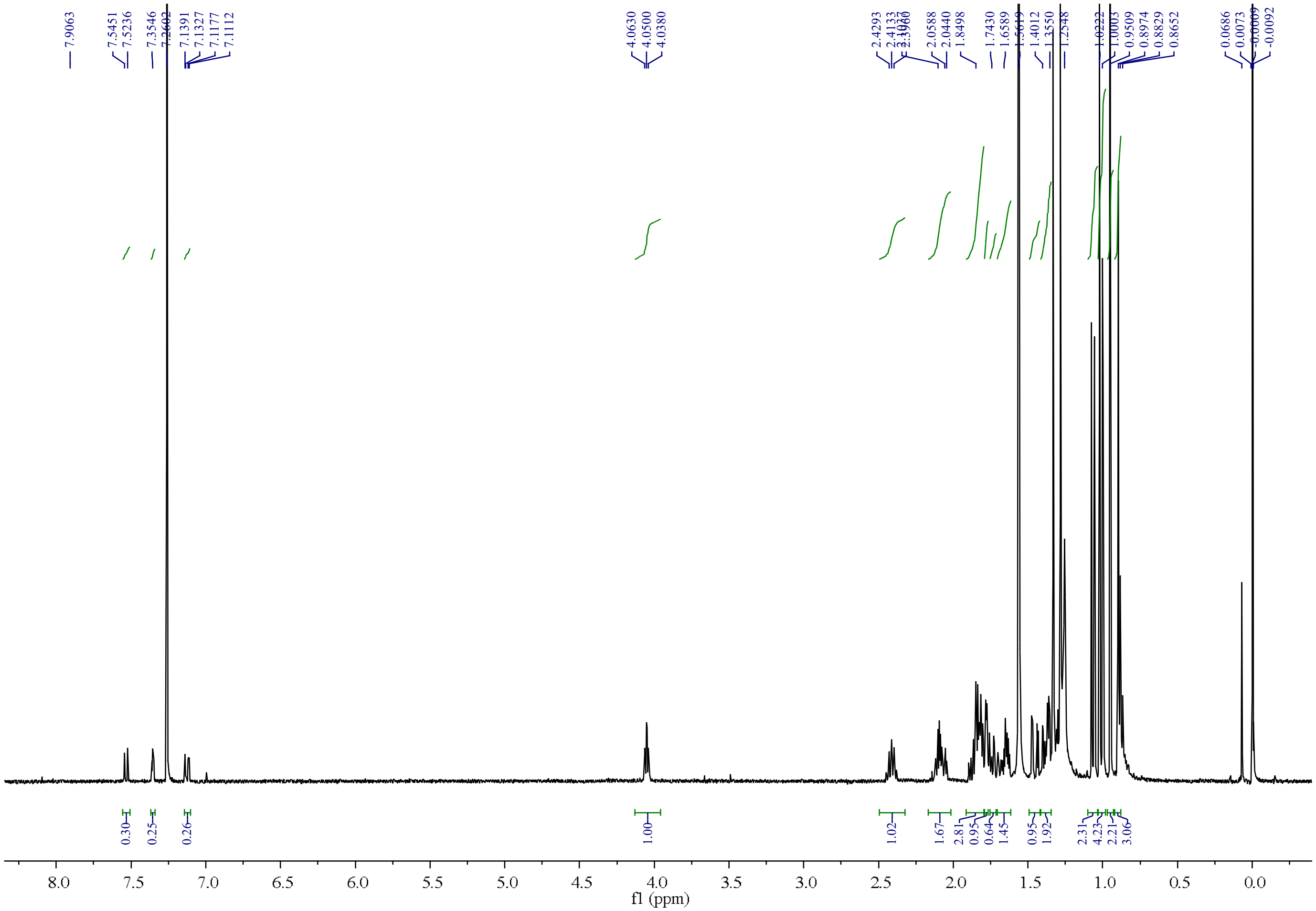

Supplementary Figure 17. **^{1}H-NMR spectrum of purified iso-OCT (4) from transient plant tissues.**

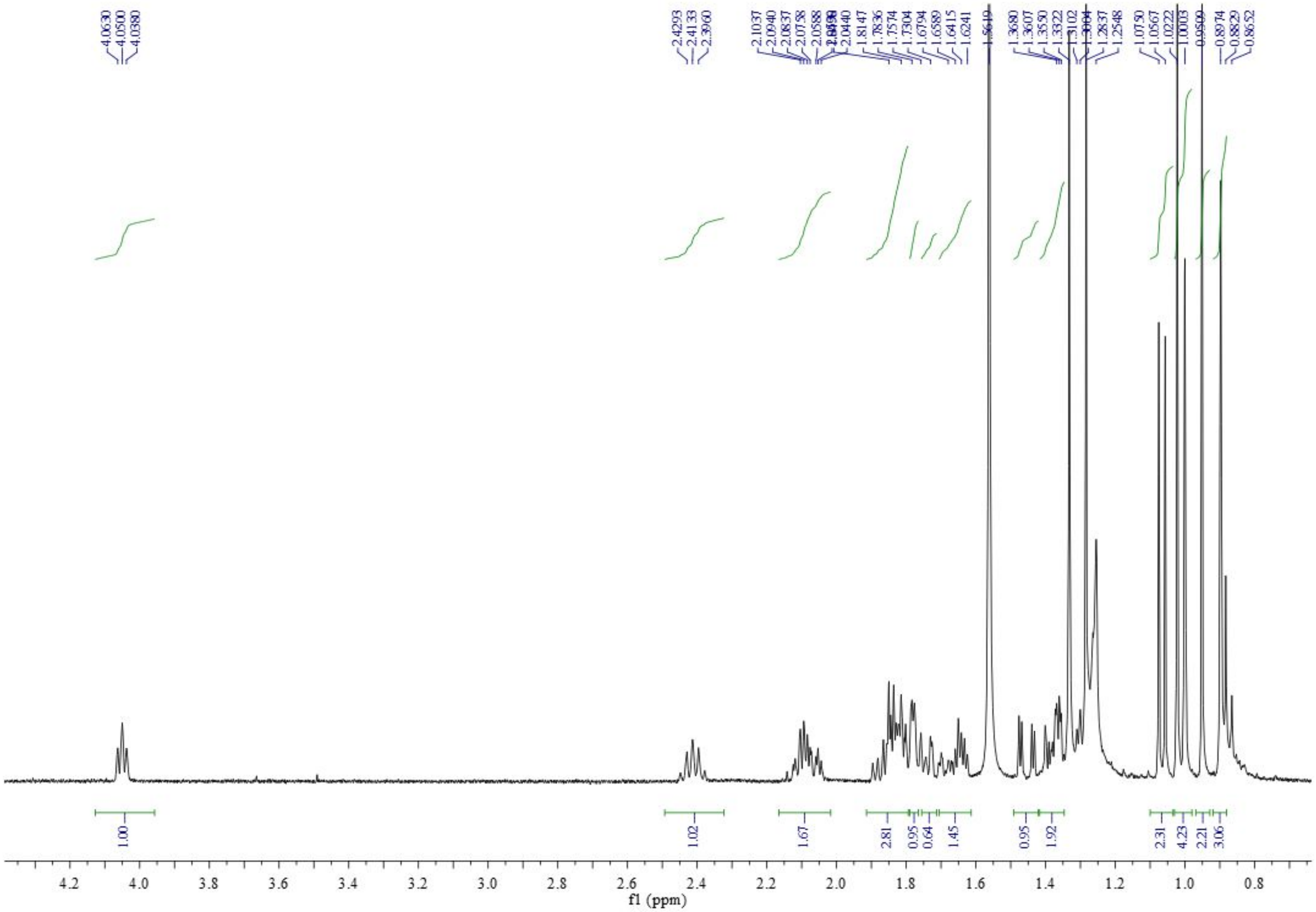

Supplementary Figure 18. **Part of ^{1}H-NMR spectrum of purified iso-OCT (4) from transient plant tissues.**

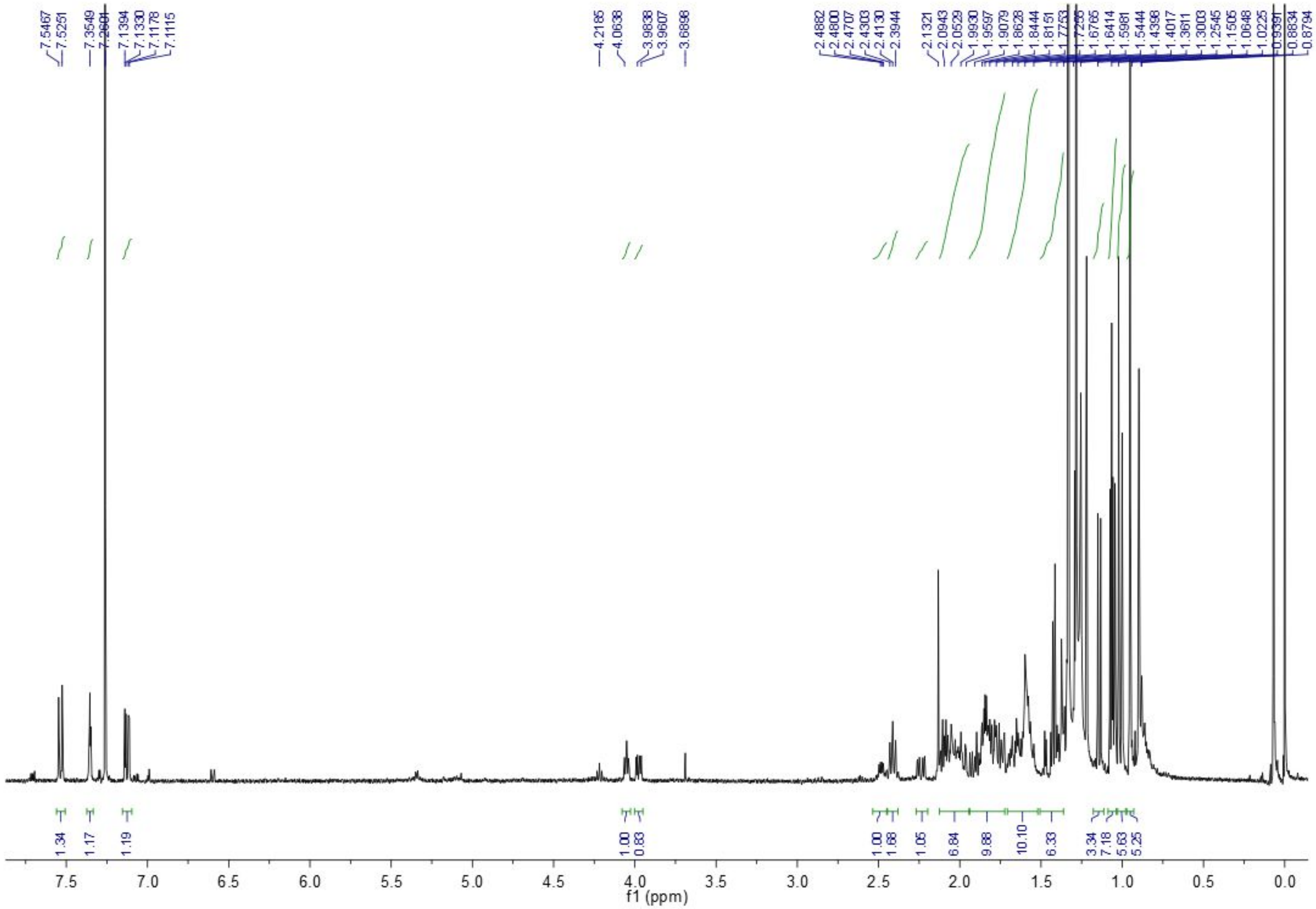

Supplementary Figure 19. ^{1}H-NMR spectrum of purified OCT (3) from transient plant tissues.

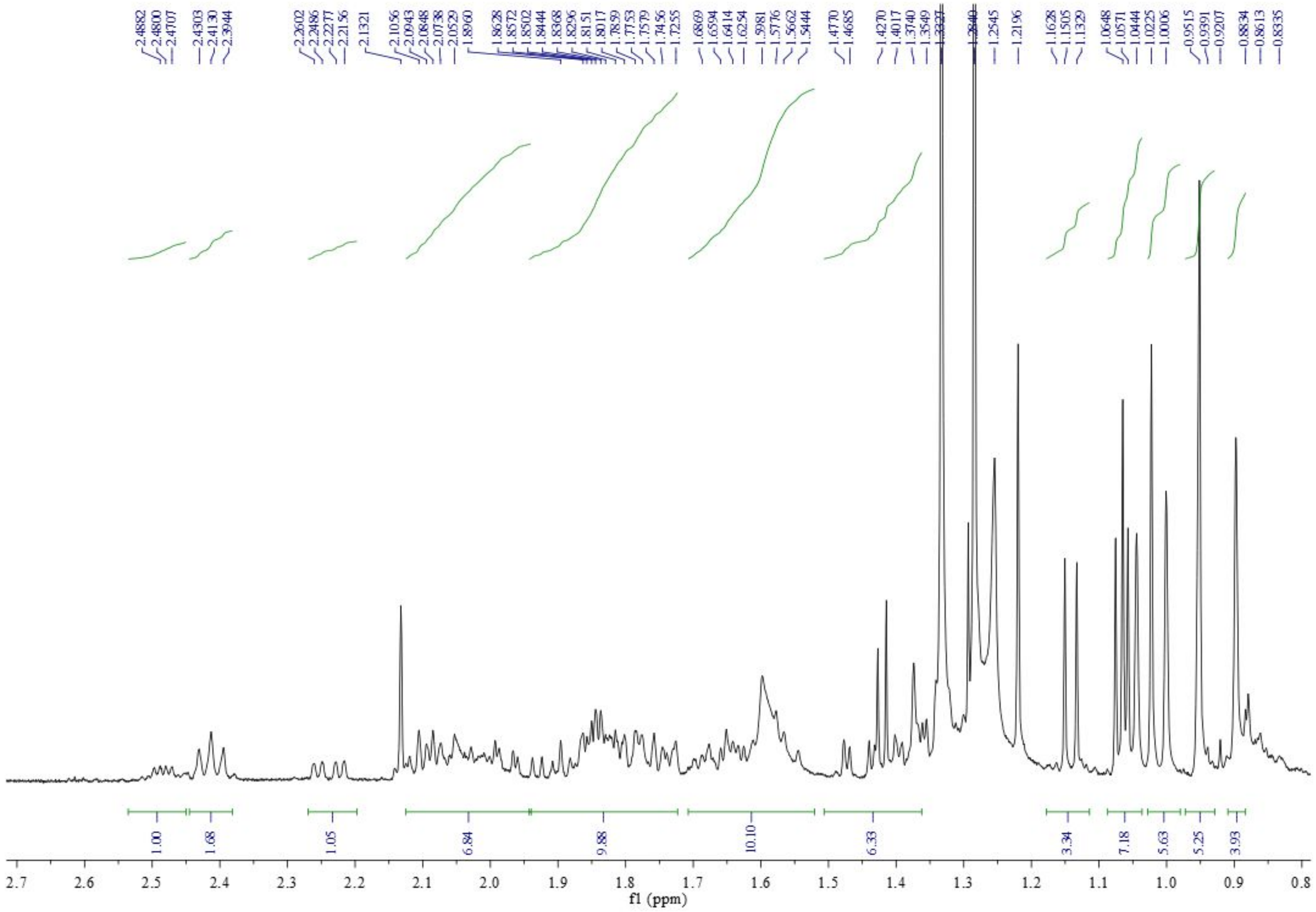

Supplementary Figure 20. Part of ^{1}H-NMR spectrum of purified OCT (3) from transient plant tissues.

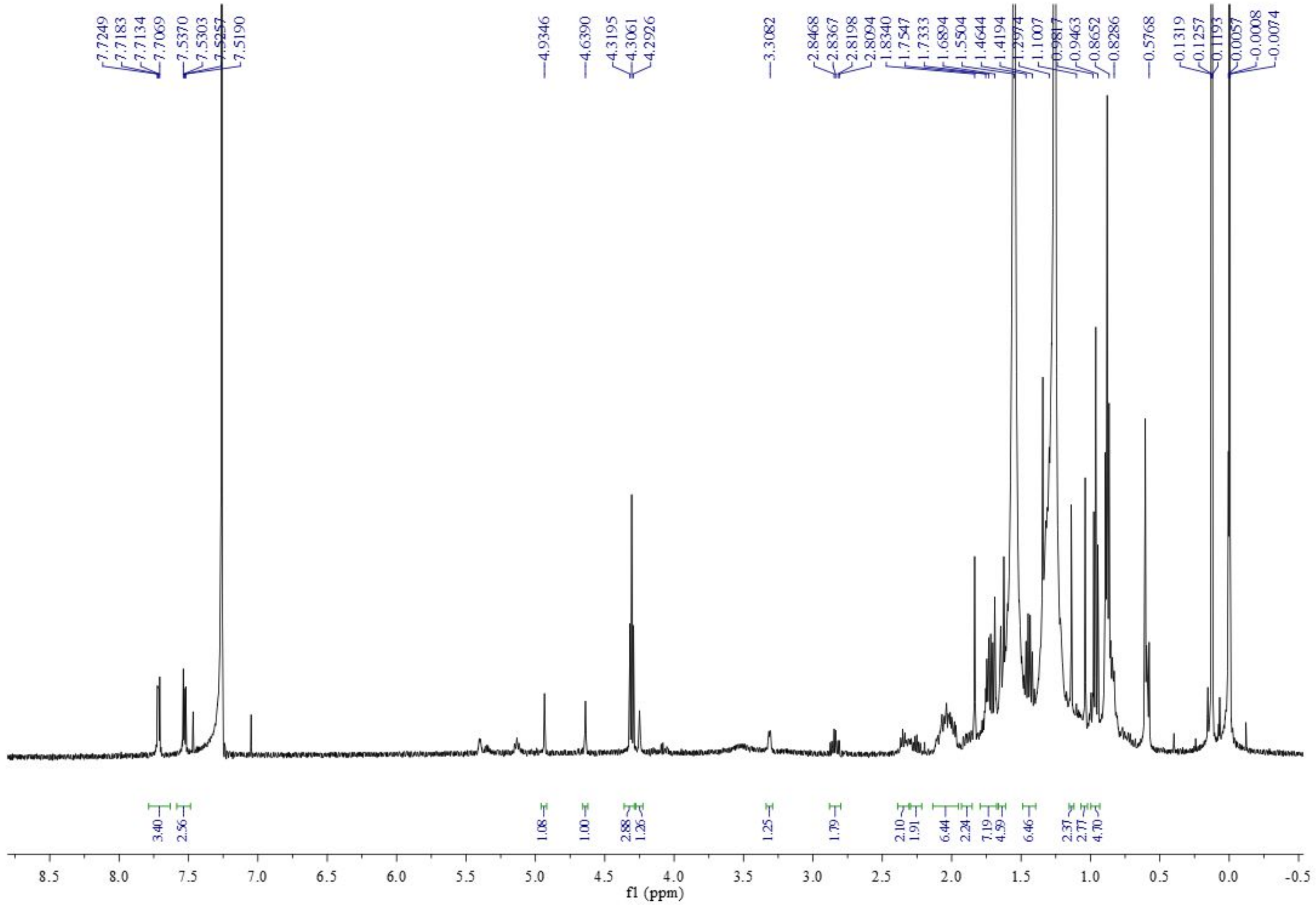

Supplementary Figure 21. ^{1}**H-NMR spectrum of purified taxadiene-5a-ol (2) from transient plant tissues.**

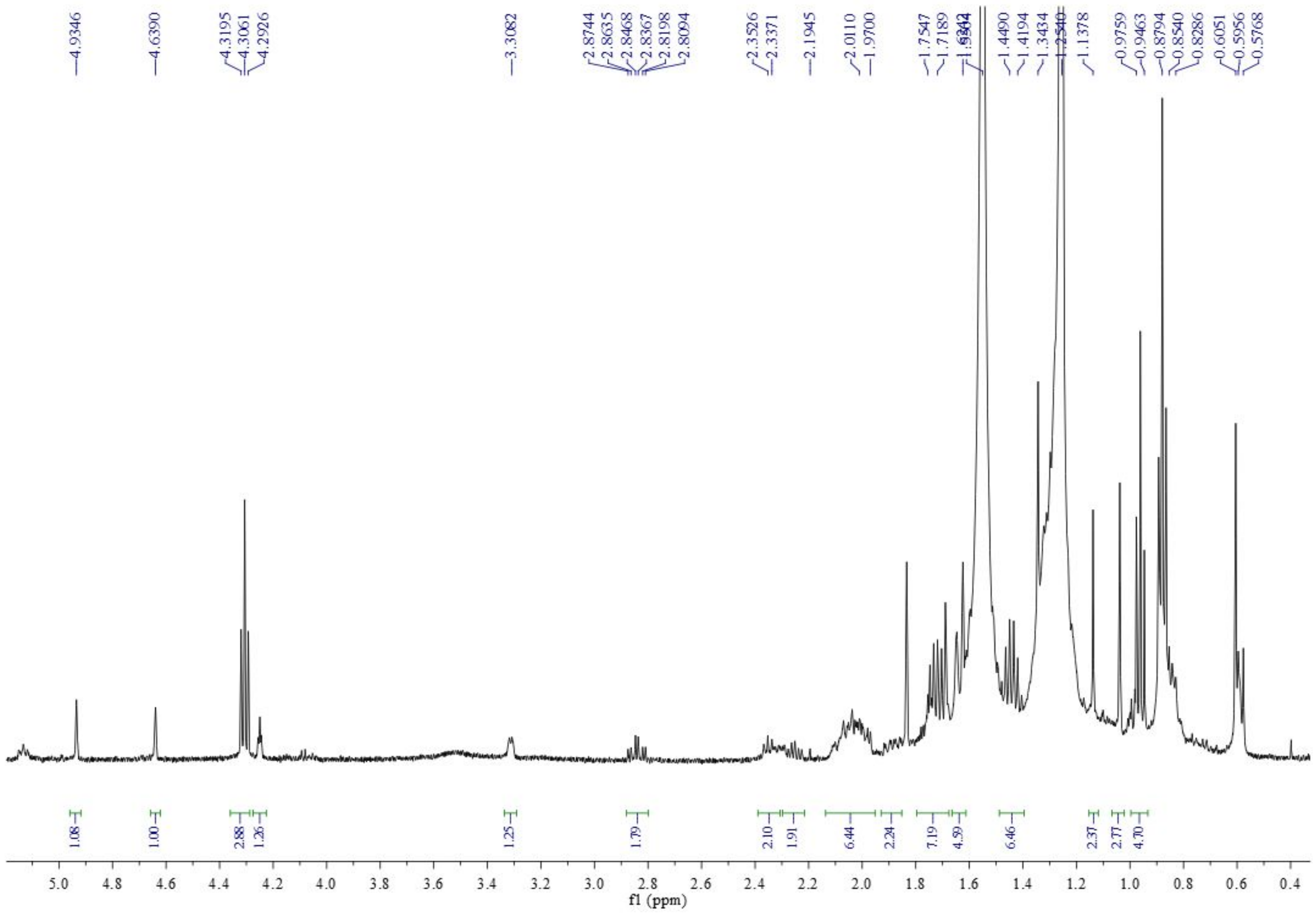

Supplementary Figure 22. **^{1}H-NMR spectrum of purified taxadiene-5a-ol (2) from transient plant tissues.**

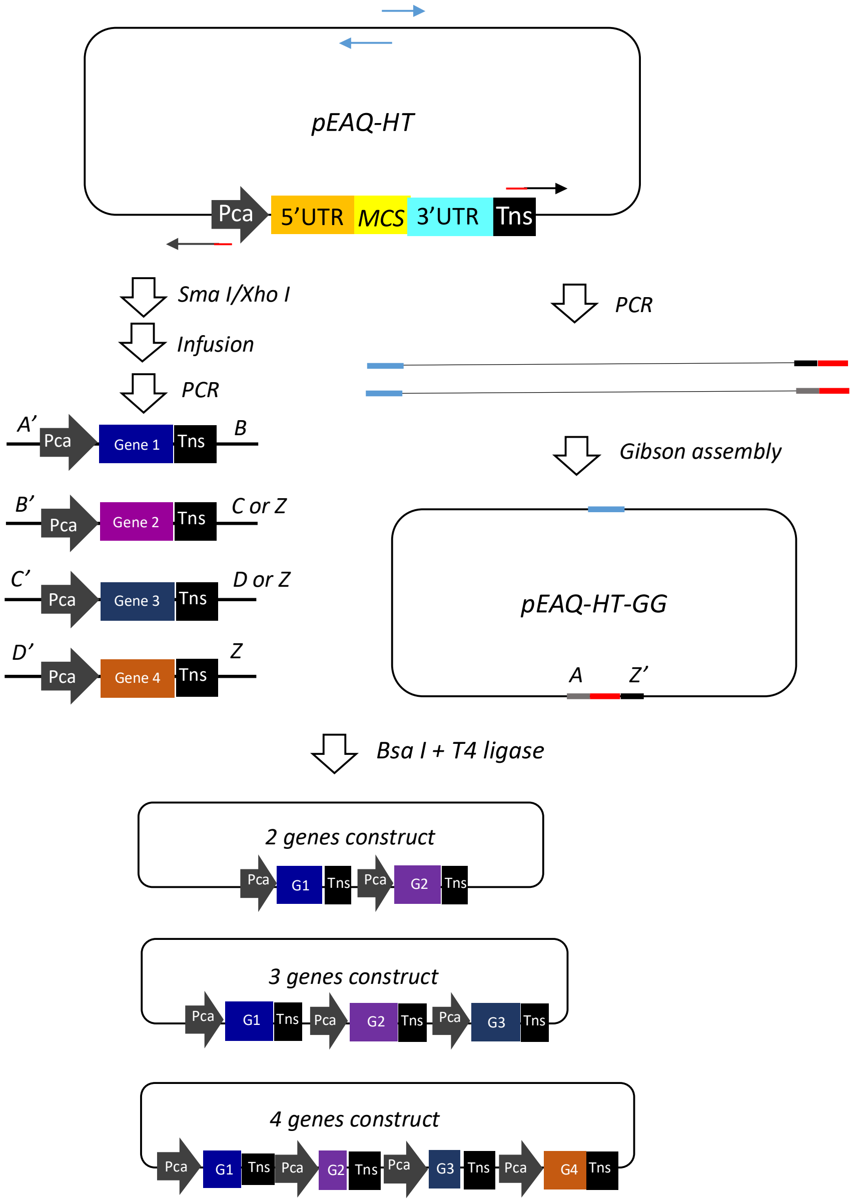

Supplementary Figure 23. Golden gate assembly strategy for multiple-cassettes constructs for transient expression in *N. benthamiana*. Two sets of primers were used to PCR amplify the empty plasmid pEAQ-HT. Bold black and gray sections of the primers bind to plasmid and also contain Bsa I sites that when cut give unique 4 bp overhangs. Blue and red sections of the primers contain overlap sequences. The two amplified regions were combined by Gibson assembly giving pEAQ-HT-GG. For single gene construct, pEAQ-HT were digested by Sma I and Xho I, then ligated with digested PCR fragments. For multi-gene construct, gene sequences of interest were PCR-amplified from pEAQ-HT constructs to include promoter, 5'UTR, coding sequence, 3'UTR and terminator regions. Primers were designed to add a Bsa I site with a unique 4 bp overhang when digested. Overhang region A is complementary to overhang region B, and so on. Amplicons and pEAQ-HT-GG were then assembled by Golden gate assembly creating the two genes, three genes, and four genes constructs. Pca, Cauliflower Mosiac Virus 35S promoter; Tns, Nopaline synthase terminator; MCS, multiple cloning site; 5'UTR, 5' untranslated region; 3'UTR, 3' untranslated region.

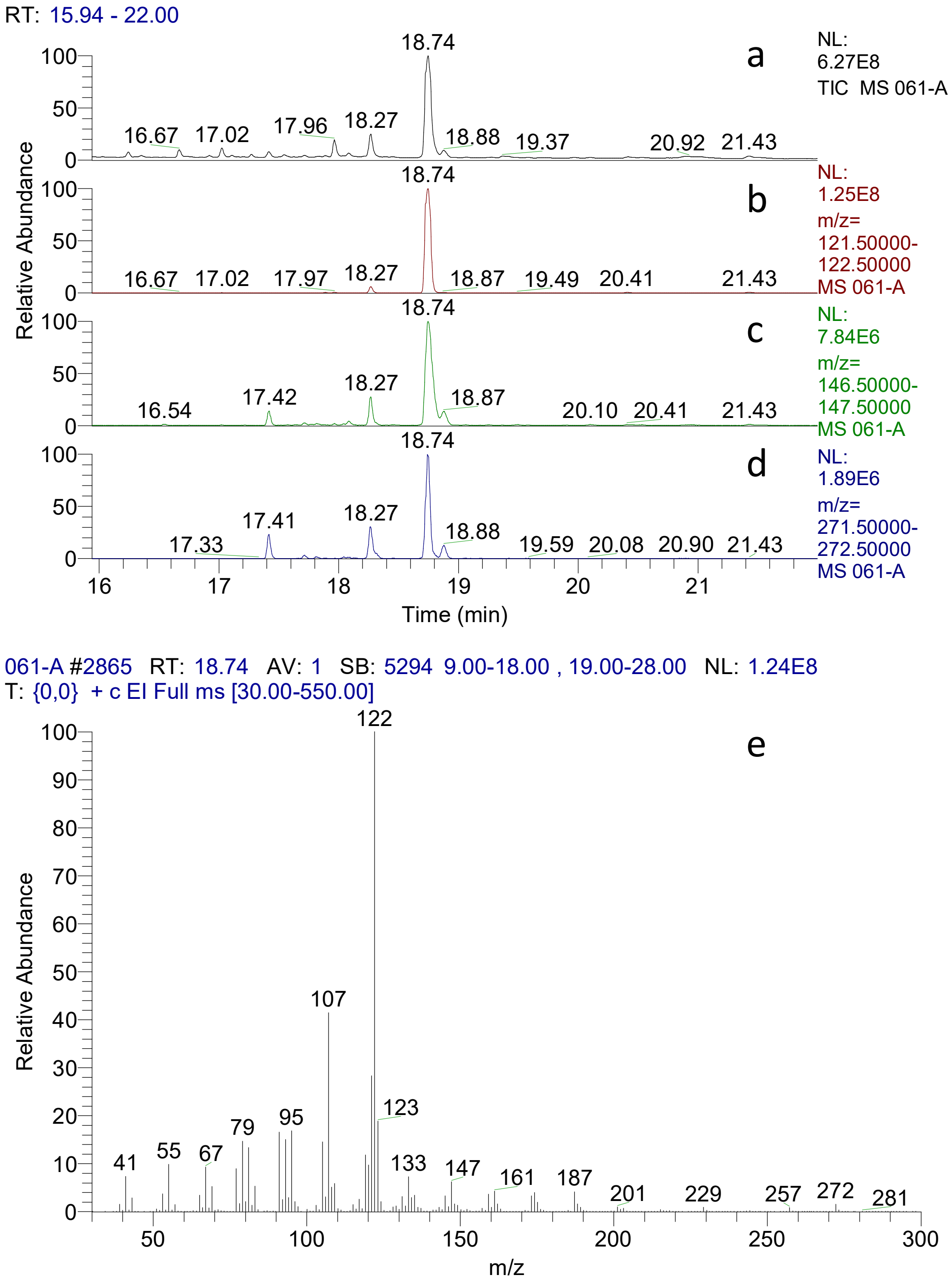

Supplementary Figure 24. GC-MS analysis of taxadiene with transient co-expression of DXS and TS in *N. benthamiana* leaves. (a) TIC of hexane extract of DXS and TS transient co-expression leaves; (b) EIC for m/z^+ 122 representing taxa-4(5), 11(12)-diene; (c) EIC for m/z^+ 147 representing a putative unknown compound; (d) EIC for m/z^+ 272 representing taxadiene and its analogs; (e) Mass spectrum of peak (tR 18.74) representing taxa-4(5),11(12)-diene.

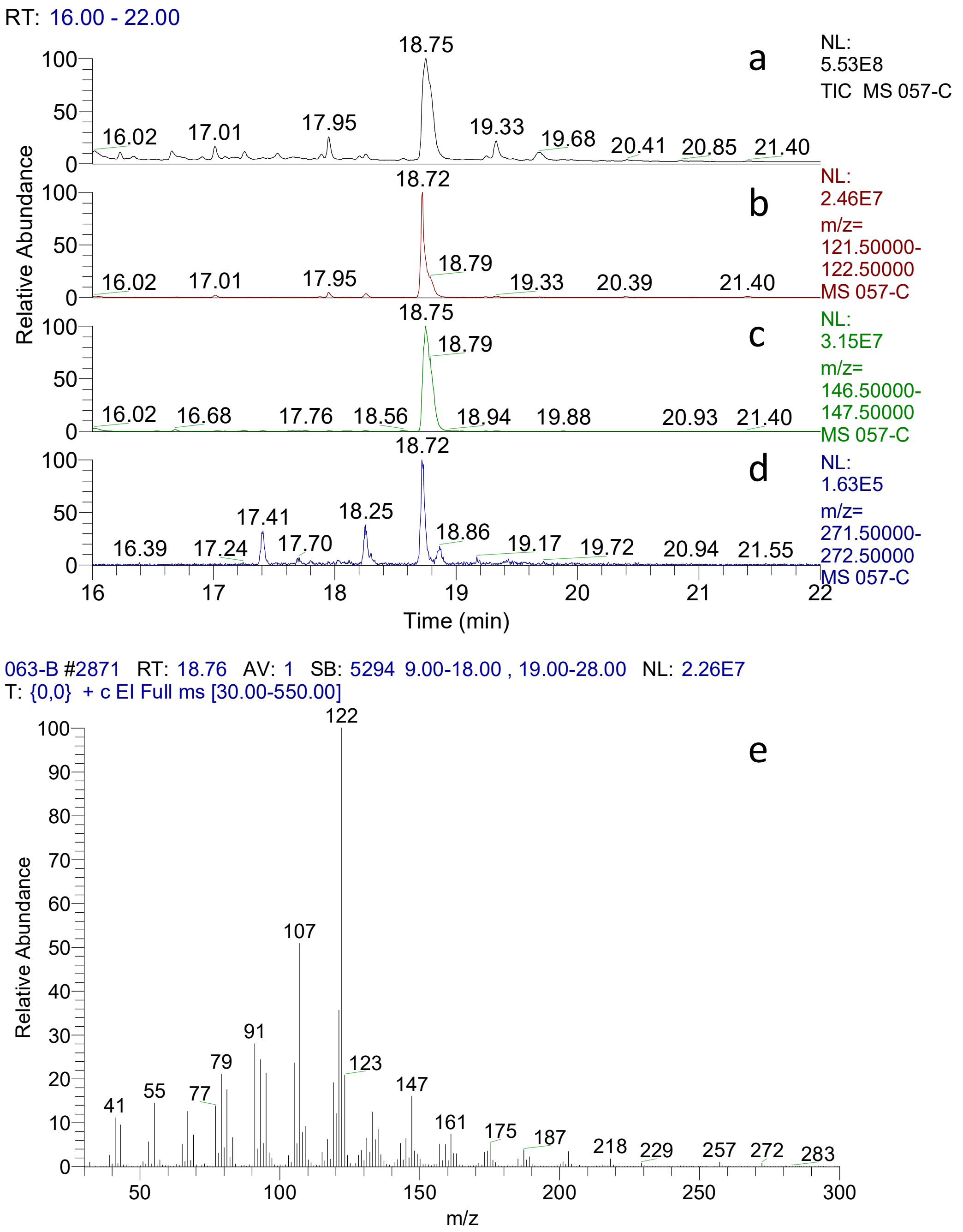

Supplementary Figure 25. **GC-MS analysis of taxadiene in transient co-expression of HMGR and TS in *N. benthamiana* leaves.** (a) TIC of hexane extract of HMGR and TS transient co-expression leaves; (b) Extracted Ion Chromatography for m/z^+ 122 corresponding to taxa-4(5), 11(12)-diene; (c) EIC for m/z^+ 147 representing a putative unknown compound; (d) EIC for m/z^+ 272 representing taxadiene and its analogs; (e) Mass spectrum of the peak at 18.75 min. The retention time and the mass spectrum are very similar with that of authentic taxa-4(5),11(12)-diene. A contaminating compound that was wrapped with taxadiene was identified in the subsequent experiments overexpressing HMGR/tHMGR in NTS1-T2 and wild type tobacco.

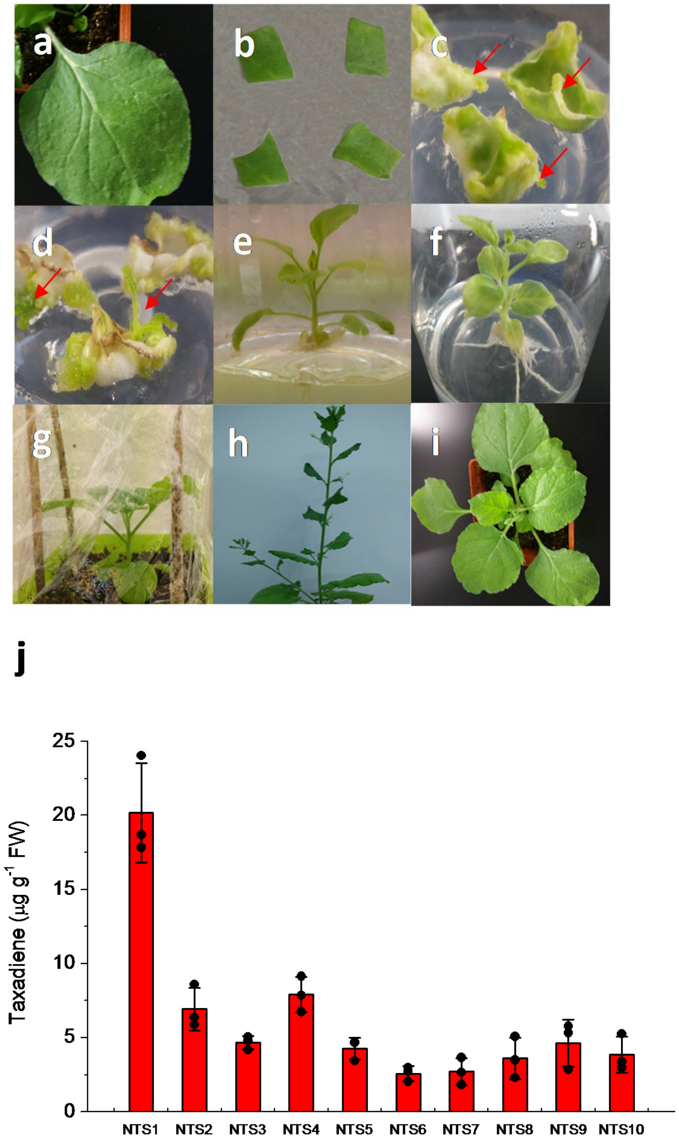

Supplementary Figure 26. *Agrobacterium*-mediated genetic transformation of tobacco leaf explants via direct regeneration. a-i，General protocol of stable transformation of tobacco with LBA4404-pEAQ-TS. (a) Leaf explants from wild type *N. benthamiana*; (b) Leaf explants co-cultivated with LBA4404-pEAQ-TS plated on MS+0.5 mg/L 6BA; (c-d) Microscopic view of direct regeneration (arrow showing regenerating buds); (e) Regenerated plant on MS+0.5 mg/l NAA; (f) Rooted plant on ½MS+0.5 mg/l NAA; (g) Transgenic plant for acclimation; (h) Transgenic plant T1; (i) Transgenic plant T2; (j). The yield of taxa-4(5),11(12)-diene measured from ten stable transgenic TS lines NTS1-NTS10. No taxadiene could be detected in the wild type of *N. benthamiana*. N.D. - not detected. Data in **j** represent the mean of n = 3 biologically independent samples (closed circles) and error bars show standard deviation. Source data of Supplementary Fig. 26j are provided as a Source Data file.

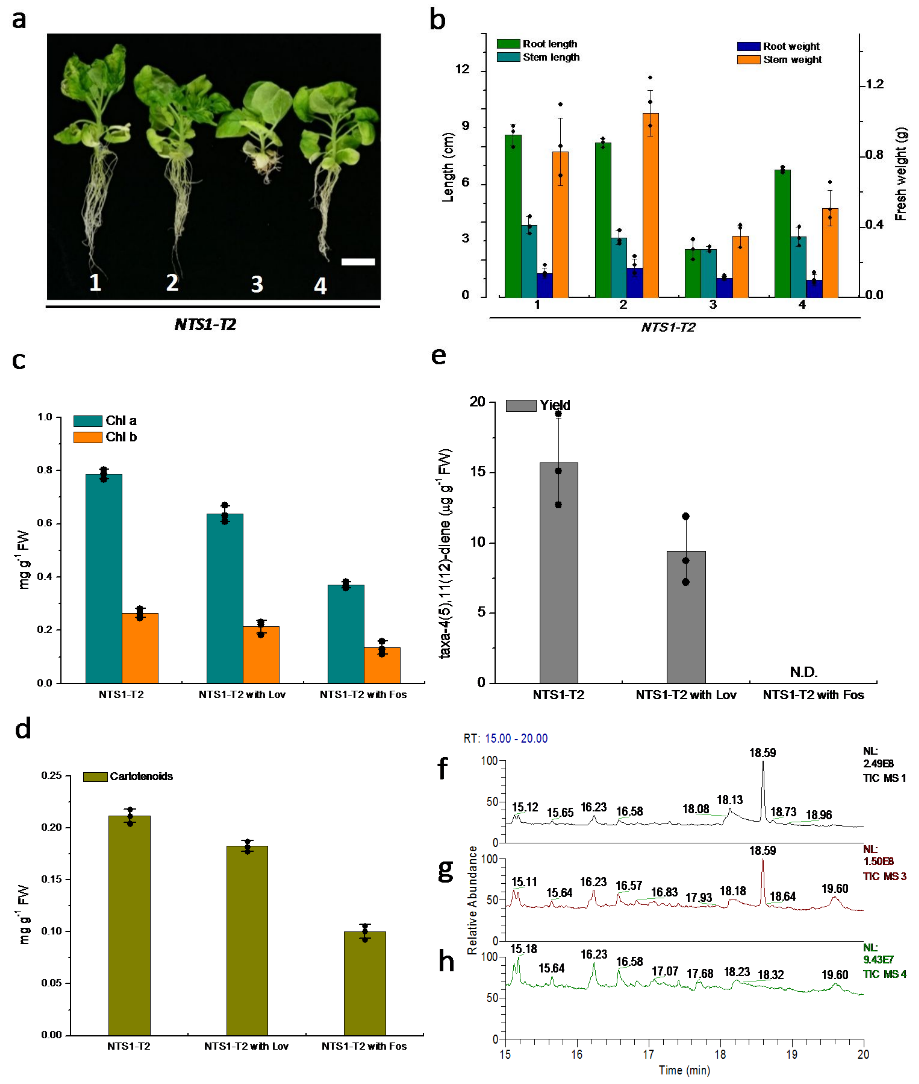

Supplementary Figure 27. Phenotypes of stable transgenic *N. benthamiana* plants expressing TS and effects of MVA and MEP pathway inhibition. (a) Phenotype of transgenic tobacco lines NTS1-T2 after 28 days with/without treatment (10 µM lovastatin or 150 µM fosmidomycin) on ½MS media: (1) plant from germination seedlings; (2) seedling transplanted to 1/2MS; (3) seedling growing on media with lovastatin; (4) seedling growing on media with fosmidomycin. bar = 2.0 cm; (b) Effect of lovastatin and fosmidomycin on root and stem development; (c-d) Effect of lovastatin and fosmidomycin on leaf quantities of chlorophyll a, b, and total carotenoids; (e) Effect of lovastatin and fosmidomycin on production of taxadiene; N.D. indicates not detected. (f - h) GC-MS analysis of taxa-4(5),11(12)-diene in NTS1-T2 line plant: (f) NTS1-T2 without treatment (control); (g) treated with lovastatin; (h) treated with fosmidomycin. The peak (tR =18.59 min) represents the desired product taxa-4(5),11(12)-diene. Chl*a*, Chlorophyll a; Chl*b*, Chlorophyll b. Data in b-e represent the mean of n = 3 biologically independent samples (closed circles) and error bars show standard deviation. Source data of Supplementary Fig. 27b-e are provided as a Source Data file.

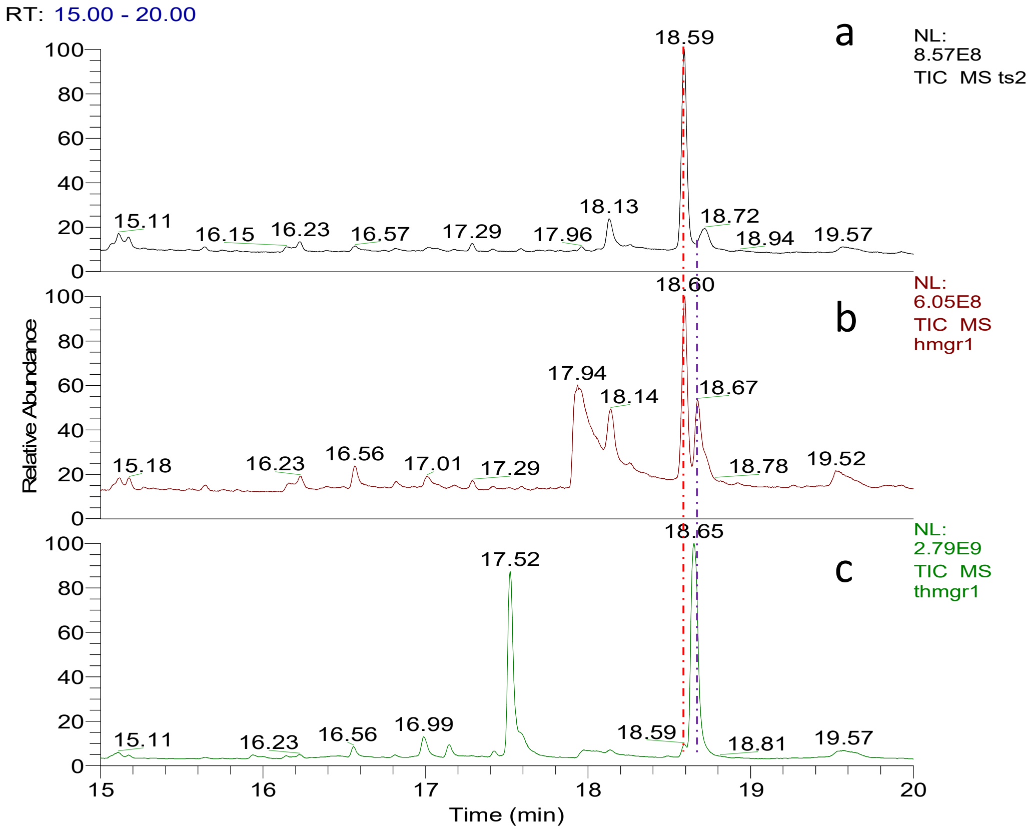

Supplementary Figure 28. GC-MS analysis of taxadiene after transient overexpression of HMGR and tHMGR in TS transgenic tobacco leaves. (A) TIC of transgenic tobacco leaves; (B) TIC of transgenic tobacco leaves with HMGR transient expression; (C) Transgenic tobacco leaves with tHMGR transient expression. The peak at 18.60 min represent taxa-4(5),11(12)-diene (red dashed line), and the peak at 18.65 represent a putative unknown compound (purple dashed line).

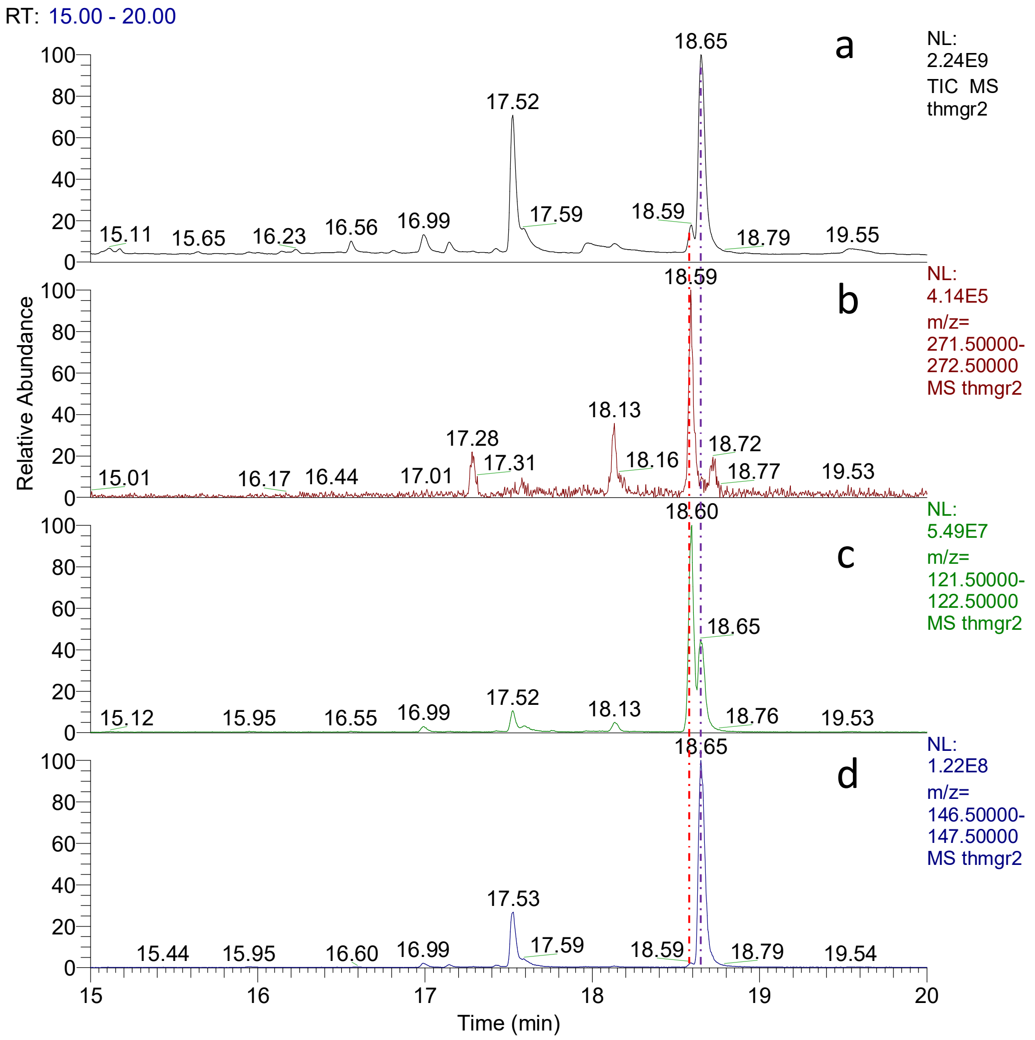

Supplementary Figure 29. GC-MS analysis of taxadiene and the unknown metabolite in transient tHMGR expression TS transgenic tobacco. (a) TIC of extracts from tHMGR expressing NTS1-T2 tobacco; (b) SIM of m/z 272 representing taxadiene; (c) SIM of m/z 122 representing the major fragment of taxadiene; (d) SIM of m/z 147 representing the major fragment of the native contaminating metabolite of tobacco. The peak at 18.60 min represent taxa-4(5),11(12)-diene (red dashed line), and the peak at 18.65 represent a putative unknown compound (purple dashed line).

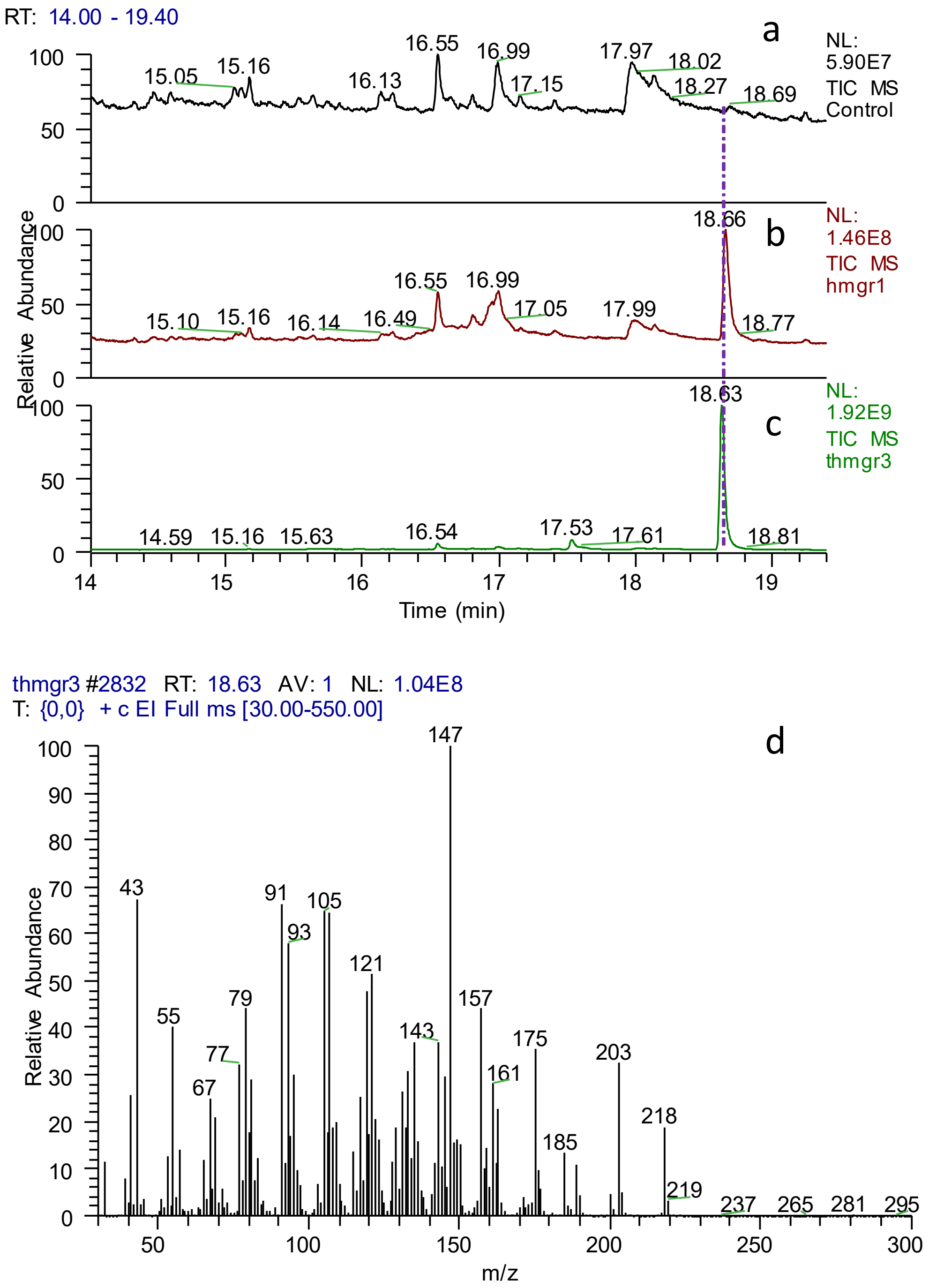

Supplementary Figure 30. GC-MS analysis of metabolites in transient HMGR and tHMGR expression and in wild-type tobacco leaves. (a) TIC of extracts from wild type tobacco; (b) TIC of extracts from HMGR expressing tobacco; (c) TIC of extracts from tHMGR expressing tobacco. A trace amount of the unknown peak that eluted together with taxadiene could be detected in wild-type plants and the yield improved with the expression of HMGR. Overexpressing tHMGR drastically improved the peak; (d) Mass spectrum of the unknown compound.

Supplementary Table 1. Primers for transient plasmid construction with In-fusion.

Genes	Primers	Sequences (5' → 3')*
TS-FLAG	pEAQ-TS-F	*CATCACCATCAT*<u>CCCGGG</u> ATGGCTCAGCTCTCATTTAATG
	pEAQ-TS-FLAG-R	*CCAGAGTTAAAGGC*<u>CTCGAG</u>CTA**CTTATCGTCGTCATCCTTGTAATC** TACTTGAATTGGATCAATATA
T5H-HA	pEAQ-T5H-F	*CATCACCATCAT*<u>CCCGGG</u> ATGGACGCCCTGTATAAGAGC
	pEAQ-T5H-HA-R	*CCAGAGTTAAAGGC*<u>CTCGAG</u>CTA**AGCGTAATCTGGAACATCGTA**TG GGTATGGTCTCGGAAA
CPR-Myc	pEAQ-CPR-F	*CATCACCATCAT*<u>CCCGGG</u> ATGCAGGCTAATTCCAACACGGTGG
	pEAQ-CPR-Myc-R	*CCAGAGTTAAAGGC*<u>CTCGAG</u>TCA**CAGATCCTCTTCAGAGATGAGTT TCTGCTC**CCATATATCTCGTAAGTATCTTCC
TS	pEAQ-TS-F	*CATCACCATCAT*<u>CCCGGG</u> ATGGCTCAGCTCTCATTTAATG
	pEAQ-TS-R	*CCAGAGTTAAAGGC*<u>CTCGAG</u>CTATACTTGAATTGG
T5H	pEAQ-T5H-F	*CATCACCATCAT*<u>CCCGGG</u> ATGGACGCCCTGTATAAGAGC
	pEAQ-T5H-R	*CCAGAGTTAAAGGC*<u>CTCGAG</u>CTATGGGTATGGTCT
CPR	pEAQ-CPR-F	*CATCACCATCAT*<u>CCCGGG</u> ATGCAGGCTAATTCCAACACGGTGG
	pEAQ-CPR-R	*CCAGAGTTAAAGGC*<u>CTCGAG</u>TCACCATATATCTCG
DXS	pEAQ-DXS-F	*CATCACCATCAT*<u>CCCGGG</u> ATGGCAGCAACAATA
	pEAQ-DXS-R	*CCAGAGTTAAAGGC*<u>CTCGAG</u> TTATTGGAGATCAAA
DXR	pEAQ-DXR-F	*CATCACCATCAT*<u>CCCGGG</u> ATGGCTCTGAAAATT
	pEAQ-DXR-R	*CCAGAGTTAAAGGC*<u>CTCGAG</u> CTACTTATCGTCGTC
IspH	pEAQ-IspH-F	*CATCACCATCAT*<u>CCCGGG</u> ATGGCTAAAGCCTGTGCA
	pEAQ-IspH-R	*CCAGAGTTAAAGGC*<u>CTCGAG</u> CTATGCTACTTGCAACGC
HMGR	pEAQ-HMGR-F	*CATCACCATCAT*<u>CCCGGG</u> ATGGAGGTTGTTGGG
	pEAQ-HMGR-R	*CCAGAGTTAAAGGC*<u>CTCGAG</u> TTAGGGTGATAATGC
tHMGR	pEAQ-tHMGR-F	*CATCACCATCACCATCAT*<u>CCCGGG</u>ATGTATGTGCAAAACTTCAT
IDI	pEAQ-IDI-F	*CATCACCATCAT*<u>CCCGGG</u> ATGGGTGAGGCAGCC
	pEAQ-IDI-R	*CCAGAGTTAAAGGC*<u>CTCGAG</u> CTATGTCAAGTTATG
GGPPS	pEAQ-GGPPS-F	*CATCACCATCAT*<u>CCCGGG</u> ATGGCTTACACGGCA
	pEAQ-GGPPS-R	*CCAGAGTTAAAGGC*<u>CTCGAG</u> TCACAGATCCTCTTC

*Restriction enzyme recognition sites Sma I and Xho I are underlined; Homology region is italic; The FLAG tag is marked in red, the HA in purple, and the Myc in green.

Supplementary Table 2. **Chloroplast transit peptide predictions of sequences.**

Protein	GenBank	ChloroP 1.1 Server prediction results[a*]					TargetP 1.1 Server prediction results[b]						TMHMM 2.0 Server prediction results[c]	Plant-PLoc[d]
		Length	Score	cTP	CS score	cTP length	cTP	mTP	SP	other	Loc	RC		
TS	AAC49310	862	0.556	Y	6.735	58	0.762	0.045	0.013	0.149	C	2	No TMhelix	Chloroplast
T5H	AAQ56240	499	0.456	-	1.878	73	0.119	0.024	0.031	0.734	-	2	TMhelix 20-42	Endoplasmic reticulum
CPR	AAT76449	717	0.455	-	0.945	62	0.340	0.088	0.073	0.668	-	4	TMhelix 1st 26-28; 2nd 55-74	Endoplasmic reticulum

*a: Output interpretation of ChloroP results are described on http://www.cbs.dtu.dk/services/ChloroP/;

b: Output interpretation of TargetP results are described on http://www.cbs.dtu.dk/services/TargetP/;

c: Output interpretation of TMHMM results are described on http://www.cbs.dtu.dk/services/TMHMM-2.0/.

d: Output interpretation of Plant-Ploc results are described on http://www.csbio.sjtu.edu.cn/bioinf/plant-multi/

Supplementary Table 3. **Candidate genes of MEP and MVA pathways in *N. benthamiana*.**

Query sequences			Subject sequences	
**Genes**	**Description**	**Genbank ID**	**Genes**	**Genbank sequences**
NtDXS	*Nicotiana tabacum* 1-D-deoxyxylulose 5-phosphate synthase mRNA, complete cds	EU650419	*NbDXS*	gi|641376751|emb|CBMM010010114.1|
NbDXR	*Nicotiana benthamiana* partial mRNA for 1-deoxy-D-xylulose-5-phosphate reductoisomerase	AM236596	*NbDXR*	AM236596
NsIspD	*Nicotiana sylvestris* 2-C-methyl-d-erythritol 2,4-cyclodiphosphate synthase mRNA, complete cds	KC961733	*NbIspD*	gi|641377427|emb|CBMM010009438.1|
NtIspE	*Nicotiana tabacum* 4-diphosphocytidyl-2-C-methyl-D-erythritol kinase (ispE) mRNA, complete cds	KJ159923	*NbIspE*	gi|641368548|emb|CBMM010018317.1|
NlIspF	*Nicotiana langsdorffii* x *Nicotiana sanderae* 2-C-methyl-D-erythritol 2,4-cyclodiphosphate synthase mRNA, partial cds	EF636808	*NbIspF*	gi|641313060|emb|CBMM010073805.1|
NbIspG	*Nicotiana benthamiana* (E)-4-hydroxy-3-methylbut-2-enyl diphosphate synthase mRNA, partial cds	AY497303	*NbIspG*	AY497303
NbIspH	*Nicotiana benthamiana* isopentenyl/dimethylallyl diphosphate synthase mRNA, partial cds	AY497304	*NbIspH*	AY497304
NbHMGS	(*Nicotiana benthamiana* NbHMGS1a mRNA for hydroxymethylglutaryl coenzyme A synthase, complete cds	LC015756	*NbHMGS*	GenBank: LC015756.1
NbHMGR1	*Nicotiana benthamiana* NbHMGR1 mRNA for hydroxy 3 methylglutaryl coenzyme A reductase, complete cds	LC015758	*NbHMGR*	GenBank: LC015758.1
NlMK	*Nicotiana langsdorffii* x *Nicotiana sanderae* mevalonate kinase mRNA, partial cds	EF636814	*NbMK*	gi|641353821|emb|CBMM010033044.1|
NsPK	PREDICTED: *Nicotiana sylvestris* phosphomevalonate kinase-like (LOC104216953), transcript variant X2, mRNA	XM_0097670 95	*NbPK*	gi|641382293|emb|CBMM010004572.1|
NtIDI1	*Nicotiana tabacum* ipi1 mRNA for isopentenyl diphosphate isomerase 1, complete cds	AB049815	*NbIDI*	gi|641385458|emb|CBMM010001407.1|
NaGGPPS	*Nicotiana attenuata* geranylgeranyl pyrophosphate synthase (ggpps) mRNA, complete cds	EF382626	*NbGGPPS*	gi|641348712|emb|CBMM010038153.1|
NbActin	*Nicotiana benthamiana* actin (act) mRNA, act-b allele, partial cds	JQ256516	*NbActin*	GenBank: JQ256516

Supplementary Table 4. **Primers for qRT-PCR analysis.**

Genes	Primers (5’ → 3’)
NbHMGR	F: TTGAGGATGAAAACGATGAG<br>R: ATTCCAAAGAGTATGACGGC
NbMK	F: CACAAAGGTCGGGAGAAATA<br>R: CTGACAGAAGGGTTGGTAAT
NbPK	F: CCACCTTTTACGTCAATCAC<br>R: GAACTAACATCAAACCCGCT
NbDXS	F: CTGCCATTGATGACAGACCA<br>R: AGCAACATGAGACCCGAAAC
NbDXR	F: CAGCTACAACCTTAACCACC<br>R: TTCTCAGCGACTATATCCAA
NbIspD-F	F: TTTGGTGGTTTCTTCTTCTG<br>R: CTTCCATTTAGGGTCAGTGT
NbIspE	F: CTTACTGTACGGGTAGGGGC<br>R: AACTTCAAAGGCAGGAGGTT
NlIspF	F: CCTTCTTCGGTATTCATGGA<br>R: ATGTGCTTCCTGAATGGCTG
NbIspG	F: GTTGTGTCTGTGCGTGGTGA<br>R: CATTGGGAAAGTGTATGTGA
NbIspH	F: GAAGGGAGAAACAGAAGAAA<br>R: GTCAATCCAATATGAGGGAA
NbHMGS	F: AACCGGCCACATTAATACCA<br>R: TCACAAAGTCCTTACCACCA
NbHMGR	F: TTGAGGATGAAAACGATGAG<br>R: ATTCCAAAGAGTATGACGGC
NbMK	F: CACAAAGGTCGGGAGAAATA<br>R: CTGACAGAAGGGTTGGTAAT
NbPK	F: CCACCTTTTACGTCAATCAC<br>R: GAACTAACATCAAACCCGCT
NbIDI	F: GGTGATGTTGAAGCTGATGC<br>R: GACACTCGGTAGAGTGGATG
NbGGPPS	F: TGGGATTGGAAAAGGCTAAG<br>R: CAGGTTGATTCAAACGACAG
NbActin	F: TATTCCCTAGTATTGTTGGC<br>R: CTGGGGTATTAAAAGTCTCA

Supplementary Table 5. Primers for multiple cassettes plasmids construction with Golden gate assembly.

Primers	Sequences (5' → 3')*
pEAQ-GG-1F	CAGGCTTGTCCACATCATCTGTG
pEAQ-GG-1R	CACAGATGATGTGGACAAGCCTG
pEAQ-GG-2F	TAAGAATTCGAGCTCCACCGCGG
pEAQ-GG-2R	CCGCGGTGGAGCTCGAATTCTTA
pEAQ-GG-3F	CTCAAGCTTGGCGCGCCAGCTTG
pEAQ-GG-3R	CAAGCTGGCGCGCCAAGCTTGAG
pEAQ-GG-4F	CCGATGGAAACGTTTAAGCA*GGTCTC*CTATACGTAATCATGGTCATAGCTGTTGC
pEAQ-GG-4R	TGCTTAAACGTTTCCATCGG*GGTCTC*TCTTAATTAACAATTCACTGGCCGTCG
pEAQ-GG-3Gene-AF	*GGTCTC*GTAAGAATTCGAGCTCCACCGC
pEAQ-GG-3Gene-BR	*GGTCTC*CTCGTGCGCGCCAAGCTTGAG
pEAQ-GG-3Gene-BF	*GGTCTC*GACGAAATTCGAGCTCCACCGC
pEAQ-GG-3Gene-CR	*GGTCTC*CTCCAGCGCGCCAAGCTTGAG
pEAQ-GG-3Gene-CF	*GGTCTC*GTGGAAATTCGAGCTCCACCGC
pEAQ-GG-3Gene-DR	*GGTCTC*CCTGAGCGCGCCAAGCTTGAG
pEAQ-GG-3Gene-DF	*GGTCTC*GTCAGAATTCGAGCTCCACCGC
pEAQ-GG-3Gene-ZR	*GGTCTC*CTATAGCGCGCCAAGCTTGAG

*Restriction enzyme recognition site Bsa I is underlined.

Supplementary Table 6. Primers used for fusion protein expression plasmids construction.

Primers	Sequences (5' → 3')*	Usage
pEAQ-TS-F	*CATCACCATCATCCCGGG*ATGGCTCAGCTCTCATTTAATG	For tp(TS)/GFP, TS/GFP, tp(TS)/trT5H/trCPR construction
pEAQ-T5H-F	*CATCACCATCATCCCGGG*ATGGACGCCCTGTATAAGAGC	For T5H/GFP construction
pEAQ-trT5H-F	*CATCACCATCATCCCGGG*ATGCGTTCTAAACGCCA	For trT5H/trCPR construction
tp(TS)-GFP-F	GGTCCTGTCGTAATGATGGTAGATCTGACT	For tp(TS)/GFP construction
tp(TS)-GFP-R	AGTCAGATCTACCATCATTACGACAGGACC	For tp(TS)/GFP construction
TS-GFP-F	GATCCAATTCAAGTAGGAGGAGGAGGAGGAGGAATGGTAGATCTGACT	For TS/GFP construction
TS-GFP-R	AGTCAGATCTACCATTCCTCCTCCTCCTCCTCCTACTTGAATTGGATC	For TS/GFP construction
tp(T5H)-GFP-F	CTTCTCCTGCTCTTCATGGTAGATCTGACT	For tp(T5H)/GFP construction
tp(T5H)-GFP-R	AGTCAGATCTACCATGAAGAGCAGGAGAAG	For tp(T5H)/GFP construction
T5H-GFP-F	CTGTTTCCGAGACCAGGAGGAGGAGGAGGAGGAATGGTAGATCTGACT	For T5H/GFP construction
T5H-GFP-R	AGTCAGATCTACCATTCCTCCTCCTCCTCCTCCTGGTCTCGGAAACAG	For T5H/GFP construction
pEAQ-GFP-R	*CCAGAGTTAAAGGCCTCGAG*TTAGCTAGCTTTGTATA	For TS/GFP, tp(TS)/GFP, T5H/GFP, tp(T5H)/GFP construction
tp(TS)-trT5H-F	GTCCTGGTCCTGTCGTAATGCGTTCTAAACGCCACTCCTC	For tp(TS)/trT5H/trCPR construction
tp(TS)-trT5H-R	GAGGAGTGGCGTTTAGAACGCATTACGACAGGACCAGGAC	For tp(TS)/trT5H/trCPR construction
trT5H-trCPR-F	AAACTGTTTCCGAGACCAGGATCTACTGGATCTAGGAGGGGAGGATCGGAT	For tp(TS)/trT5H/trCPR, trT5H/trCPR construction
trT5H-trCPR-R	ATCCGATCCTCCCCTCCTAGATCCAGTAGATCCTGGTCTCGGAAACAGTTT	For tp(TS)/trT5H/trCPR trT5H/trCPR construction
pEAQ-CPR-R	*CCAGAGTTAAAGGCCTCGAG*TCACCATATATCTCG	For tp(TS)/trT5H/trCPR, trT5H/trCPR construction
CPR-CFP-F	AGAAATTACAGATGGATGGAGGAGGAGGAGGAATGGTGAGCAAGGGCGAGG	For tp(TS)/trT5H/trCPR/CFP construction
CPR-CFP-R	CCTCGCCCTTGCTCACCATTCCTCCTCCTCCTCCATCCATCTGTAATTTCT	For tp(TS)/trT5H/trCPR/CFP construction
pEAQ-CFP-R	*AATGAAACCAGAGTTAAAGGCCTCGAG*TTACTTGTACAGCTCGTCCAT	For tp(TS)/trT5H/trCPR/CFP construction

*Restriction enzyme recognition sites Sma I and Xho I are underlined; Homology region is italic; The linker peptide GGGGG used for chimeras TS/GFP, T5H/GFP construction is marked in red, the linker peptide GSTGS used for chimeras trT5H/trCPR, tp(TS)/trT5H/trCPR construction is marked in green.

Supplementary references

1. Hasan, M. M. et al. Metabolic engineering of *Nicotiana benthamiana* for the increased production of taxadiene. *Plant Cell Rep.* **33**, 895–904 (2014).

2. Wang K (Ed). *Agrobacterium Protocol*, volume (II), 2nd edition. Humana press, Totowa, pp 143–154 (2006).

3. Lichtenthaler, H. K. Chlorophylls and carotenoids: Pigments of photosynthetic biomembranes. *Methods Enzymol.* **148**, 350–382 (1987).

4. Murashige, T. & Skoog, F. A revised medium for rapid growth and bioassays with tobacco. *Physiol. Plant* **15**, 473–497 (1962).

5. Rubenstein, S. M. & Williams, R. M. Studies on the biosynthesis of Taxol: Total synthesis of taxa-4(20),11(12)-diene and taxa-4(5),11(12)-diene. The first committed biosynthetic intermediate. *J. Org. Chem.* **60**, 7215–7223 (1995).

6. Koepp, A. E. *et al.* Cyclization of geranylgeranyl diphosphate to taxa-4 (5), 11 (12) -diene is the committed step of Taxol biosynthesis in *Pacific Yew*. *J. Biol. Chem.* **270**, 8686–8690 (1995).

7. Biggs, B. W. *et al.* Orthogonal assays clarity the oxidative biochemistry of Taxol P450 CYP725A4. *ACS chem biol.* **11**, 1445-1451 (2016).

8. Rotein, D. et al. CYP725A4 from yew catalyzes complex structral rearrangement of taxa-4(5),11(12)-diene into the cyclic ether 5(12)-oxa-3(11)-cyclotacane. *J. Biol. Chem.* **283**, 6067-6075 (2008).
