# Supplementary material for: Chloroplastic metabolic engineering coupled with isoprenoid pool enhancement for committed taxanes biosynthesis in Nicotiana benthamiana
Source: Nat Commun. 2019 Oct 24;10:4850. doi: 10.1038/s41467-019-12879-y (PMC6813417; doi:10.1038/s41467-019-12879-y)
Supplement: Supplementary file 2 — Peer Review [file 41467_2019_12879_MOESM2_ESM.pdf]

Editorial Note: In their review of the first version of this manuscript, Reviewer #1 added their comments to the manuscript file. In their review of the second version of this manuscript, Reviewer #4 added their comments to the manuscript file. These comments, excluding minor textual revisions, have been copied into this Peer Review File.

Reviewers' comments:

Reviewer #1 (Remarks to the Author):

What are the major claims of the paper?
The authors report improved biosynthesis of a taxane precursor taxa-11(12),4(20)-dien-5-ol that is presumed to be a precursor on the pathway to paclitaxel (Taxol) in <i>Taxus</i> plants. This biosynthetic pathway was engineered in a heterologous tobacco host (<i>Nicotiana benthamiana</i>) that enabled the authors to streamline engineering efforts over those presented in earlier independent studies. Their achievement to biosynthesize the second committed intermediate on the Taxol pathway centered on targeting the pathway enzymes to a centralized location: the plastids and using the primary metabolism of the host plant to supply the feedstock of geranyl geranyl diphosphate to the pathway. An intriguing observation was that increasing the expression of primary metabolic enzyme (namely the DXP or MVA) increased the production of the diterpenoid taxa-11(12),4(20)-diene. Diterpenes of this type are thought canonically to derive exclusively (or principally, at least) from the DXP primary metabolic pathway.

These efforts improved metabolic flux to the taxadiene intermediate over the amounts measured in earlier engineering efforts where leader sequences that targeted enzymes to their natural location in a heterologous host were retained. The authors realized that placing the enzymatic machinery in separate organelles of the host prevented metabolic flux through the pathway.

Are they novel and will they be of interest to others in the community and the wider field?
Yes, not only does this research showcase the application of the robust plant Nicotiana benthamiana, it also demonstrates that engineering a complex metabolic pathway into a new host requires benefits from knowing the limitations of metabolite transport in the chassis organism.

If the conclusions are not original, it would be helpful if you could provide relevant references.
The conclusions are not original, and the authors do cite some of the prior art in their references, yet have a glaring omission of the seminal work by Croteau et al., who identified and characterized the genes used later in iterations of Taxol pathway engineering efforts as follows:

Guerra-Bubb, J., Croteau, R., and Williams, R. M. (2012) The early stages of taxol biosynthesis: an interim report on the synthesis and identification of early pathway metabolites, Nat. Prod. Rep. 29, 683-696.

Kaspera, R., and Croteau, R. (2006) Cytochrome P450 oxygenases of Taxol biosynthesis, Phytochem. Rev. 5, 433-444.

Croteau, R., Ketchum, R. E., Long, R. M., Kaspera, R., and Wildung, M. R. (2006) Taxol biosynthesis and molecular genetics, Phytochem. Rev. 5, 75-97.

Dejong, J. M., Liu, Y., Bollon, A. P., Long, R. M., Jennewein, S., Williams, D., and Croteau, R. B. (2006) Genetic engineering of taxol biosynthetic genes in Saccharomyces cerevisiae, Biotechnol. Bioeng. 93, 212-224.

Jennewein, S., Park, H., DeJong, J. M., Long, R. M., Bollon, A. P., and Croteau, R. B. (2005) Coexpression in yeast of Taxus cytochrome P450 reductase with cytochrome P450 oxygenases involved in Taxol biosynthesis, Biotechnol. Bioeng. 89, 588-598.

Chau, M., and Croteau, R. (2004) Molecular cloning and characterization of a cytochrome P450 taxoid 2alpha-hydroxylase involved in Taxol biosynthesis, Arch. Biochem. Biophys. 427, 48-57.

Jennewein, S., Long, R. M., Williams, R. M., and Croteau, R. (2004) Cytochrome p450 taxadiene 5alpha-hydroxylase, a mechanistically unusual monooxygenase catalyzing the first oxygenation step of taxol biosynthesis, Chem Biol 11, 379-387.

Horiguchi, T., Rithner, C. D., Croteau, R., and Williams, R. M. (2002) Studies on taxol biosynthesis. Preparation of taxa-4(20),11(12)-dien-5 alpha-acetoxy-10 beta-ol by deoxygenation of a taxadiene tetraacetate obtained from Japanese yew, J Org Chem 67, 4901-4903.

Jennewein, S., Rithner, C. D., Williams, R. M., and Croteau, R. B. (2001) Taxol biosynthesis: taxane 13 alpha-hydroxylase is a cytochrome P450-dependent monooxygenase, Proc. Natl. Acad. Sci. U. S. A. 98, 13595-13600.

Walker, K., and Croteau, R. (2001) Taxol biosynthetic genes, Phytochemistry 58, 1-7.

Wheeler, A. L., Long, R. M., Ketchum, R. E., Rithner, C. D., Williams, R. M., and Croteau, R. (2001) Taxol biosynthesis: differential transformations of taxadien-5 alpha-ol and its acetate ester by cytochrome P450 hydroxylases from Taxus suspension cells, Arch. Biochem. Biophys. 390, 265-278.

Walker, K., Ketchum, R. E., Hezari, M., Gatfield, D., Goleniowski, M., Barthol, A., and Croteau, R. (1999) Partial purification and characterization of acetyl coenzyme A: taxa-4(20),11(12)-dien-5alpha-ol O-acetyl transferase that catalyzes the first acylation step of taxol biosynthesis, Arch. Biochem. Biophys. 364, 273-279.

Hefner, J., Ketchum, R. E., and Croteau, R. (1998) Cloning and functional expression of a cDNA encoding geranylgeranyl diphosphate synthase from Taxus canadensis and assessment of the role of this prenyltransferase in cells induced for taxol production, Arch. Biochem. Biophys. 360, 62-74.

Huang, K. X., Huang, Q. L., Wildung, M. R., Croteau, R., and Scott, A. I. (1998) Overproduction, in Escherichia coli, of soluble taxadiene synthase, a key enzyme in the Taxol biosynthetic pathway, Protein Expr. Purif. 13, 90-96.

Hefner, J., Rubenstein, S. M., Ketchum, R. E., Gibson, D. M., Williams, R. M., and Croteau, R. (1996) Cytochrome P450-catalyzed hydroxylation of taxa-4(5),11(12)-diene to taxa-4(20),11(12)-dien-5alpha-ol: the first oxygenation step in taxol biosynthesis, Chem Biol 3, 479-489.

Wildung, M. R., and Croteau, R. (1996) A cDNA clone for taxadiene synthase, the diterpene cyclase that catalyzes the committed step of taxol biosynthesis, J. Biol. Chem. 271, 9201-9204.

Lin, X., Hezari, M., Koepp, A. E., Floss, H. G., and Croteau, R. (1996) Mechanism of taxadiene synthase, a diterpene cyclase that catalyzes the first step of taxol biosynthesis in Pacific yew, Biochemistry 35, 2968-2977.

Koepp, A. E., Hezari, M., Zajicek, J., Vogel, B. S., LaFever, R. E., Lewis, N. G., and Croteau, R. (1995) Cyclization of geranylgeranyl diphosphate to taxa-4(5),11(12)-diene is the committed step of taxol biosynthesis in Pacific yew, J. Biol. Chem. 270, 8686-8690.

=========================

This reviewer was disappointed to read that efforts to engineer the paclitaxel pathway into a heterologous host have not advancesd beyond the first 3 intermediates presumed to be committed to the Taxol pathway as shown earlier in the extensive effort by Stephanopolous with the same distribution of off-pathway by-products formed. (Ajikumar, P. K., Xiao, W.-H., Tyo, K. E. J., Wang, Y., Simeon, F., Leonard, E., Mucha, O., Phon, T. H., Pfeifer, B., and Stephanopoulos, G. (2010) Isoprenoid pathway optimization for Taxol precursor overproduction in Escherichia coli, Science 330,

70-74) and by Anterola (Anterola, A., Shanle, E., Perroud, P. F., and Quatrano, R. (2009) Production of taxa-4(5),11(12)-diene by transgenic Physcomitrella patens, Transgenic Res. 18, 655-660), as examples.

Despite the author's excellent extraction of cDNA from <i>T. chinesis</i> needles, without the seminal sequence information deposited by Croteau into the DNA databanks, the characterization of their cDNA sequences would be lengthy. Along these lines, it was unclear why the authors chose to isolate RNA from the needles of the Taxus plant rather than using the abundant extant sequence information of various orthologs to synthesize the needed cDNAs.

As I began reading the manuscript, I was felt the authors would address one of the biggest "impediments" confronted when attempting to engineer the taxol pathway into a chassis organism. The authors state:

" the biggest impediment to achieving total biotechnological production of taxol lies in the dearth of knowledge on its complex biosynthetic pathway, which consist of at least 19 steps from GGPP..."

This reviewer's sense of the Taxol pathway is that after the first modications of the taxadiene core early in the pathway, the order of the pathway transformations is overwhelming to predict a linear route. It is not clear how the current work described by Wang et al. addresses this impediment.

Is the work convincing, and if not, what further evidence would be required to strengthen the conclusions?
The crosstalk of the MVA and MEP pathways is compelling; however, it would be nice if the authors could do 13C-labeling studies to confirm their conclusion that both primary metabolic pathways indeed feed into the taxane diterpenes. In situ immunoblotting to identify the localization of the enzymes in planta would be beneficial. While the peptide target sequences putatively inform on the destination of the expressed enzymes, there is no guarantee that they are transported exclusively to the target organelle. If they (GGPP synthase, TS, T5OH) are distributed amongst organelles and the cytosol where the MVA and MEP pathway are functioning, then one may observe the unusual "crosstalk" proposed by the authors. Described in its current state, the conclusion is only speculative. The latter could be studied by in situ immunoblotting.

On a more subjective note, do you feel that the paper will influence thinking in the field?
This reviewer feels that the paper uses the taxol pathway genes as a model to demonstrate the utility of <i>Nicotiana benthamiana</i> as a chassis host, and this helps others in the area of plant host engineering. Their understanding of organellar targeting in planta is another major consideration that informs other researchers in the biotechnological area. To contrast, this manuscript unfortunately does not advance the area of Taxol biosynthesis as suggested throughout the document.

Reviewer #2 (Remarks to the Author):

The authors Jianhua Li et al. in the manuscript titled “Chloroplastic metabolic engineering coupled with isoprenoid pool enhancement for committed taxol intermediates biosynthesis in Nicotania benthamania” employ an innovative method for metabolic engineering that overcomes the boundaries of metabolome compartmentalization. Here, they elegantly demonstrate what they quipped to be a ‘rudimentary’ notion into a well-principled experiment that supported initial hypotheses with unique

bioinformatics and modular engineering strategies, all of which were validated by metabolite identification and cell imaging.
The authors provide a plant systems for terpenoid production as compared to microbial systems as plants have both the 2-C-methyl-D-erythritol 4-phosphate (MEP) and the mevalonic acid (MVA) pathways to generate the desired terpenoids, thus extensive engineering of introduced pathways can be avoided. Most reports so far have focused on taxadiene synthesis. The authors have used a clever approach by compartmentalizing TS, T5αH, and cytochrome P450 reductase (CPR) in the chloroplastid combined with increasing isoprenoid precursor pool size. They were able to increase production of taxadiene, and also produced taxadiene-5α-ol in a heterologous plant. However, the author has not provided the real ratio of the OCT: taxadiene 5-ol. Also, the GC-MS method used for the identification of the product is not full proof and the authors need to produce more product using this plant source and at least provide NMR for identification of the products as was done in the ACS chemical biology paper by Ajikumar's group. While the engineering in this paper is very novel, the full characterization of the products in required and quantification of the ratio of OCT: 5-alpha ol is important. Another thing the authors can try is to use the reductase of the nicotiana plant which may further increase the production.
This paper is very important and their strategy could be further used to make the other precursors of the taxol synthesis. This is also the first report of the heterologous production of taxadiene-5α-ol in plant cells.
Minor comments: while the manuscript is very well written, the figure quality needs to improved. The GC-MS data is very noisy. Moreover, the authors should include better characterization of the products.
(Lines 144-147):"TS detectable at 2 dpi and accumulated to its highest level at 4 dpi followed by a slow decrease afterwards. CPR showed a similar accumulation profile to that of TS. On the other hand, T5αH was detectable at 2 dpi and continuously increased through 5 dpi."
This was the only point in the manuscript that the authors stated anything about temporal relationships with gene/protein expression, aside from their arguments about limiting carbon flow. Though the authors examined transcriptional levels of MEP and MVA pathway genes (Figure 5a), expression levels as measured by qRT-PCR were only included up to Day 3, leaving this earlier comment in the Results section unaddressed. The authors should revise Figure 5a by including expression values throughout days 1-5 -post transformation.
It would also be very interesting if the authors to include nuclear run-on transcription assays to measure RNA accumulation for each compartmentalized gene of interest (supplemental). The authors do a fantastic job emphasizing the difficulties in navigating the spatial regulation within plant metabolomics capable of complicating engineering efforts. Attention should also be made to the temporal regulation of this metabolic flux, where discussion on participatory gene expression trends could be added. This commentary would greatly improve the present manuscript by suggesting ways for optimizing future metabolic engineering efforts when also considering the time scales of carbon flux, giving the reader improved perspective on the complexity behind the regulation of plant secondary metabolism.

Reviewer #3 (Remarks to the Author):

The manuscript of Li et al. builds upon a very extensive area of investigation focused on building production platforms for fine and specialty chemicals in microbes and plants. The current work is directed to diterpene production in plants and plays upon an equally rich background of directing biosynthesis to distinct cellular compartments such as the chloroplast and the cytoplasmic face of the ER. While the current report does provide some intriguing observations, the work does not standup to the expectations of a break-through report in a Nature specialty journal and the results per se are somewhat preliminary and not rigorously vetted. And while the authors have cited some of the key

publications in this arena, they have not captured the conceptual framework correctly nor accurately.

Over the last 12 years, there have been some outstanding reports on how to direct terpene metabolism in the cytosol and chloroplast compartments. I won't dwell on these but rather use a particular result presented in the current manuscript to illustrate a lack of critical consideration by the authors. Fig. 5b presents results from transiently expressing the chloroplast-targeted TS, plus and minus over-expression of early steps in the MEP and MVA pathways. TS expression by itself yields 5 µg/g FW, while TS+DXS or TS+HMGR yields 45 µg/g. Firstly, what the authors don't examine is comparison between full-length HMGR versus truncated forms. Why is this important? Well, there are many precedent studies showing that expression of the full-length (and hence ER targeted form) does not improve carbon flux while truncated forms do. Second, how do the authors account for a plastid-targeted TS's ability to gain sufficient carbon from a cytoplasmic targeted HMGR? The authors are apt to claim there is exchange between the cytosolic MVA pathway and the plastid MEP pathways. Okay, then the control, over-expression of just HMGR should enhance carotenoid levels. The converse experiment with the DXS over-expression needs an equal intensive assessment. If there is movement of MEP pathway intermediates to the cytosolic pathway, then one would not expect total sterol levels to be impacted, but sterol biosynthetic intermediates. Again, the control experiments are missing here and there isn't sufficient critical assessment of the results themselves.

A second interpretation of the Fig. 5b experiment is that there is inefficient targeting of the TS to the plastid compartment. That is, there are two populations of TS – one directed to the plastid and a second that doesn't make it to the plastid compartment. There are ways to test for this via approaches published over the years. Is this important to distinguish? Yes indeed! Because this could also be helpful in explaining some the P450 expression results. These are details that could have very important ramifications for how the data might be more thoroughly explained.

Still staying with Fig. 5b, coupled expression of the TS with GGPS gives a significant boost to production, and in combination with both HMGR or DXS gives another doubling. However, either TS-HMGR nor TS-DXS gives an equally enhanced production level as the TS+GGPS+HMGR+DXS. So, does this mean that the prenyltransferase isn't significant? I appreciate that the authors provide some mRNA level determinations, but I question the significance/importance of this. Wouldn't actual measurement of enzyme activities in the infiltrated tissue samples be more appropriate? I suspect that the level of enhanced GGPS enzyme activity is very variable. But, if its activity were significant, then elevated levels of GGOH in the tissue samples should be detectable. Were the authors able to detect GGOH?

Another figure that opens more questions than answers is Fig. 6. These panels are providing evidence that targeting of the P450 T5alphaH to the chloroplast can significantly improve the accumulation of oxidized taxanes. Some evidence that the T5alphaH targeted to the chloroplast is actually catalytically competent and active is needed. Targeting a normally cytoplasmic P450 to the chloroplast would entail the vectorial movment of the peptide across the inner membrane, followed by its proper folding and insertion of a heme prosthetic unit. This is possible, but some evidence for such would make for a more compelling story. Equally difficult to understand is how the histobars in 6c actually correlate with GC trace in 6d. The peak for compound 3 looks to be much more significant than that for peak 2. Yet, the quantitation in the histogram is just of the inverse of the chromatogram – peak 2 is twice that of peak 3.

I've focused on a couple panels of data because I wanted to illustrate the depth of understanding I would expect in a paper suitable for a Nature specialty journal.

But there other concerns I have about the manuscript as well.

The error bars presented for all the data are incredibly tight. There are lots of reports of agroinfiltration to illustrate the variability in such data. If one were to standardize exactly what leaf

was infiltrated by position on the plant and time upon when the leaf reaches maturity, and if one were able to demonstrate that they were able to infiltrate exactly the same number of CFUs, and if one were to examine just the infiltration zone only, then perhaps it is possible to obtain data with essentially less than 20% variations. But reading the methods, there isn't sufficient detail to suggest such attention to the details. Nor is there any mention of what the replicates are in each case. I'm assuming there are technical repeats within a single experiment. In fact, there should be documentation comparing between biological repeats between independent experiments.

The authors cite references which they state provide insight into the importance of compartmentalization for diterpene accumulation in N. benthamiana. When I examine those citations, at least one of the articles did not address diterpene production as suggested by the authors. If the authors cite papers it means they have rigorously read those papers and are citing them accurately. This is critical to the integrity of the scientific community.

The authors are also remiss in not mentioning some of the work on transient expression in N. benthamiana for oxidized triterpenes. Wouldn't that work be the precedent for the current work?

In summary, this manuscript represents an important approach to developing production platforms in plants, but the work is preliminary and better suited for a traditionally based specialty journal.

Reviewers' comments:

Reviewer #1 (Remarks to the Author):

What are the major claims of the paper?
The authors report improved biosynthesis of a taxane precursor taxa-11(12),4(20)-dien-5-ol that is presumed to be a precursor on the pathway to paclitaxel (Taxol) in *Taxus* plants. This biosynthetic pathway was engineered in a heterologous tobacco host (*Nicotiana benthamiana*) that enabled the authors to streamline engineering efforts over those presented in earlier independent studies. Their achievement to biosynthesize the second committed intermediate on the Taxol pathway centered on targeting the pathway enzymes to a centralized location: the plastids and using the primary metabolism of the host plant to supply the feedstock of geranylgeranyl diphosphate to the pathway. An intriguing observation was that increasing the expression of primary metabolic enzyme (namely the DXP or MVA) increased the production of the diterpenoid taxa-11(12),4(20)-diene. Diterpenes of this type are thought canonically to derive exclusively (or principally, at least) from the DXP primary metabolic pathway.

These efforts improved metabolic flux to the taxadiene intermediate over the amounts measured in earlier engineering efforts where leader sequences that targeted enzymes to their natural location in a heterologous host were retained. The authors realized that placing the enzymatic machinery in separate organelles of the host prevented metabolic flux through the pathway.

Are they novel and will they be of interest to others in the community and the wider field?
Yes, not only does this research showcase the application of the robust plant Nicotiana benthamiana, it also demonstrates that engineering a complex metabolic pathway into a new host requires benefits from knowing the limitations of metabolite transport in the chassis organism.

If the conclusions are not original, it would be helpful if you could provide relevant references.
The conclusions are not original, and the authors do cite some of the prior art in their references, yet have a glaring omission of the seminal work by Croteau et al., who identified and characterized the genes used later in iterations of Taxol pathway engineering efforts as follows:

Guerra-Bubb, J., Croteau, R., and Williams, R. M. (2012) The early stages of taxol biosynthesis: an interim report on the synthesis and identification of early pathway metabolites, Nat. Prod. Rep. 29, 683-696.

Kaspera, R., and Croteau, R. (2006) Cytochrome P450 oxygenases of Taxol biosynthesis, Phytochem.Rev. 5, 433-444.

Croteau, R., Ketchum, R. E., Long, R. M., Kaspera, R., and Wildung, M. R. (2006) Taxol biosynthesis and molecular genetics, Phytochem.Rev. 5, 75-97.

Dejong, J. M., Liu, Y., Bollon, A. P., Long, R. M., Jennewein, S., Williams, D., and Croteau, R. B. (2006) Genetic engineering of taxol biosynthetic genes in Saccharomyces cerevisiae, Biotechnol. Bioeng. 93, 212-224.

Jennewein, S., Park, H., DeJong, J. M., Long, R. M., Bollon, A. P., and Croteau, R. B. (2005)

Coexpression in yeast of Taxus cytochrome P450 reductase with cytochrome P450 oxygenases involved in Taxol biosynthesis, Biotechnol. Bioeng. 89, 588-598.

Chau, M., and Croteau, R. (2004) Molecular cloning and characterization of a cytochrome P450 taxoid 2alpha-hydroxylase involved in Taxol biosynthesis, Arch. Biochem. Biophys. 427, 48-57.

Jennewein, S., Long, R. M., Williams, R. M., and Croteau, R. (2004) Cytochrome p450 taxadiene 5alpha-hydroxylase, a mechanistically unusual monooxygenasecatalyzing the first oxygenation step of taxol biosynthesis, ChemBiol 11, 379-387.

Horiguchi, T., Rithner, C. D., Croteau, R., and Williams, R. M. (2002) Studies on taxol biosynthesis. Preparation of taxa-4(20),11(12)-dien-5 alpha-acetoxy-10 beta-ol by deoxygenation of a taxadiene tetraacetate obtained from Japanese yew, J Org Chem 67, 4901-4903.

Jennewein, S., Rithner, C. D., Williams, R. M., and Croteau, R. B. (2001) Taxol biosynthesis: taxane 13 alpha-hydroxylase is a cytochrome P450-dependent monooxygenase, Proc. Natl. Acad. Sci. U. S. A. 98, 13595-13600.

Walker, K., and Croteau, R. (2001) Taxol biosynthetic genes, Phytochemistry 58, 1-7.

Wheeler, A. L., Long, R. M., Ketchum, R. E., Rithner, C. D., Williams, R. M., and Croteau, R. (2001) Taxol biosynthesis: differential transformations of taxadien-5 alpha-ol and its acetate ester by cytochrome P450 hydroxylases from Taxus suspension cells, Arch. Biochem. Biophys. 390, 265-278.

Walker, K., Ketchum, R. E., Hezari, M., Gatfield, D., Goleniowski, M., Barthol, A., and Croteau, R. (1999) Partial purification and characterization of acetyl coenzyme A: taxa-4(20),11(12)-dien-5alpha-ol O-acetyl transferase that catalyzes the first acylation step of taxol biosynthesis, Arch. Biochem. Biophys. 364, 273-279.

Hefner, J., Ketchum, R. E., and Croteau, R. (1998) Cloning and functional expression of a cDNA encoding geranylgeranyl diphosphate synthase from Taxus canadensis and assessment of the role of this prenyltransferase in cells induced for taxol production, Arch. Biochem. Biophys. 360, 62-74.

Huang, K. X., Huang, Q. L., Wildung, M. R., Croteau, R., and Scott, A. I. (1998) Overproduction, in Escherichia coli, of soluble taxadiene synthase, a key enzyme in the Taxol biosynthetic pathway, Protein Expr. Purif. 13, 90-96.

Hefner, J., Rubenstein, S. M., Ketchum, R. E., Gibson, D. M., Williams, R. M., and Croteau, R. (1996) Cytochrome P450-catalyzed hydroxylation of taxa-4(5),11(12)-diene to taxa-4(20),11(12)-dien-5alpha-ol: the first oxygenation step in taxol biosynthesis, ChemBiol 3, 479-489.

Wildung, M. R., and Croteau, R. (1996) AcDNA clone for taxadiene synthase, the diterpenecyclase that catalyzes the committed step of taxol biosynthesis, J. Biol. Chem. 271, 9201-9204.

Lin, X., Hezari, M., Koepp, A. E., Floss, H. G., and Croteau, R. (1996) Mechanism of taxadiene synthase, a diterpenecyclase that catalyzes the first step of taxol biosynthesis in Pacific yew,

Biochemistry 35, 2968-2977.

Koepp, A. E., Hezari, M., Zajicek, J., Vogel, B. S., LaFever, R. E., Lewis, N. G., and Croteau, R. (1995) Cyclization of geranylgeranyl diphosphate to taxa-4(5),11(12)-diene is the committed step of taxol biosynthesis in Pacific yew, J. Biol. Chem. 270, 8686-8690.

========================

This reviewer was disappointed to read that efforts to engineer the paclitaxel pathway into a heterologous host have not advancesd beyond the first 3 intermediates presumed to be committed to the Taxol pathway as shown earlier in the extensive effort by Stephanopolous with the same distribution of off-pathway by-products formed. (Ajikumar, P. K., Xiao, W.-H., Tyo, K. E. J., Wang, Y., Simeon, F., Leonard, E., Mucha, O., Phon, T. H., Pfeifer, B., and Stephanopoulos, G. (2010) Isoprenoid pathway optimization for Taxol precursor overproduction in Escherichia coli, Science 330, 70-74) and by Anterola (Anterola, A., Shanle, E., Perroud, P. F., and Quatrano, R. (2009) Production of taxa-4(5),11(12)-diene by transgenic Physcomitrella patens, Transgenic Res. 18, 655-660), as examples.

Despite the author's excellent extraction of cDNA from *T. chinesis* needles, without the seminal sequence information deposited by Croteau into the DNA databanks, the characterization of their cDNA sequences would be lengthy. Along these lines, it was unclear why the authors chose to isolate RNA from the needles of the Taxus plant rather than using the abundant extant sequence information of various orthologs to synthesize the needed cDNAs.

As I began reading the manuscript, I was felt the authors would address one of the biggest "impediments" confronted when attempting to engineer the taxol pathway into a chassis organism. The authors state:

" the biggest impediment to achieving total biotechnological production of taxol lies in the dearth of knowledge on its complex biosynthetic pathway, which consist of at least 19 steps from GGPP..."

This reviewer's sense of the Taxol pathway is that after the first modications of the taxadiene core early in the pathway, the order of the pathway transformations is overwhelming to predict a linear route. It is not clear how the current work described by Wang et al. addresses this impediment.

Is the work convincing, and if not, what further evidence would be required to strengthen the conclusions?
The crosstalk of the MVA and MEP pathways is compelling; however, it would be nice if the authors could do 13C-labeling studies to confirm their conclusion that both primary metabolic pathways indeed feed into the taxanediterpenes. In situ immunoblotting to identify the localization of the enzymes in planta would be beneficial. While the peptide target sequences putatively inform on the destination of the expressed enzymes, there is no guarantee that they are transported exclusively to the target organelle. If they (GGPP synthase, TS, T5OH) are distributed amongst organelles and the cytosol where the MVA and MEP pathway are functioning, then one may observe the unusual "crosstalk" proposed by the authors. Described in its current state, the conclusion is only speculative. The latter could be studied by in situ

immunoblotting.

On a more subjective note, do you feel that the paper will influence thinking in the field?
This reviewer feels that the paper uses the taxol pathway genes as a model to demonstrate the utility of *Nicotiana benthamiana* as a chassis host, and this helps others in the area of plant host engineering. Their understanding of organellar targeting in planta is another major consideration that informs other researchers in the biotechnological area. To contrast, this manuscript unfortunately does not advance the area of Taxol biosynthesis as suggested throughout the document.

Reviewer #2 (Remarks to the Author):

The authors Jianhua Li et al. in the manuscript titled "Chloroplastic metabolic engineering coupled with isoprenoid pool enhancement for committed taxol intermediates biosynthesis in Nicotaniabenthamania" employ an innovative method for metabolic engineering that overcomes the boundaries of metabolome compartmentalization. Here, they elegantly demonstrate what they quipped to be a 'rudimentary' notion into a well-principled experiment that supported initial hypotheses with unique bioinformatics and modular engineering strategies, all of which were validated by metabolite identification and cell imaging.
The authors provide a plant systems for terpenoid production as compared to microbial systems as plants have both the 2-C-methyl-D-erythritol 4-phosphate (MEP) and the mevalonic acid (MVA) pathways to generate the desired terpenoids, thus extensive engineering of introduced pathways can be avoided. Most reports so far have focused on taxadiene synthesis. The authors have used a clever approach by compartmentalizing TS, T5αH, and cytochrome P450 reductase (CPR) in the chloroplastid combined with increasing isoprenoid precursor pool size. They were able to increase production of taxadiene, and also produced taxadiene-5α-ol in a heterologous plant. However, the author has not provided the real ratio of the OCT: taxadiene 5-ol. Also, the GC-MS method used for the identification of the product is not full proof and the authors need to produce more product using this plant source and at least provide NMR for identification of the products as was done in the ACS chemical biology paper by Ajikumar's group. While the engineering in this paper is very novel, the full characterization of the products in required and quantification of the ratio of OCT: 5-alpha ol is important. Another thing the authors can try is to use the reductase of the nicotianaplant which may further increase the production.
This paper is very important and their strategy could be further used to make the other precursors of the taxol synthesis. This is also the first report of the heterologous production of taxadiene-5α-ol in plant cells.
Minor comments: while the manuscript is very well written, the figure quality needs to improved. The GC-MS data is very noisy. Moreover, the authors should include better characterization of the products.
(Lines 144-147):"TS detectable at 2 dpi and accumulated to its highest level at 4 dpi followed by a slow decrease afterwards. CPR showed a similar accumulation profile to that of TS. On the other hand, T5αH was detectable at 2 dpi and continuously increased through 5 dpi."
This was the only point in the manuscript that the authors stated anything about temporal relationships with gene/protein expression, aside from their arguments about limiting carbon flow. Though the authors examined transcriptional levels of MEP and MVA pathway genes (Figure 5a), expression levels as measured by qRT-PCR were only included up to Day 3, leaving this earlier comment in the Results section unaddressed. The authors should revise Figure 5a by including expression values throughout days 1-5 -post transformation.

It would also be very interesting if the authors to include nuclear run-on transcription assays to measure RNA accumulation for each compartmentalized gene of interest (supplemental). The authors do a fantastic job emphasizing the difficulties in navigating the spatial regulation within plant metabolomics capable of complicating engineering efforts. Attention should also be made to the temporal regulation of this metabolic flux, where discussion on participatory gene expression trends could be added. This commentary would greatly improve the present manuscript by suggesting ways for optimizing future metabolic engineering efforts when also considering the time scales of carbon flux, giving the reader improved perspective on the complexity behind the regulation of plant secondary metabolism.

Reviewer #3 (Remarks to the Author):

The manuscript of Li et al. builds upon a very extensive area of investigation focused on building production platforms for fine and specialty chemicals in microbes and plants. The current work is directed to diterpene production in plants and plays upon an equally rich background of directing biosynthesis to distinct cellular compartments such as the chloroplast and the cytoplasmic face of the ER. While the current report does provide some intriguing observations, the work does not standup to the expectations of a break-through report in a Nature specialty journal and the results per se are somewhat preliminary and not rigorously vetted. And while the authors have cited some of the key publications in this arena, they have not captured the conceptual framework correctly nor accurately.

Over the last 12 years, there have been some outstanding reports on how to direct terpene metabolism in the cytosol and chloroplast compartments. I won't dwell on these but rather use a particular result presented in the current manuscript to illustrate a lack of critical consideration by the authors. Fig. 5b presents results from transiently expressing the chloroplast-targeted TS, plus and minus over-expression of early steps in the MEP and MVA pathways. TS expression by itself yields 5 µg/g FW, while TS+DXS or TS+HMGR yields 45 µg/g. Firstly, what the authors don't examine is comparison between full-length HMGR versus truncated forms. Why is this important? Well, there are many precedent studies showing that expression of the full-length (and hence ER targeted form) does not improve carbon flux while truncated forms do. Second, how do the authors account for a plastid-targeted TS's ability to gain sufficient carbon from a cytoplasmic targeted HMGR? The authors are apt to claim there is exchange between the cytosolic MVA pathway and the plastid MEP pathways. Okay, then the control, over-expression of just HMGR should enhance carotenoid levels. The converse experiment with the DXS over-expression needs an equal intensive assessment. If there is movement of MEP pathway intermediates to the cytosolic pathway, then one would not expect total sterol levels to be impacted, but sterol biosynthetic intermediates. Again, the control experiments are missing here and there isn't sufficient critical assessment of the results themselves.

A second interpretation of the Fig. 5b experiment is that there is inefficient targeting of the TS to the plastid compartment. That is, there are two populations of TS – one directed to the plastid and a second that doesn't make it to the plastid compartment. There are ways to test for this via approaches published over the years. Is this important to distinguish? Yes indeed! Because this could also be helpful in explaining some the P450 expression results. These are details that could have very important ramifications for how the data might be more thoroughly explained.

Still staying with Fig. 5b, coupled expression of the TS with GGPS gives a significant boost to production, and in combination with both HMGR or DXS gives another doubling. However, either TS-HMGR nor TS-DXS gives an equally enhanced production level as the TS+GGPS+HMGR+DXS. So, does this mean that the prenyltransferase isn't significant? I appreciate that the authors provide some mRNA level determinations, but I question the significance/importance of this. Wouldn't actual measurement of enzyme activities in the infiltrated tissue samples be more appropriate? I suspect that the level of enhanced GGPS enzyme activity is very variable. But, if its activity were significant, then elevated levels of GGOH in the tissue samples should be detectable. Were the authors able to detect GGOH?

Another figure that opens more questions than answers is Fig. 6. These panels are providing evidence that targeting of the P450 T5alphaH to the chloroplast can significantly improve the accumulation of oxidized taxanes. Some evidence that the T5alphaH targeted to the chloroplast is actually catalytically competent and active is needed. Targeting a normally cytoplasmic P450 to the chloroplast would entail the vectorialmovment of the peptide across the inner membrane, followed by its proper folding and insertion of a heme prosthetic unit. This is possible, but some evidence for such would make for a more compelling story. Equally difficult to understand is how the histobars in 6c actually correlate with GC trace in 6d. The peak for compound 3 looks to be much more significant than that for peak 2. Yet, the quantitation in the histogram is just of the inverse of the chromatogram – peak 2 is twice that of peak 3.

I've focused on a couple panels of data because I wanted to illustrate the depth of understanding I would expect in a paper suitable for a Nature specialty journal.

But there other concerns I have about the manuscript as well.

The error bars presented for all the data are incredibly tight. There are lots of reports of agroinfiltration to illustrate the variability in such data. If one were to standardize exactly what leaf was infiltrated by position on the plant and time upon when the leaf reaches maturity, and if one were able to demonstrate that they were able to infiltrate exactly the same number of CFUs, and if one were to examine just the infiltration zone only, then perhaps it is possible to obtain data with essentially less than 20% variations. But reading the methods, there isn't sufficient detail to suggest such attention to the details. Nor is there any mention of what the replicates are in each case. I'm assuming there are technical repeats within a single experiment. In fact, there should be documentation comparing between biological repeats between independent experiments.

The authors cite references which they state provide insight into the importance of compartmentalization for diterpene accumulation in N. benthamiana. When I examine those citations, at least one of the articles did not address diterpene production as suggested by the authors. If the authors cite papers it means they have rigorously read those papers and are citing them accurately. This is critical to the integrity of the scientific community.

The authors are also remiss in not mentioning some of the work on transient expression in N. benthamiana for oxidized triterpenes. Wouldn't that work be the precedent for the current work?

In summary, this manuscript represents an important approach to developing production platforms in plants, but the work is preliminary and better suited for a traditionally based specialty journal.

Point-by-point response to reviewers' comments:

General Response

We thank all three reviewers for their time and their thoughtful evaluation of our manuscript and for the very helpful and detailed comments. We have carefully considered all the comments and accordingly revised the manuscript as per the suggestions, and in addition, we carried out further experiments as suggested by the reviewers. We greatly appreciate the reviewers` help and we believe that the revision has improved the paper and clarified a number of important points. The shared points of criticism were the lack of an in-depth examination of the proposed contribution of the MVA pathway to the biosynthesis of the taxane olefin scaffold and the need for more full-proof identification of reported taxane metabolites. We appreciate the reviewers for bringing this up. We have thoroughly assessed our previous results and carried out suggested experiments, resulting in identification of a previously missed contaminating peak eluting at nearly the same retention time as taxadiene and with some similar mass spectra fragments. The contaminating metabolite increased upon overexpressing HMGR in *Nicotiana benthamiana* leaves but not to a point where the peaks could be easily separated from the taxadiene peak. Specifically, overexpression of the truncated HMGR as suggested by the reviewers enhanced the peak almost hundreds-fold and helped to identify it as a contaminating peak. As a result, we have modified our conclusions and interpretation of results accordingly.

We have provided detailed replies to all reviewers' comments in the point -by-point responses below, and we have carefully revised our manuscript keyed to the comments. Revised areas in the main manuscript and the supplementary data are in red.

Reviewer #1 (Remarks to the Author):

Comment: *If the conclusions are not original, it would be helpful if you could provide relevant references.*
The conclusions are not original, and the authors do cite some of the prior art in their references, yet have a glaring omission of the seminal work by Croteau et al., who identified and characterized the genes used later in iterations of Taxol pathway engineering efforts as follows:

Guerra-Bubb, J., Croteau, R., and Williams, R. M. (2012) The early stages of taxol biosynthesis: an interim report on the synthesis and identification of early pathway metabolites, Nat. Prod. Rep. 29, 683-696.

Kaspera, R., and Croteau, R. (2006) Cytochrome P450 oxygenases of Taxol biosynthesis, Phytochem.Rev. 5, 433-444.

Croteau, R., Ketchum, R. E., Long, R. M., Kaspera, R., and Wildung, M. R. (2006) Taxol biosynthesis and molecular genetics, Phytochem.Rev. 5, 75-97.

Dejong, J. M., Liu, Y., Bollon, A. P., Long, R. M., Jennewein, S., Williams, D., and Croteau, R. B. (2006) Genetic engineering of taxol biosynthetic genes in Saccharomyces cerevisiae, Biotechnol. Bioeng. 93, 212-224.

Jennewein, S., Park, H., DeJong, J. M., Long, R. M., Bollon, A. P., and Croteau, R. B. (2005)

Coexpression in yeast of Taxus cytochrome P450 reductase with cytochrome P450 oxygenases involved in Taxol biosynthesis, Biotechnol. Bioeng. 89, 588-598.

Chau, M., and Croteau, R. (2004) Molecular cloning and characterization of a cytochrome P450 taxoid 2alpha-hydroxylase involved in Taxol biosynthesis, Arch. Biochem. Biophys. 427, 48-57.

Jennewein, S., Long, R. M., Williams, R. M., and Croteau, R. (2004) Cytochrome p450 taxadiene 5alpha-hydroxylase, a mechanistically unusual monooxygenasecatalyzing the first oxygenation step of taxol biosynthesis, ChemBiol 11, 379-387.

Horiguchi, T., Rithner, C. D., Croteau, R., and Williams, R. M. (2002) Studies on taxol biosynthesis. Preparation of taxa-4(20),11(12)-dien-5 alpha-acetoxy-10 beta-ol by deoxygenation of a taxadiene tetraacetate obtained from Japanese yew, J Org Chem 67, 4901-4903.

Jennewein, S., Rithner, C. D., Williams, R. M., and Croteau, R. B. (2001) Taxol biosynthesis: taxane 13 alpha-hydroxylase is a cytochrome P450-dependent monooxygenase, Proc. Natl. Acad. Sci. U. S. A. 98, 13595-13600.

Walker, K., and Croteau, R. (2001) Taxol biosynthetic genes, Phytochemistry 58, 1-7.

Wheeler, A. L., Long, R. M., Ketchum, R. E., Rithner, C. D., Williams, R. M., and Croteau, R. (2001) Taxol biosynthesis: differential transformations of taxadien-5 alpha-ol and its acetate ester by cytochrome P450 hydroxylases from Taxus suspension cells, Arch. Biochem. Biophys. 390, 265-278.

Walker, K., Ketchum, R. E., Hezari, M., Gatfield, D., Goleniowski, M., Barthol, A., and Croteau, R. (1999) Partial purification and characterization of acetyl coenzyme A: taxa-4(20),11(12)-dien-5alpha-ol O-acetyl transferase that catalyzes the first acylation step of taxol biosynthesis, Arch. Biochem. Biophys. 364, 273-279.

Hefner, J., Ketchum, R. E., and Croteau, R. (1998) Cloning and functional expression of a cDNA encoding geranylgeranyl diphosphate synthase from Taxus canadensis and assessment of the role of this prenyltransferase in cells induced for taxol production, Arch. Biochem. Biophys. 360, 62-74.

Huang, K. X., Huang, Q. L., Wildung, M. R., Croteau, R., and Scott, A. I. (1998) Overproduction, in Escherichia coli, of soluble taxadiene synthase, a key enzyme in the Taxol biosynthetic pathway, Protein Expr. Purif. 13, 90-96.

Hefner, J., Rubenstein, S. M., Ketchum, R. E., Gibson, D. M., Williams, R. M., and Croteau, R. (1996) Cytochrome P450-catalyzed hydroxylation of taxa-4(5),11(12)-diene to taxa-4(20),11(12)-dien-5alpha-ol: the first oxygenation step in taxol biosynthesis, ChemBiol 3, 479-489.

Wildung, M. R., and Croteau, R. (1996) AcDNA clone for taxadiene synthase, the diterpenecyclase that catalyzes the committed step of taxol biosynthesis, J. Biol. Chem. 271, 9201-9204.

Lin, X., Hezari, M., Koepp, A. E., Floss, H. G., and Croteau, R. (1996) Mechanism of taxadiene synthase, a diterpenecyclase that catalyzes the first step of taxol biosynthesis in Pacific yew,

Biochemistry 35, 2968-2977.

Koepp, A. E., Hezari, M., Zajicek, J., Vogel, B. S., LaFever, R. E., Lewis, N. G., and Croteau, R. (1995) Cyclization of geranylgeranyl diphosphate to taxa-4(5),11(12)-diene is the committed step of taxol biosynthesis in Pacific yew, J. Biol. Chem. 270, 8686-8690.

Answer: We thank the reviewer for positive evaluation of our work. We apologize for inadvertently overlooking citation of these critical prior works especially from Croteau group and we thank the reviewer for the suggestions. As already noted by the reviewer, our conclusions derive from novel chloroplastic engineering of the partial taxol pathway and utilization of the native precursor pathways to improve isoprenoid precursor supply. In that regard and in light of the previous disappointing results in engineering the early taxol pathway in plant systems, the conclusions are original, but as suggested by the reviewer, the introduction, commentary and arguments discussed here will benefit a lot by a more focused coverage of suggested critical prior work. The engineering efforts presented in this work benefited from the cloning experiments in the suggested papers. Out of all the 18 papers suggested by the reviewer, we have now cited 14 in the revised manuscript. We excluded the other 4 excellent papers (Chau & Croteau, 2004; Horiguchi et al., 2004; Jennewein et al., 2001 and Huang et al., 1998) mainly because they described semi-chemical synthesis or they described cloning and mechanisms of enzymes that were not used in this work. The added citations are in the Introduction and Discussion sections, and we feel the paper is now more balanced, has a more solid background and the data and conclusions are in an improved context in the revised manuscript.

Comment: *This reviewer was disappointed to read that efforts to engineer the paclitaxel pathway into a heterologous host have not advancesd beyond the first 3 intermediates presumed to be committed to the Taxol pathway as shown earlier in the extensive effort by Stephanopolous with the same distribution of off-pathway by-products formed. (Ajikumar, P. K., Xiao, W.-H., Tyo, K. E. J., Wang, Y., Simeon, F., Leonard, E., Mucha, O., Phon, T. H., Pfeifer, B., and Stephanopoulos, G. (2010) Isoprenoid pathway optimization for Taxol precursor overproduction in Escherichia coli, Science 330, 70-74) and by Anterola (Anterola, A., Shanle, E., Perroud, P. F., and Quatrano, R. (2009) Production of taxa-4(5),11(12)-diene by transgenic Physcomitrella patens, Transgenic Res. 18, 655-660), as examples.*

Answer: We agree with the reviewer on the urgent need to construct the taxol pathway further downstream. As demonstrated over the past decade, the metabolic engineering of the taxol pathway is extremely challenging in heterologous hosts mostly due to the product promiscuity of T5αH and other related cytochrome P450s. It comes as no surprise that little progress has been made in recent years. The works referred to by the reviewer were breakthrough, but still faced the challenge of by-products on the oxidation chemistry of taxol biosynthesis. Although the first 3 intermediates have been produced in a coculture of *E.coli* and yeast (Zhou et al., 2015), the yields achieved were very low. All these obstacles, combined with the limited pathway knowledge currently available warrant exploration of other production platforms and engineering strategies that can be leveraged on for future efforts in both pathway elucidation and pathway engineering. Considering that taxol is a plant natural product, we developed this plant system to offer an alternative system to microbial systems and demonstrate its robustness in engineering the taxol pathway. Previous efforts to engineer the pathway in plant systems met with very limited success in taxadiene production and failed to produce taxadiene-5α-ol. Though microbial systems have had a headstart in taxol bioengineering, it is still crucial to develop alternative platforms in parallel, especially in plants.

Plant systems can provide cost-effective production as is the case in artemisinin where despite the success in engineering commercially relevant levels for artemisinic acid in *Saccharomyces cerevisiae* (Paddon et al., 2013), development of plant production platforms is still pursued (Farhi et al., 2011; Fuentes et al., 2016; Malhotra et al., 2016) due to relatively higher costs of microbial production systems (Ikram & Simonsen, 2017, Peplow 2016).

Comment: *Despite the author's excellent extraction of cDNA from T. chinesis needles, without the seminal sequence information deposited by Croteau into the DNA databanks, the characterization of their cDNA sequences would be lengthy. Along these lines, it was unclear why the authors chose to isolate RNA from the needles of the Taxus plant rather than using the abundant extant sequence information of various orthologs to synthesize the needed cDNAs.*

Answer: The choice to clone the sequences from the cDNA of *T. chinesis* needles was made purely on economic considerations. We agree the simple retrieval of deposited information of various sequences and synthesis of required cDNAs would have been easier, but cloning the sequences was considered quicker and cheaper. Considering *T. chinensis* plant resources are easily accessible and available to us, and the fact that we also aimed to screen the *T. chinensis* cDNA library for other novel cytochrome P450s in this work, we then opted for cloning, guided of course by the several sequences deposited in DNA databanks as stated by the reviewer. The cloned sequences were blasted against the deposited sequences and showed 99-100% identities.

Comment: *As I began reading the manuscript, I was felt the authors would address one of the biggest "impediments" confronted when attempting to engineer the taxol pathway into a chassis organism. The authors state:*

" the biggest impediment to achieving total biotechnological production of taxol lies in the dearth of knowledge on its complex biosynthetic pathway, which consist of at least 19 steps from GGPP..."

This reviewer's sense of the Taxol pathway is that after the first modifications of the taxadiene core early in the pathway, the order of the pathway transformations is overwhelming to predict a linear route. It is not clear how the current work described by Wang et al. addresses this impediment.

Answer: Indeed, the dearth of knowledge on the taxol biosynthetic pathway is a major obstacle on efforts to engineer the pathway in chassis organisms. As demonstrated in all previous attempts to engineer the pathway past the first committed intermediate, the order of the pathway is non-linear and complex, characterized by several off target and dead-end products. While the current work was not designed to directly tackle the selectivity of T5αH and channel more oxidised taxanes towards taxadiene-5α-ol *per se*, it provides useful insights through utilization of the native biology of a plant to synthesize taxanes. As noted by the reviewer, the statement in the introduction section might be interpreted to mean that the current work focuses on pathway elucidation to address the dearth of knowledge on taxol biosynthesis, so we have re-worded the statement to avoid this ambiguity and improve clarity. The sentence now reads: "*Despite these significant gains and the great strides that have been made toward synthetic production, the failure to achieve total biotechnological production of taxol lies in the non-effective expression of known pathway enzymes and*

the dearth of knowledge on its complex biosynthetic pathway." (Line 71 in the revised manuscript).

Comment: *Is the work convincing, and if not, what further evidence would be required to strengthen the conclusions?*
The crosstalk of the MVA and MEP pathways is compelling; however, it would be nice if the authors could do 13C-labeling studies to confirm their conclusion that both primary metabolic pathways indeed feed into the taxane diterpenes. In situ immunoblotting to identify the localization of the enzymes in planta would be beneficial. While the peptide target sequences putatively inform on the destination of the expressed enzymes, there is no guarantee that they are transported exclusively to the target organelle. If they (GGPP synthase, TS, T5OH) are distributed amongst organelles and the cytosol where the MVA and MEP pathway are functioning, then one may observe the unusual "crosstalk" proposed by the authors. Described in its current state, the conclusion is only speculative. The latter could be studied by in situ immunoblotting.

Answer: We thank the reviewer for this constructive criticism and these excellent suggestions for experiments to strengthen our conclusion. We completely agree that ^{13}C-labeling studies would directly confirm the involvement of isoprenoid precursor pathways. While the experiment seems not technically challenging, the unavailability of labeled CO_2 in our area and complications of its importation hindered our efforts. In the absence of labeled CO_2, we attempted feeding ^{13}C-glucose by direct foliar application and through supplementation in water but the synthesized taxadiene was not labeled. We then developed TS stably-transformed *N. benthamiana* explants (see additional Materials and Methods section and Supplementary Fig. S16) and attempted feeding labeled glucose from supplemented MS media. Though the feeding was partially successful (see Fig. R1 below) the labeling pattern was not conclusive. The m/z 274 in Fig. R1B indicates taxadiene synthesized from 3 normal IPP and one labeled [1,5-^{13}C]IPP (MEP-labeled) and the m/z 275 indicates taxadiene from 3 normal IPP and one labeled [2,4,5-^{13}C]IPP (MVA-labeled). Because the plants were grown on sterile media, we could not transiently infiltrate Agrobacteria with DXS or HMGS, so we resorted to inhibition of the MVA or the MEP pathway using lovastatin and fosmidomycin, respectively (inhibition experiments are described in our response to reviewer # 3, page 21-22 in this rebuttal document). Inhibition of the MEP pathway completely blocked taxadiene formation, thus labeling experiments could not be conducted. Inhibition of the MVA pathway resulted in some alterations in the abundance of fragments compared to unblocked plants (Fig. R1C-D). For example, lovastatin treatment decreased the abundance of m/z 275 in Fig. R1D, indicating that the MVA somehow interacted with taxadiene formation, however the observed pattern was not enough to draw inferences as to the source of precursors and the contribution of each pathway. We tried our best over the past months, but unfortunately, we successively failed to label patterns that would have provided direct proof of the involvement of the MVA or the MEP pathway.

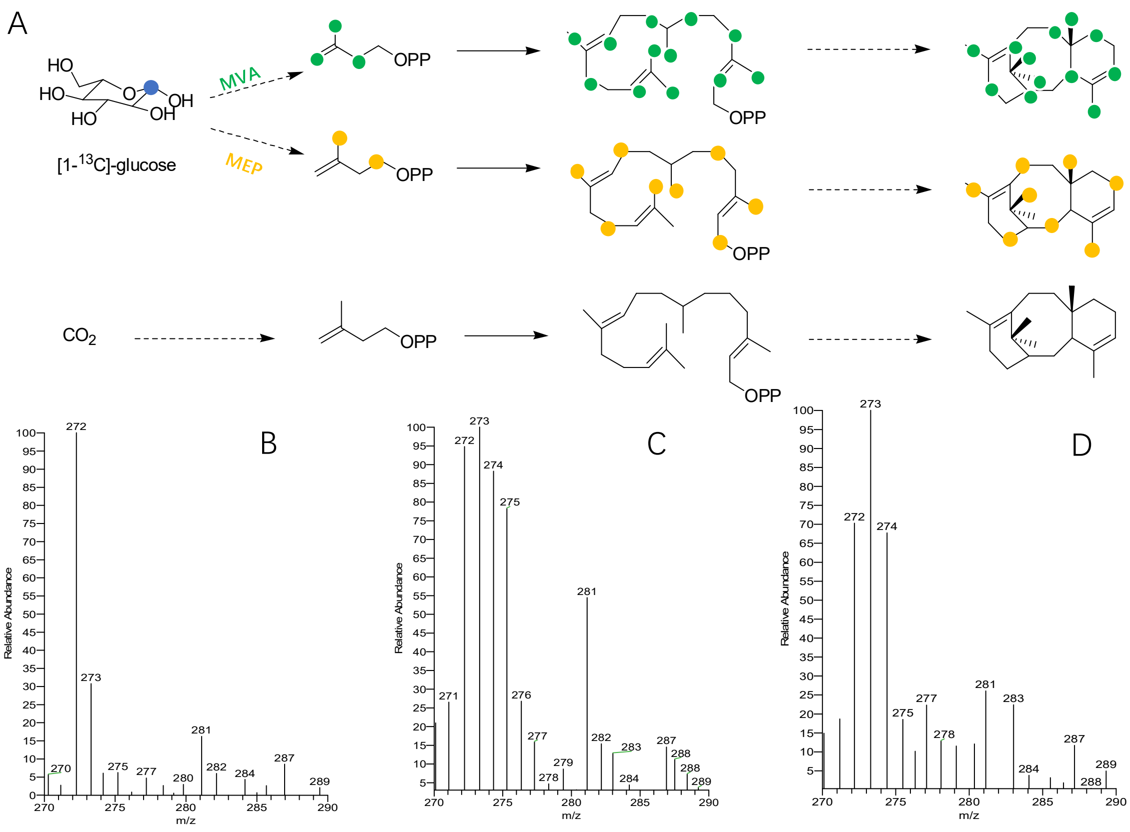

Figure R1. Taxadiene labeling in TS-transformed tobacco line NTS1-T2. (A). Differential labeling patterns of taxadiene from MEP or MVA pathway; Taxadiene from [2,4,5-^{13}C]IPP via the MVA pathway and [1,5-^{13}C]IPP through MEP pathway are distinct (Wu et al., 2006). (B). Normal glucose as the sole carbon source; (C). [1-^{13}C]glucose as the sole carbon source; (D) [1-^{13}C]glucose as the sole carbon source and supplemented with lovastatin to block the MVA pathway.

We however designed another set of experiments to ascertain the contribution of the two-isoprenoid precursor pathways via overexpression of both HMGR and tHMGR (its truncated version) in TS-transgenic plants and in wild plants as controls (also suggested by Reviewer 3, who also raised a similar point). In our previous version, we speculated that both the MVA & MEP pathways are involved in taxane biosynthesis based on the observation that overexpression of both DXS and HMGR enzymes improved taxadiene production by comparable levels (Supplementary S14, and S15). We once again repeated the experiment very carefully, and aided by the expression of tHMGR and the addition of aforementioned controls in wild type *N. benthamiana* plants, we identified a previously missed contaminating peak that elutes very close to taxadiene and have some similar mass fragments (see attached data below).

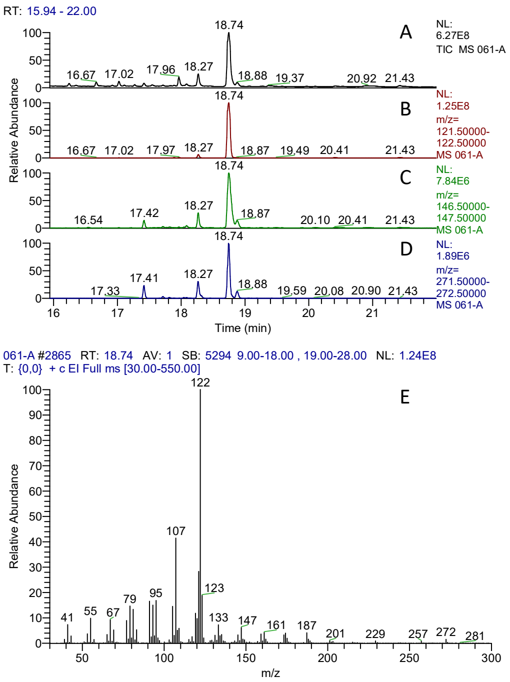

Figure S14. GC-MS analysis of taxadiene with transient co-expression of DXS and TS in N. benthamiana leaves. (A) TIC of hexane extract of DXS and TS transient co-expression leaves; (B) EIC for m/z+ 122 representing taxa-4(5), 11(12)-diene; (C) EIC for m/z+ 147 representing a putative unknown compound; (D) EIC for m/z+ 272 stands for the taxadiene and its analogs; (E) Mass spectrum of peak (tR 18.74) representing the taxa-4(5),11(12)-diene.

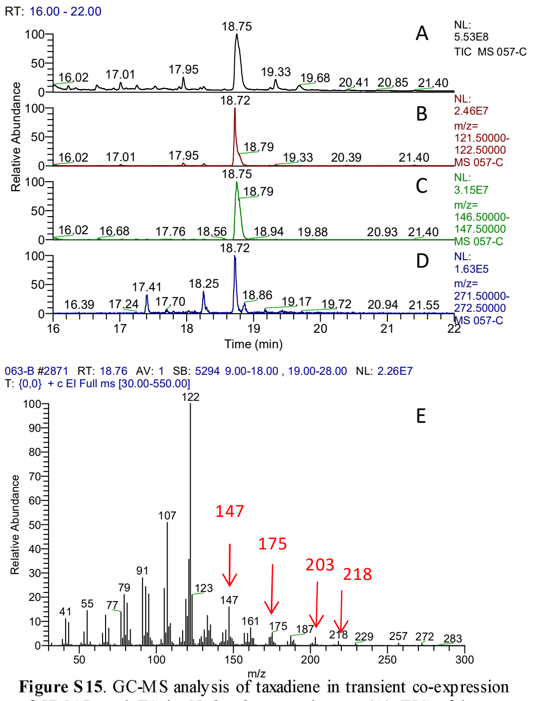

Figure S15. GC-MS analysis of taxadiene in transient co-expression of HMGR and TS in *N. benthamiana* leaves. (A) TIC of hexane extract of HMGR and TS transient co-expression leaves; (B) Extracted Ion Chromatography for m/z$^+$ 122 stands for taxa-4(5), 11(12)-diene; (C) EIC for m/z$^+$ 147 representing a putative unknown compound; (D) EIC for m/z$^+$ 272 representing for the taxadiene and its analogs; (E) Mass spectrum of the peak at 18.75 min. The retention time and the mass spectrum are very similar with the authentic taxa-4(5),11(12)-diene. And a contaminate compound was wrapped with taxadiene in the latter experiment with the overexpressing of HMGR/tHMGR in NTS1-T2 and wild type tobacco.

In our previous data, taxadiene had a retention time of 18.74 min, and the contaminant had a retention time of 18.75 min, and also shows m/z of 147 and 122 making it less obvious to distinguish from taxadiene at low concentrations (Supplementary S14, and S15), leading to the inadvertent identification of the coeluting contaminant as taxadiene in our previous data. From the new experiments, we show that the contaminant increased nearly 10-fold upon expression of tHMGR compared with full-length HMGR (The taxadiene has a retention time of 18.60 min, the contaminant has a retention time of 18.65 min, Supplementary S18, and S19 below). Expression of HMGR/ tHMGR in control wild-type tobacco also resulted in production and improvement of this contaminant peak [the contaminant peak gave the m/z 218, 203, 175, 147 (Supplementary Fig. S20 below), which can be also be found from the mass spectrum of HMGR-TS expressed leaves in Supplementary S15], clearly demonstrating that this is a native metabolite apparently with no established link to the introduced heterologous pathway. Based on this new data, we have corrected our conclusion that taxadiene is predominantly derived from the MEP pathway in *N. benthamiana*.

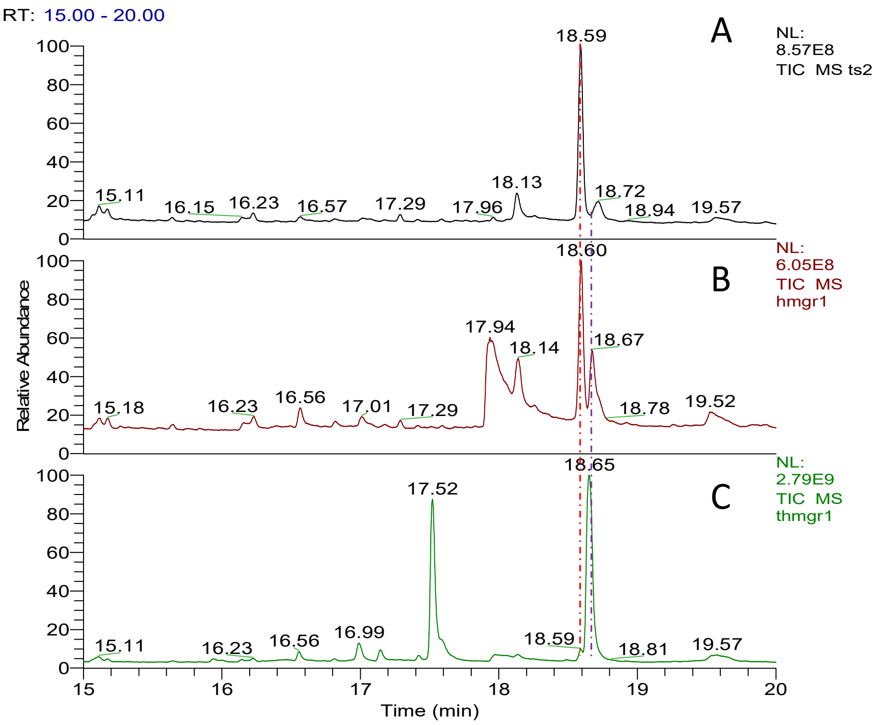

Figure S18. GC-MS analysis of taxadiene (18.60 min) with transient overexpression of HMGR and tHMGR in TS transgenic tobacco leaves. (A) TIC of transgenic tobacco leaves; (B) TIC of transgenic tobacco leaves with HMGR transient expression; (C) Transgenic tobacco leaves with tHMGR transient expression. The peak at 18.60 min representing taxa-4(5),11(12)-diene (red dashed line), and the peak at 18.65 representing a putative unknown compound (purple dashed line).

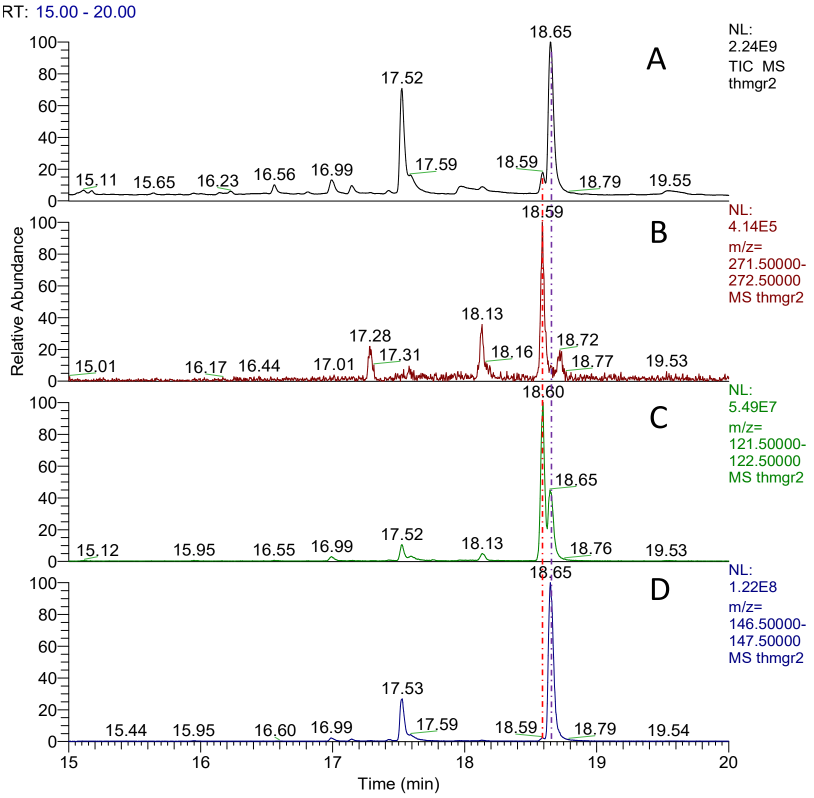

Figure S19. GC-MS analysis of metabolite in transient tHMGR expression in TS transgenic tobacco. (A) TIC of extracts from tHMGR expressing NTS1-T2 tobacco; (B) SIM of m/z 272 representing taxadiene; (C) SIM of m/z 122 representing the major fragment of taxadiene; (D) SIM of m/z 147 representing the major fragment of the native contaminant of tobacco. The peak at 18.60 min representing taxa-4(5),11(12)-diene (red dashed line), and the peak at 18.65 representing a putative unknown compound (purple dashed line).

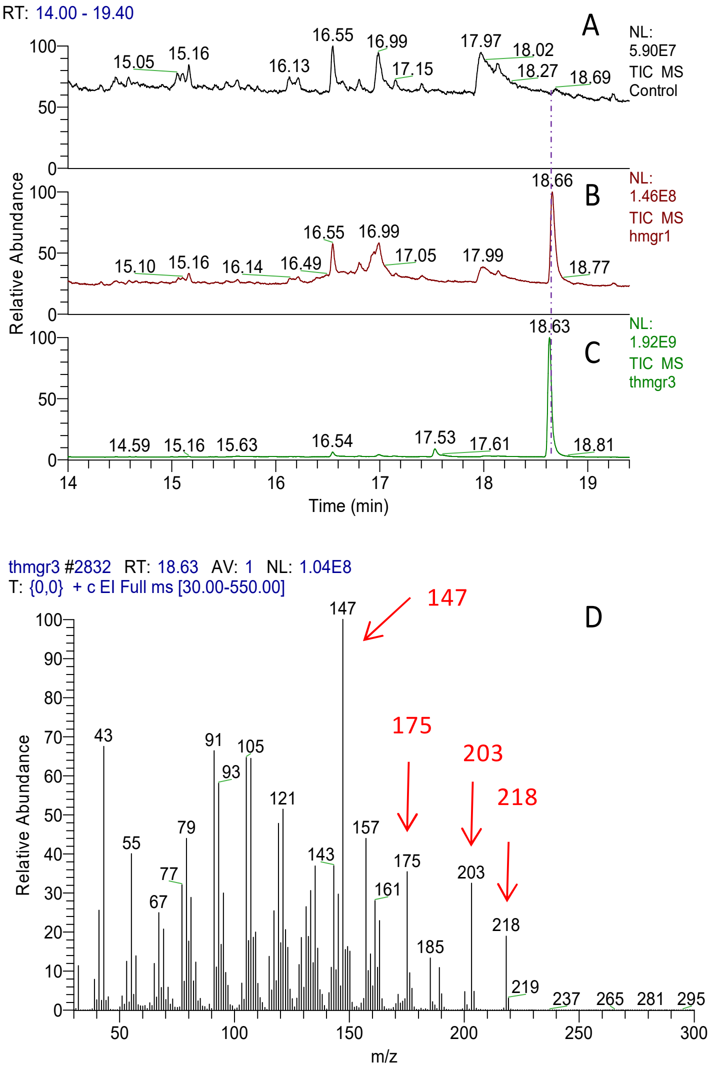

Figure S20. GC-MS analysis of metabolites with transient expression of HMGR and tHMGR in wide type tobacco leaves. (A) TIC of extracts from wild type tobacco.; (B) TIC of extracts from HMGR expressing tobacco; (C) TIC of extracts from tHMNGR expressing tobacco. A trace amount of the unknown peak can be measured, the yield can be improved with the expression of HMGR and greatly improved with the expression of tHMGR; (D) Mass spectrum of the unknown compound.

In addition, we also carried out inhibition experiments of the MEP and the MVA pathways in stable-transformed tobacco lines to further support this conclusion (see our response to Reviewer 3, and Supplementary Fig. S17).

Another question pertains to the localization of fusion proteins to target organelles. We agree that insertion of target chloroplastic transit sequences on fusion proteins is no guarantee of complete localization in target plastids. We have carried out additional fluorescence assays using a cyan fluorescent protein (CFP) infused at the C-terminal of tp(TS)-trT5H-trCRP to detect cellular location of the fusion protein. Results clearly show that the final chimera was located in the chloroplasts (see the added panel, Fig. 3C).

Comment: *On a more subjective note, do you feel that the paper will influence thinking in the field?*
This reviewer feels that the paper uses the taxol pathway genes as a model to demonstrate the utility of Nicotiana benthamiana as a chassis host, and this helps others in the area of plant

host engineering. Their understanding of organellar targeting in planta is another major consideration that informs other researchers in the biotechnological area. To contrast, this manuscript unfortunately does not advance the area of Taxol biosynthesis as suggested throughout the document.

Answer: We thank the reviewer for positive evaluation of our work. As explained in the above comments to the reviewer, though the current work was not designed to directly tackle pathway elucidation, the novelty of the work lies in the engineering strategies presented and the use of the plant system that could provide an easy alternative platform to (i) further engineer the pathway and (ii) elucidate the pathway through identification of intermediates and screening of missing pathway genes, especially plant Cytochrome P450s in the future. By successfully producing taxadiene-5α-ol to relatively high levels in a plant system without addition of carbon feedstocks and extensive engineering of the pathway, this work is not just proof of concept but demonstrates a useful strategy and advancement of engineering efforts in plant cells.

Reviewer #2 (Remarks to the Author):

Comment: *However, the author has not provided the real ratio of the OCT: taxadiene 5-ol. Also, the GC-MS method used for the identification of the product is not full proof and the authors need to produce more product using this plant source and at least provide NMR for identification of the products as was done in the ACS chemical biology paper by Ajikumar's group. While the engineering in this paper is very novel, the full characterization of the products in required and quantification of the ratio of OCT: 5-alpha ol is important.*

Answer: The reviewer raises a very important point in light of the taxadiene-contaminating metabolite that we uncovered in the additional experiments conducted during the course of this revision (see our response to reviewer #1 above). We have purified the main compound taxa-4(5), 11(12)-diene from the DXS-GGPPS-TS overexpressing tobacco leaves and in addition to GC-MS, we also identified the compound by ^{1}H and ^{13}C nuclear magnetic resonance (NMR) spectroscopy studies as suggested. The NMR data (see Supplementary Fig. S9-S12) for taxadiene was in agreement with published data (Rubenstein and Williams., 1995; Koepp, et al., 1995). The related experimental section has been described in the supporting material and the result has been added in the main manuscript (L167). We also aimed to purify and identify the oxidised taxanes (taxadiene-5α-ol, OCT and iso-OCT) by NMR, but unfortunately failed to isolate enough amounts of the compounds due to the relatively low yields in engineered tobacco leaves. As for OCT (the oxygenated product that accumulated to highest levels in tobacco leaves in our study), it is worth to note that previous attempts to purify the compound for NMR analysis was met with stability issues by Ajikumar and coworkers (Biggs et al., 2016). However, we have carefully checked the MS spectrum of the designated oxidised compounds and compared with the spectra reported by Hefner et al., (1996) and papers from Ajikumar's group (Biggs et al., 2016) that confirmed an exact match to taxadiene-5α-ol.

We have added descriptions of the ratios of taxadiene-5α-ol, OCT and iso-OCT yields in the result section (see L238, L345). Cytosolic engineering of the pathway resulted in taxadiene-5α-ol : OCT : iso-OCT ratios of 1 : 5 : 1.5, while chloroplastic engineering coupled with isoprenoid precursor enhancement slightly shifted the product ratio to 1 : 4 : 1.6, respectively. This product profile is different from the one reported in microbial systems (Sagwan-Barkdoll et al., 2010) where taxadiene-5a-ol contributed to much lower percentage of the total oxidised taxanes.

Comment: *Another thing the authors can try is to use the reductase of the nicotiana plant which may further increase the production.*

Answer: Thanks for this advice. We did try using the endogenous CPR in *Nicotiana benthamiana* without expression of heterologous TcCPR. However, there was no target compound detected. In light of this observation, overexpression of *Nicotiana* CPR was not attempted.

Comment: *Minor comments: while the manuscript is very well written, the figure quality needs to improved. The GC-MS data is very noisy. Moreover, the authors should include better characterization of the products.*

Answer: We apologize for the poor quality of the figures, especially the mass spectra. We agree the mass spectra appear a bit noisy, most this was likely due to the background of tobacco metabolites. We have improved the figures by adding peak labels and have reworked all the other figures in terms of presentability and hope they now have a better appeal in the revised version.

***Comment**: (Lines 144-147):"TS detectable at 2 dpi and accumulated to its highest level at 4 dpi followed by a slow decrease afterwards. CPR showed a similar accumulation profile to that of TS. On the other hand, T5αH was detectable at 2 dpi and continuously increased through 5 dpi." This was the only point in the manuscript that the authors stated anything about temporal relationships with gene/protein expression, aside from their arguments about limiting carebon flow. Though the authors examined transcriptional levels of MEP and MVA pathway genes (Figure 5a), expression levels as measured by qRT-PCR were only included up to Day 3, leaving this earlier comment in the Results section unaddressed. The authors should revise Figure 5a by including expression values throughout days 1-5 -post transformation.*

Answer: The qRT-PCR experiments were designed to probe the innate isoprenoid precursor supply pathways and identify bottlenecks limiting carbon supply to taxane biosynthesis following introduction of the heterologous pathway. For that purpose, the early period after infiltration was considered critical and more informative, thus we only checked the first three days post-infiltration. We considered temporal expression at protein level to be more appropriate for describing the late events, thus we conducted protein level analyses on days 4 and 5, together with day 2 to provide a reference point for comparison. From the point of view of identifying upstream targets for engineering taxanes, and describing changes in protein expression in the late stages until harvesting, these sampling points we considered adequate. Moreover, because different leaves show some variability in mRNA levels at a given time, we carefully selected the healthiest looking plants, and avoided damaged leaves for analyses. With these stringent conditions, only a limited number of leaves (2-3 ideal leaves per plant) could be harvested for these analyses, thus we had to select time points that reflected the best changes for mRNA and protein expression.

***Comment:** It would also be very interesting if the authors to include nuclear run-on transcription assays to measure RNA accumulation for each compartmentalized gene of interest (supplemental).*

Answer: **T**hanks for this suggestion. Nuclear run-on transcription assays would be very informative if the foreign genes were integrated in the genome of the host. In our case, we did transient protein expression, thus the foreign genes do not replicate and are eventually lost through cycles of cell division over several days. We thus resorted to a description of temporal changes in mRNA and protein levels as described above.

Comment: *The authors do a fantastic job emphasizing the difficulties in navigating the spatial regulation within plant metabolomics capable of complicating engineering efforts. Attention should also be made to the temporal regulation of this metabolic flux, where discussion on participatory gene expression trends could be added. This commentary would greatly improve the present manuscript by suggesting ways for optimizing future metabolic engineering efforts when also considering the time scales of carbon flux, giving the reader improved perspective on the complexity behind the regulation of plant secondary metabolism.*

Answer: We thank the reviewer for this suggestion that definitely improves our manuscript and the discussion on complex temporal regulation encountered in plant systems and suggestions for next steps to improve pathway engineering. To give the reader an improved appreciation of the regulatory complexities behind plant metabolic fluxes, we have added commentary on temporal gene and protein expression trends in the discussion as follows:

"The metabolic flux in plants is tightly controlled by complex regulatory mechanisms, and an improved understanding of gene and protein expression trends is critical for precise control and fine-tuning of engineered pathways. Gene expression analysis of native MVA and MEP pathway genes in the early period following agroinfiltration demonstrated varying trends, implying internal adjustment and transcriptional regulation of precursor pathways. We targeted genes that decreased mRNA expression levels for overexpression and achieved an 8-fold improvement in taxadiene and oxidised terpenes, demonstrating the importance of an improved understanding of temporal regulation. Recently, the utility of precise temporal expression of taxadiene synthase and GGPPS was demonstrated in *Pichia pastoris* (Vogl et al., 2018) using bidirectional promoters designed to be induced at different time-scales that achieved a 50-fold taxadiene production compared to constitutive expression." (L475 in the revised manuscript).

Reviewer #3 (Remarks to the Author):

Comment: *While the current report does provide some intriguing observations, the work does not stand up to the expectations of a break-through report in a Nature specialty journal and the results per se are somewhat preliminary and not rigorously vetted. And while the authors have cited some of the key publications in this arena, they have not captured the conceptual framework correctly nor accurately.*

Answer: We deeply thank the reviewer for positive evaluation of our work and for raising these constructive criticisms. We have carefully carried out additional experiments to rigorously vet our results and strengthen the conclusions reported in this work. Over the past decades, many efforts to engineer the taxol pathway in heterologous hosts have been faced with several challenges, warranting establishment of alternative production platforms and novel engineering strategies to overcome these hurdles. We believe elucidation of the biosynthetic pathway, a comprehensive understanding of underlying mechanisms of enzymes and pathway regulation and an improved understanding of the fundamental biology of chassis organisms are all critical steps towards sustainable production of taxol at clinically-relevant quantities in engineered organisms. This work addresses some of these areas by designing a strategy to construct the pathway for production of the first oxidised taxanes in a plant system. The reported yields are relatively high and we believe the strategy, findings and discussions herein will benefit the broad readership of the journal and can be pave way towards pathway elucidation and future engineering efforts to overcome the decades-long hurdles encountered in microbial systems.

Regarding the accuracy and clarity in capturing the conceptual framework, we have reworked the manuscript and citations to improve clarity, contextual accuracy and logical flow of the story in the revised manuscript as detailed in the specific responses to the reviewer below.

Comment: *Over the last 12 years, there have been some outstanding reports on how to direct terpene metabolism in the cytosol and chloroplast compartments. I won't dwell on these but rather use a particular result presented in the current manuscript to illustrate a lack of critical consideration by the authors. Fig. 5b presents results from transiently expressing the chloroplast-targeted TS, plus and minus over-expression of early steps in the MEP and MVA pathways. TS expression by itself yields 5 µg/g FW, while TS+DXS or TS+HMGR yields 45 µg/g. Firstly, what the authors don't examine is comparison between full-length HMGR versus truncated forms. Why is this important? Well, there are many precedent studies showing that expression of the full-length (and hence ER targeted form) does not improve carbon flux while truncated forms do. Second, how do the authors account for a plastid-targeted TS's ability to gain sufficient carbon from a cytoplasmic targeted HMGR? The authors are apt to claim there is exchange between the cytosolic MVA pathway and the plastid MEP pathways. Okay, then the control, over-expression of just HMGR should enhance carotenoid levels. The converse experiment with the DXS over-expression needs an equal intensive assessment. If there is movement of MEP pathway intermediates to the cytosolic pathway, then one would not expect total sterol levels to be impacted, but sterol biosynthetic intermediates. Again, the control experiments are missing here and there isn't sufficient critical assessment of the results themselves.*

Answer: We appreciate this critical assessment of our results and conclusions and we thank the reviewer for the helpful suggestions that allowed us to conduct additional experiments to improve the manuscript. This was one of the shared criticisms of this work (it was also raised by reviewer #1), so we initially responded to this issue in the general response and in detail to our response to reviewer #1 above. Following the suggestions of the reviewer and further suggestions by reviewer #1, we designed three sets of additional experiments to critically scrutinize our initial conclusion on the involvement of both the MEP and the MVA pathways in supplying carbon precursors for biosynthesis of taxadiene, *viz* (1) ^{13}C-labeling studies, (2) overexpression of both HMGR and the truncated version (tHMGR) and (3) pathway inhibition studies of the MEP and the MVA pathways. As detailed in our response to reviewer #1 (pages 11-15 in this rebuttal document), our in vivo feeding experiments using ^{13}C-labeled glucose were unfortunately not conclusive to determine the contribution of each pathway towards taxane biosynthesis. We also considered labeling from isotopic CO_2, however, we failed to conduct these experiments due the unavailability of isotopically labelled gas at our disposal.

We have also detailed the results of overexpression of HMGR and tHMGR in our response to reviewer #1 above, and will describe these data in brief here. Careful repetitions of the HMGR overexpression in TS-transgenic plants, aided by the suggested expression of a more robust tHMGR resulted in identification of a previously missed contaminating peak that elutes very close to taxadiene and have some similar mass fragments (see data attached to response to reviewer #1; Supplementary Fig. S18, S19). Based on the MS spectra from previous analysis, taxadiene has a retention time of 18.74 min, and the contaminant has a retention time of 18.75 min, and also shows m/z of 147, 122, making it less obvious to distinguish from taxadiene at low concentrations. As suggested by the reviewer, we additionally conducted control experiments (overexpression of HMGR and tHMGR in wild-type tobacco). We show that the contaminant increased almost 10-fold upon expression of tHMGR compared with full-length HMGR (Supplementary Fig. S20), clearly demonstrating that the compound is a native metabolite apparently with no established link to the introduced heterologous pathway. Based on this new data, we have corrected our conclusion that taxadiene is predominantly derived from the MEP pathway in *N. benthamiana*.

The third set of experiments were contacted on TS stably-transformed tobacco lines, involving separate inhibitions of the MEP and the MVA pathways by 150 µM fosmidomycin and 10 µM lovastatin, respectively. Inhibition of the MEP pathway by fosmidomycin completely inhibited taxadiene formation (Figure S17F) while inhibition of the MVA pathway did not abolish taxadiene biosynthesis in transgenic leaves. This data is again clear evidence to support that the taxane scaffold derives predominantly from the plastid-localised MEP pathway. We have added HMGR/tHMGR overexpression and pathway inhibition data to the Supplementary information and added descriptions of these data in Results and Discussion (L424-441) sections in the revised manuscript.

Concerning the changes in chlorophylls and carotenoids:
To further test for the cross-talk we previously speculated, we did examine the phenotypes, chlorophyll and carotenoid levels of MEP and MVA inhibited transgenic tobacco plants (Supplementary Fig. S17, below). As expected, inhibition of the MEP pathway resulted in decreased carotenoids and chlorophylls while inhibition of the MVA pathway reduced sterol content, manifesting in phenotypes with shorter root length. These results are consistent with above experiments and do not support significant crosstalk of isoprenoid precursors between the chloroplast and the cytoplasm compartments. These data have also been added to the revised manuscript.

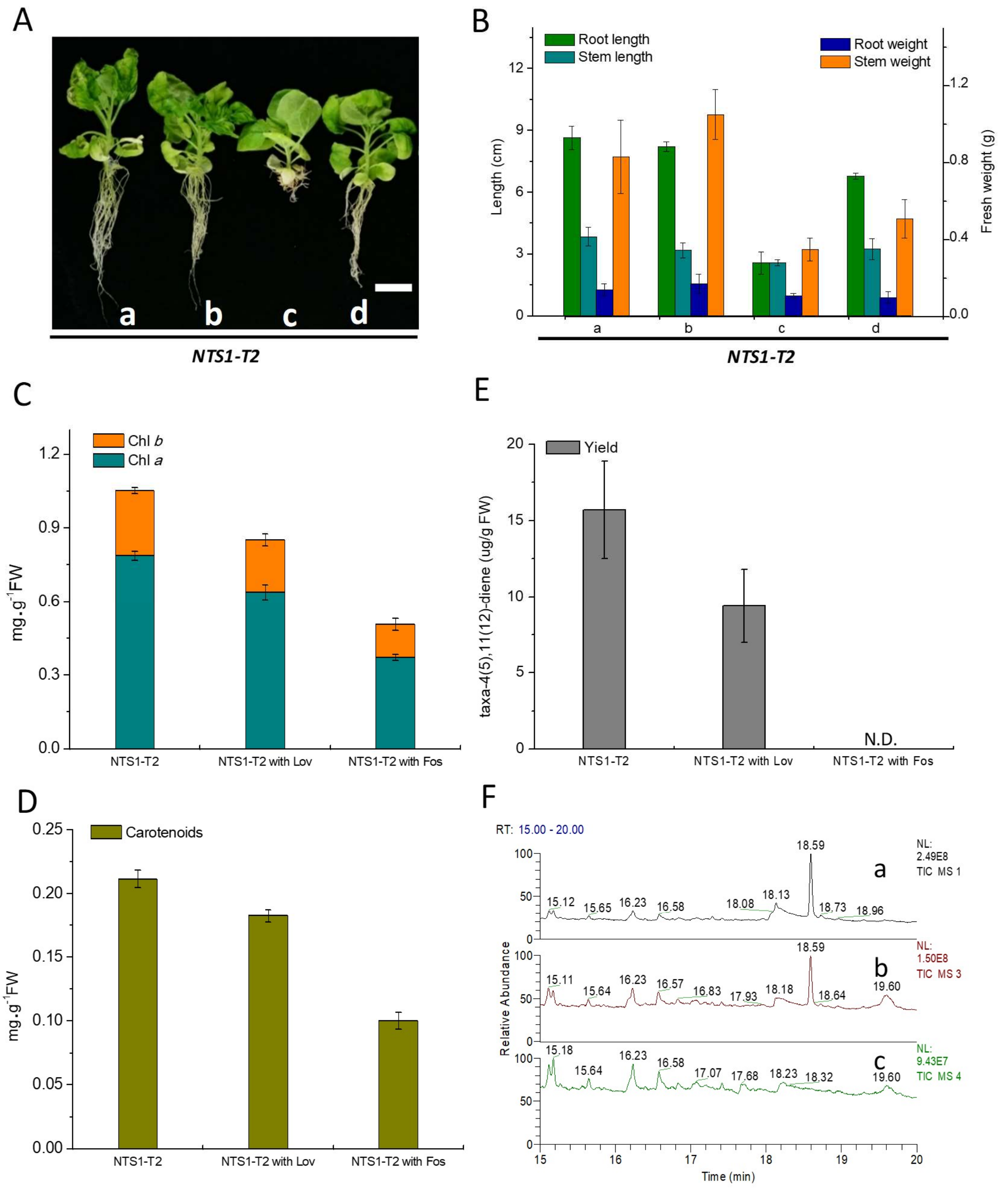

Figure S17. Phenotypes of transgenic *N. benthamiana* plants expressing TS on MS solid media with 0.3% (w/v) sucrose as the sole carbon source. (A) Phenotype of transgenic tobacco line NTS1-T2 after 28 days with/without treatment in ½MS media. From germination seedings (a); shoot from transferred seeding (b); transferred shoot with lovastatin (c); with fosmidomycin (d). bar = 2.0 cm. (B) Effect of lovastatin and fosmidomycin on root and stem development; (C-D) Effect of lovastatin and fosmidomycin on yields of chlorophyll a, b, and total carotenoids; (E) Effect of lovastatin and fosmidomycin on production of taxadiene; N.D. indicates not detected. (F) GC-MS analysis of taxa-4(5),11(12)-diene in NTS1-T2 line plant. (a) NTS1-T2 without treatment; (b) treated with lovastatin; (c) treated with fosmidomycin. The peak (tR =18.59 min) stands for the desired product taxa-4(5),11(12)-diene. Chl*a*, Chlorophyll a; Chl*b*, Chlorophyll b.

Comment: *A second interpretation of the Fig. 5b experiment is that there is inefficient targeting of the TS to the plastid compartment. That is, there are two populations of TS – one directed to the plastid and a second that doesn't make it to the plastid compartment. There are ways to test for this via approaches published over the years. Is this important to distinguish? Yes indeed! Because this could also be helpful in explaining some the P450 expression results. These*

are details that could have very important ramifications for how the data might be more thoroughly explained.

Answer: As described above, the additional experiments carried out discounted our earlier interpretation of the data on cytosolic engineering of the taxol pathway. We appreciate the reviewer for raising this issue. Regarding possible incomplete targeting of heterologous genes in the compartments, we have carried out an additional experiment with fluorescence imaging using cyan fluorescent protein (CFP) infused at the C-terminal of tp(TS)-trT5H-trCRP. We agree inserting a taxadiene synthase chloropastic targeting signal sequence (tp) to a protein naturally localized in other compartments is not full guarantee that the protein will be entirely targeted in the plastids as intended. Results of the additional experiment conducted clearly show that the final chimera was located in the chloroplasts (see the added panel, Fig. 3C). The description of this data was added to the manuscript L218-224)

Comment: *Still staying with Fig. 5b, coupled expression of the TS with GGPS gives a significant boost to production, and in combination with both HMGR or DXS gives another doubling. However, either TS-HMGR nor TS-DXS gives an equally enhanced production level as the TS+GGPS+HMGR+DXS. So, does this mean that the prenyltransferase isn't significant?*

Answer: Overexpression of GGPPS increased the yield of taxadiene up to 4-fold compared to TS overexpression alone, highlighting the significance of GGPPS in channelling isoprenoids towards diterpenoid synthesis. Co-overexpression of DXS with TS resulted in an 8-fold increase in taxadiene content compared to TS alone. The plants overexpressing DXS-GGPPS-TS accumulated taxadiene to approximately 10-fold better than TS alone. Given that overexpressing DXS alone achieved an 8-fold increase, the further boost in taxadiene of adding GGPPS was less than expected, but does not imply the prenyltransferase is not significant. We interpreted this to show that overexpressing GGPPS relieved the major pathway bottleneck in these transgenic plants as is the case in wild-type tobacco, and that further improvement will entail enhancement of the upstream precursor supply pathways. We are not claiming there is no room to further optimise the GGPPS-catalyzed step, but in our engineered plants, it's reasonable to direct efforts to further boost production of taxadiene to processes upstream or downstream of GGPPS until the prenyltransferase becomes limiting.

Comment: *I appreciate that the authors provide some mRNA level determinations, but I question the significance/importance of this. Wouldn't actual measurement of enzyme activities in the infiltrated tissue samples be more appropriate? I suspect that the level of enhanced GGPS enzyme activity is very variable. But, if its activity were significant, then elevated levels of GGOH in the tissue samples should be detectable. Were the authors able to detect GGOH?*

Answer: We examined the mRNA levels to detect the bottlenecks of the terpenoid synthetic pathway after the exogenous taxane pathway has been introduced into the cells. The production of taxadiene would exhaust the native terpenoid pool, and disturb the isoprenoid balance. Measurements of mRNA levels was very important in the design of our engineering strategy given the largely unsuccessful previous attempts to engineer the taxane pathway in plants. Based on insights from mRNA data, we streamlined this design for successful reconstitution of the pathway in a plant- based system. The suggested protein activity level assays of GGPPS are a set of experiments that would definitely improve insights on our

engineering strategy and guide next steps in pathway optimization of this design. Focus on GGPPS in heterologous pathway engineering in yeast have proved the utility of the prenyltransferase step in improving taxadiene levels (DeJong et al., 2006; Ding et al., 2014; Engels, Dahm, & Jennewein, 2008). Increases in geranylgeraniol have been reported in yeast construct following overexpression of GGPPS (up to 33mg/l in Engels and coworkers study), however, in this study, we did not detect accumulation of geranylgeraniol in our GC-MS analysis. This might reflect the differences in our system and the yeast expression systems, and the complexity of the background of the plant tissue extract used here.

Another possible reason is that GGOH would be consumed as a common precursor to the chlorophyll a/b and carotenoids generation, different from aforementioned engineered yeast systems. Analysis of chlorophyll and carotenoids in TS-overexpressing leaves (Fig. R2 below) suggests increased utilization of native isoprenoid precursors towards taxadiene biosynthesis that drastically reduced the concentrations of these compounds in engineered plants compared to the wild-type plants.

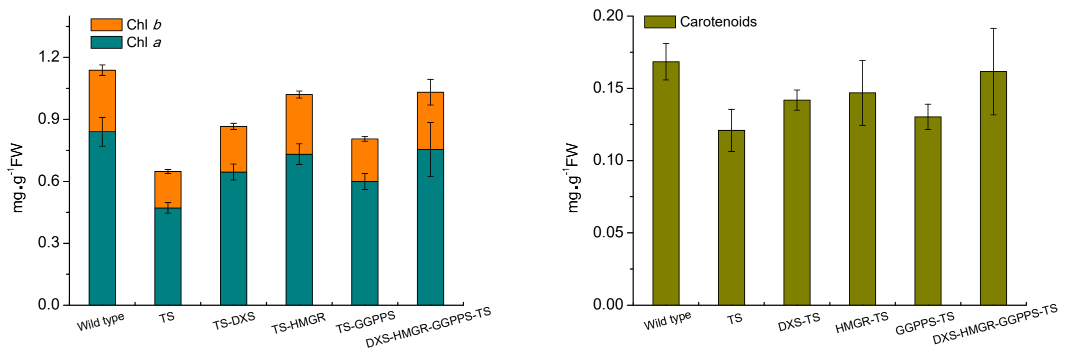

Figure R2. Analysis of chlorophyll a/b and carotenoids in wild-type and engineered tobacco plants overexpressing TS and upstream genes. Error bars indicate standard deviation of three biological replicates

Comment: *Another figure that opens more questions than answers is Fig. 6. These panels are providing evidence that targeting of the P450 T5alphaH to the chloroplast can significantly improve the accumulation of oxidized taxanes. Some evidence that the T5alphaH targeted to the chloroplast is actually catalytically competent and active is needed. Targeting a normally cytoplasmic P450 to the chloroplast would entail the vectorial movment of the peptide across the inner membrane, followed by its proper folding and insertion of a heme prosthetic unit. This is possible, but some evidence for such would make for a more compelling story.*

Answer: We used a simple strategy for targeting of the P450 enzyme T5αH fused with its reductase partner to the chloroplast by replacing the native peptides with the plastid-targeting terminal of TS. We detected the final chimera tp(TS)-trT5H-trCPR-CFP in the chloroplast, confirming proper localization. Activity assays to confirm that T5αH is catalytically competent and active are very important not only to guide optimization of engineering efforts but to make it possible to make comparisons with published T5αH kinetic parameters. However, purification and activity assays for T5αH are extremely challenging, even in microbial systems as illustrated by recent elegant designs using lipid nanodisc technology (Biggs et al., 2016; Rouck et al., 2017) to characterize this P450 enzyme. Compared to yeast microsome extracts used in the aforementioned studies, the plant tissue extract in this study is way more complex, thus, our attempts on T5αH activity assays faced several technical hurdles and were not successful. While optimization of the purification protocol of the heterologous proteins is work in progress, identification of the proteins by fluorescence assays and emergence of new, expected peaks in transgenic plants is proof (at least in part) that the introduced proteins were

active. Moreover, the ratio of taxadiene-5α-ol: OCT: iso-OCT in chloroplastic-engineered lines (1 : 4 : 1.6) was nearly similar to that in cytosolic engineered-plants, demonstrating that T5αH directed into the chloroplast can achieve a comparable product profile with T5αH in its natural environment on the ER membrane. (The preceding sentence was added in the revised manuscript; L344). The mechanism by which P450 proteins cross the two membranes of chloroplast and acquire catalytic competency through proper folding and insertion of a prosthetic heme are not yet properly defined, but the concept have been demonstrated before with even longer pathways involving more than one P450 enzyme (Gnanasekaran et al., 2016; Nielsen et al., 2013).

Comment: *Equally difficult to understand is how the histobars in 6c actually correlate with GC trace in 6d. The peak for compound 3 looks to be much more significant than that for peak 2. Yet, the quantitation in the histogram is just of the inverse of the chromatogram – peak 2 is twice that of peak 3.*

Answer: We sincerely apologize for this genuine mistake on our part. This was a genuine mistake and mix-up of figure legends. We thank reviewer for noting this, the figure has been corrected. We have also carefully re-checked all the figures, tables and Supplementary data to make sure there are no more any mistakes of this nature and to ensure accuracy in the revised manuscript.

Comment: *The error bars presented for all the data are incredibly tight. There are lots of reports of agroinfiltration to illustrate the variability in such data. If one were to standardize exactly what leaf was infiltrated by position on the plant and time upon when the leaf reaches maturity, and if one were able to demonstrate that they were able to infiltrate exactly the same number of CFUs, and if one were to examine just the infiltration zone only, then perhaps it is possible to obtain data with essentially less than 20% variations. But reading the methods, there isn't sufficient detail to suggest such attention to the details. Nor is there any mention of what the replicates are in each case. I'm assuming there are technical repeats within a single experiment. In fact, there should be documentation comparing between biological repeats between independent experiments.*

Answer: We apologize for omitting details about how the infiltrations were conducted and descriptions of sample sizes and biological replicates used. It took a long time to develop this plant production system for taxanes and over the years the experiments were repeated many times. The data reported here from of three biological repeats (unless mentioned) and independent experiments were repeated at least twice. We took extreme care in standardising OD_{600} before infiltrations and infiltrated leaves were harvested 5 days post infiltration for analyses. We have added details on biological repeats in our experiments in the Methods section (L600, L620) and have now added information about biological replicates and technical replicates on figure legends.

Comment: *The authors cite references which they state provide insight into the importance of compartmentalization for diterpene accumulation in N. benthamiana. When I examine those citations, at least one of the articles did not address diterpene production as suggested by the authors. If the authors cite papers it means they have rigorously read those papers and are citing them accurately. This is critical to the integrity of the scientific community.*

Answer:

We sincerely apologize for this mistake in the wording of the referred sentence (L314 in the first submission). The sentence was citing references on the use of compartmentalisation approaches in production of terpenoids in *N. benthamiana* in previous work, but not necessarily diterpenoids. We have revised the sentence as follows:

"Recently, compartmentalized metabolic engineering in *Nicotiana spp*. has emerged as a promising strategy to overcome some of these problems and improve yields of terpenoids (Fuentes et al., 2016; Malhotra et al., 2016; Wu et al., 2006)." (L400 in the revised manuscript)

Comment: *The authors are also remiss in not mentioning some of the work on transient expression in N. benthamiana for oxidized triterpenes. Wouldn't that work be the precedent for the current work?*

Answer: We thank the reviewer for this suggestion. Yes, indeed, there had been really interesting work on oxidized triterpenes (Reed & Osbourn, 2018; Reed et al., 2017; Stephenson, Reed, Brouwer, & Osbourn, 2018) transient expression in *N. benthamiana* that would help put our work into context. Over the years, work on oxidised triterpenes have really advanced *N. benthamiana* as a powerful expression system for terpenes production in terms of technical resources, purification, scalability and optimizations of protocols, and is very insightful for development of robust and scalable transient production platforms for diterpenes like taxanes presented in our work. We have incorporated work on oxidised triterpenes in the revised manuscript (reference number 38, 55 and 59).

Comment: *In summary, this manuscript represents an important approach to developing production platforms in plants, but the work is preliminary and better suited for a traditionally based specialty journal.*

Answer: We thank the reviewer and we really appreciate these constructive criticisms. There are many advantages to development of a plant production system for engineering the taxol biosynthetic system. In general, the engineering of monoterpenes and sesquiterpenes in plant platforms has advanced significantly while advances in engineering diterpenoids and triterpenoids have not been as successful. In particular, the taxol pathway is one of the most challenging pathways to engineer in chassis organisms due to the complexity of the lengthy pathway and the comparatively large number of cytochrome P450 enzymes involved. While the developed plant platform is not yet as efficient and advanced as the ones in oxidized triterpenes, this work presents a breakthrough in production of taxadiene-5a-ol that has been unsuccessful for a long time, and could lay a solid platform for further development. Another attractive side of a plant production system for taxanes is it could provide a very useful platform for pathway elucidation. Quite a number of steps remain unknown in the long taxol pathway despite a long period of gene and substrate mining using established microbial systems. We have carefully considered all points raised by the reviewer to strengthen the paper and improve both the data and the commentary and we hope this improved clarity on a number of issues. Given the number of technical challenges in engineering taxanes in plant systems, we believe this work will be of interest to the broad readership of this superlatively high-quality journal.

References:

Ajikumar, P. K., Xiao, W.-H., Tyo, K. E. J., Wang, Y., Simeon, F., Leonard, E., … Stephanopoulos, G. (2010). Isoprenoid pathway optimization for Taxol precursor overproduction in Escherichia coli. *Science (New York, N.Y.)*, *330*(6000), 70–74. https://doi.org/10.1126/science.1191652

Barton, N. A., Marsh, B. J., Lewis, W., Narraidoo, N., Seymour, G. B., Fray, R., & Hayes, C. J. (2016). Accessing low-oxidation state taxanes: Is taxadiene-4(5)-epoxide on the taxol biosynthetic pathway? *Chemical Science*, *7*(5), 3102–3107. https://doi.org/10.1039/c5sc03463a

Biggs, B. W., Rouck, J. E., Kambalyal, A., Arnold, W., Lim, C. G., De Mey, M., … Ajikumar, P. K. (2016). Orthogonal Assays Clarify the Oxidative Biochemistry of Taxol P450 CYP725A4. *ACS Chemical Biology*, *11*(5), 1445–1451. https://doi.org/10.1021/acschembio.5b00968

DeJong, J. M., Liu, Y., Bollon, A. P., Long, R. M., Jennewein, S., Williams, D., & Croteau, R. B. (2006). Genetic engineering of taxol biosynthetic genes inSaccharomyces cerevisiae. *Biotechnology and Bioengineering*, *93*(2), 212–224. https://doi.org/10.1002/bit.20694

Ding, M. Z., Yan, H. F., Li, L. F., Zhai, F., Shang, L. Q., Yin, Z., & Yuan, Y. J. (2014). Biosynthesis of taxadiene in saccharomyces cerevisiae: Selection of geranylgeranyl diphosphate synthase directed by a computer-aided docking strategy. *PLoS ONE*, *9*(10). https://doi.org/10.1371/journal.pone.0109348

Engels, B., Dahm, P., & Jennewein, S. (2008). Metabolic engineering of taxadiene biosynthesis in yeast as a first step towards Taxol (Paclitaxel) production. *Metabolic Engineering*, *10*(3–4), 201–206. https://doi.org/10.1016/j.ymben.2008.03.001

Fuentes, P., Zhou, F., Erban, A., Karcher, D., Kopka, J., & Bock, R. (2016). A new synthetic biology approach allows transfer of an entire metabolic pathway from a medicinal plant to a biomass crop. *ELife*, *5*(JUN2016), 1–26. https://doi.org/10.7554/eLife.13664

Gnanasekaran, T., Karcher, D., Nielsen, A. Z., Martens, H. J., Ruf, S., Kroop, X., … Jensen, P. E. (2016). Transfer of the cytochrome P450-dependent dhurrin pathway from Sorghum bicolor into Nicotiana tabacum chloroplasts for light-driven synthesis. *Journal of Experimental Botany*, *67*(8), 2495–2506. https://doi.org/10.1093/jxb/erw067

Ikram, N. K. B. K., & Simonsen, H. T. (2017). A Review of Biotechnological Artemisinin Production in Plants. *Frontiers in Plant Science*, *8*(November), 1–10. https://doi.org/10.3389/fpls.2017.01966

Malhotra, K., Subramaniyan, M., Rawat, K., Kalamuddin, M., Qureshi, M. I., Malhotra, P., … Kumar, S. (2016). Compartmentalized Metabolic Engineering for Artemisinin Biosynthesis and Effective Malaria Treatment by Oral Delivery of Plant Cells. *Molecular Plant*, *9*(11), 1464–1477. https://doi.org/10.1016/j.molp.2016.09.013

Nielsen, A. Z., Ziersen, B., Jensen, K., Lassen, L. M., Olsen, C. E., Møller, B. L., & Jensen, P. E. (2013). Redirecting photosynthetic reducing power toward bioactive natural product synthesis. *ACS Synthetic Biology*, *2*(6), 308–315. https://doi.org/10.1021/sb300128r

Paddon, C. J., Westfall, P. J., Pitera, D. J., Benjamin, K., Fisher, K., McPhee, D., … Newman, J. D. (2013). High-level semi-synthetic production of the potent antimalarial artemisinin. *Nature*, *496*, 528. Retrieved from https://doi.org/10.1038/nature12051

Peplow, M. (2016). Synthetic malaria drug meets market resistance: first commercial deployment of synthetic biology for medicine has modest impact. Nature 530, 389–391. doi: 10.1038/530390a

Reed, J., & Osbourn, A. (2018). Engineering terpenoid production through transient expression in Nicotiana benthamiana. *Plant Cell Reports*, *37*(10), 1431–1441. https://doi.org/10.1007/s00299-018-2296-3

Reed, J., Stephenson, M. J., Miettinen, K., Brouwer, B., Leveau, A., Brett, P., … Osbourn, A. (2017). A translational synthetic biology platform for rapid access to gram-scale quantities of novel drug-like molecules. *Metabolic Engineering*, *42*(July), 185–193. https://doi.org/10.1016/j.ymben.2017.06.012

Rouck, J. E., Biggs, B. W., Kambalyal, A., Arnold, W. R., De Mey, M., Ajikumar, P. K., & Das, A. (2017). Heterologous expression and characterization of plant Taxadiene-5α-Hydroxylase (CYP725A4) in Escherichia coli. *Protein Expression and Purification*, *132*, 60–67. https://doi.org/10.1016/j.pep.2017.01.008

Sagwan-Barkdoll, L., Anterola, A.M. Taxadiene-5α-ol is a minor product of CYP725A4 when expressed in Escherichia coli. Biotechnol. Appl. Bioc. 2017, 5, 294-305.https:// doi.org/10.1002/bab.1606

Stephenson, M. J., Reed, J., Brouwer, B., & Osbourn, A. (2018). Transient Expression in Nicotiana Benthamiana Leaves for Triterpene Production at a Preparative Scale. *Journal of Visualized Experiments*, (138), 1–11. https://doi.org/10.3791/58169

Vogl, T., Kickenweiz, T., Pitzer, J., Sturmberger, L., Weninger, A., Biggs, B. W., … Glieder, A. (n.d.). Engineered bidirectional promoters enable rapid multi-gene co-expression optimization. *Nature Communications*, (2018). https://doi.org/10.1038/s41467-018-05915-w

Wu, S., Schalk, M., Clark, A., Miles, R. B., Coates, R., & Chappell, J. (2006). Redirection of cytosolic or plastidic isoprenoid precursors elevates terpene production in plants. *Nature Biotechnology*, *24*(11), 1441–1447. https://doi.org/10.1038/nbt1251

Zhou, K., Qiao, K., Edgar, S., & Stephanopoulos, G. (2015). Distributing a metabolic pathway among a microbial consortium enhances production of natural products. *Nature Biotechnology*, *33*(4), 377–383. https://doi.org/10.1038/nbt.3095

Reviewers' comments:

Reviewer #1 unavailable.

Reviewer #2 (Remarks to the Author):

Reviewer comments have been mostly addressed. The work comprising Figure S17 is an appreciated addition to the manuscript.
Previously there has been several groups that have successfully demonstrated the production of taxadiene in several different systems (plants and microbial) and have provided the NMR and C13-NMR. One of the innovation in this work is the next step of the synthesis of the taxane-5-ol. The GC-MS is not enough for characterization of the product. Even a proton NMR can be sufficient which requires 0.5 mg to 1mg of the compound or using a longer acquisition time. The reported yield in the paper is the following.
“After co-expression with TS, there was an increase in the amount of taxadiene to 9.9 μg/g FW (about 2.0-fold higher than engineering in the cytoplasm), and a remarkable increase in taxadiene-5ɑ-ol (Supplementary Fig. S2) to 0.90 μg/g FW (almost 4.3-fold increase as compared to 228 yields from cytoplasmic engineering) in tobacco leaves”
If the reported yield is ~1 ug/g of leaves. The author will require 500g of leaves to get enough material to run a characterization. Despite the OCT being unstable and forming iso-OCT, it is worth to purify the downstream compounds for 1-NMR analysis even though C-13 NMR analysis is not possible.
As the focus of the paper is bioengineering a plant to produce high quantity of both taxadiene and taxadiene-5-alpha-ol, characterization of the products in important as any of the other hydroxylated products can be potentially formed.

Figures could still use some modification to improve their clarity. For example quality of Figure 5a is very poor, difficult to read and the figures should be remade. On Figure 5a, the rightmost subpanel has no header, unlike the other two subpanels within panel 5a.

Figure 6, for example: the colored print on panel 6a has a strange discoloration/shadow. Please remove this from the figure as it is distracting. These types of colorations make it difficult to distinguish the chemical structure from the rest of the schematic. Please change all chemical structure line settings to a line width of 0.0183 inches and bold all heteroatoms in all structures.

The manuscript reads better than the previous draft and provides a more cohesive narrative to recent research progress. In addition to the points made by the authors concerning the importance of this work, it would be worthwhile to emphasize the difficulty in working with non-model plant organisms. Optimization of experimental procedures and growth conditions, as well as lack of sustainability and lifetime differences among non-model plants, are details, which researchers outside of plant specialty fields do not take into consideration.
To supplement the advantages provided in the text concerning the use of heterologous plant host systems, it should be emphasized how more efforts found in this manuscript must be pursued so that bottlenecks surrounding non-model plant systems can be circumvented and plant metabolic engineering efforts can progress and compete against microbe-based efforts.

Reviewer #3 (Remarks to the Author):

The authors have made a valent attempt to address my earlier concerns. However, my overall sentiment remains as before. The work is good, but not the conceptual leap forward the authors contend. All the experimental details still don’t fall together to give a cogent picture. I tried to be subtle in my earlier review suggesting that the authors might not have captured the conceptual

underpinnings of work proceeding their work, but can be more explicit now. But first, I will elaborate below on some of the technical issues related to the data.

Figure 2a depicting the immunoblot for TS expression exhibits multiple bands. Why? Slow processing of the protein in vivo? Assuming the larger band in the anti-flag blot corresponds to unprocess TS (n-terminal targeting signal sequence still retained), this raises the issue if the unprocessed protein in catalytically active. Is it? In comparison to n-terminal cleaved protein, how active? It is hard to see if the T5aH and CPR are also processed. Is there any evidence that either or both are actually n-terminal cleavage products? Are these catalytically active?

The terminology "Recombinant protein expression and committed taxadiene production …" are inappropriate. Figure 2 shows the accumulation of the TS, P450 and Reductase proteins in plants expressing the corresponding cDNA constructs (a). Panels b and c show the corresponding amounts of taxadiene accumulating in leaves after agroinfiltration with the TS construct (with the epitope-tag?).

Figure 3 is to convince readers that the proteins are processed and inserted into the chloroplast as defined by their targeting signal sequences. Okay, so based on the immunoblots in Fig. 2, I would expect about 50% of the tpTS tagged protein to get into the chloroplast and 50% not. What is evident in the TS-GFP panel is very intense punctate staining. When overlayed with the autofluoresence image, much of the GFP appears to be co-localized to the plastid compartment. But what about the other 50% of the TS protein that doesn't seem to be processed under these conditions (in Fig. 2)? Is it simply imported but not cleaved? Are both molecular species catalytically active?

Figure 4 depicts the taxane accumulation profiles for the 3 proteins targeted to the cytosol and plastid, and there is unexpected results here as well. When TS is targeted to the plastid, yet the P450 and reductase are directed to the cytosol, taxane accumulation is ~ 4 µg/g FW and oxidized taxane is about 1. When all three enzymes are directed to the cytosol, taxane level is boosted a bit as is the oxidized form. Then, when all three are targeted to the plastid, both the taxane and oxidized forms double in amounts. Why is this the case? Why doesn't targeting of the TS to the plastid itself able to yield taxane levels when all three proteins are targeted to the plastid?

Figure 5 provides evidence that over-expression of the DXS in combination with GGPS and TS boost taxane levels 10-fold. This is significant and important, but the precedence for this was well established by others engineering mono- and sesqui-terpene production into the plastids. Hence, the result is expected.

The final piece of evidence provided is when DXS, GGPS, TS, P450 and Reductase are all targeted to the plastid and the taxane profiles measured. Quite surprising here, the taxane level is reduced to about one-quarter of that when just targeting the TS protein to the plastid. But then, one might imagine that the level of oxidized products were correspondingly increased. But that too isn't quite the case. The level is marginally increased above that when the TS, P450 and Reductase are all targeted to the plastid (Fig. 4). So, if the results are as reproducible as the authors suggest in the rebuttal letter, then more than two-thirds of the diterpene hydrocarbon remains unaccounted for in the experiment depicted in Fig. 6.

When I pointed out previously the prior research articles cited didn't necessarily focus on diterpenes, I had hoped the authors would appreciate that this reviewer examined those papers closely as well. Perhaps I should have been more explicit. What is uncanny in the current manuscript is how the work in this manuscript follows upon that described by Wu et al. Including how figures are constructed and the strategies used to unravel the contribution of the MVA and MEP pathway to terpene metabolism installed in the plastid versus cytosol. True, that work focused on another class of terpenes, but the approaches and strategies employed in the current work are almost identical, yet portrayed as if originating with the current authors.

What is equally important to note, the Wu et al. work occurred long ago and there have been many additional efforts to install terpene synthases and decorating enzymes into the chloroplast to effect complex biosynthesis. Maybe the authors are unaware of these efforts. But it is not justified to portray the current work without due reference to the conceptual frontier provided by the work of others. The current work is good and important, but it is neither ground breaking nor precedent setting. It is the logical progression of solid scientific progress.

Lastly, the rebuttal letter and the revision leads to more questions than answers. For instance, the unknown "putative" triterpene that with mass fragments of 208 and 213. I'm not able to follow this logic. Such ions will be evident in sesqui-, di-, tri- and tetra-terpenes. This is just an example of the confusion created by the rebuttal letter - there are many others.

Reviewer #4 (Remarks to the Author):

The present work set up a metabolic pathway in the plant Nicotiana benthamiana for producing the precursors of Taxol. The authors demonstrated that the plant platform produced not only taxadiene but also taxadiene-5ɑ-ol and the latter is the first report to be produced by the plant chasis. They also manifested that these precursors were mainly produced by the MEP pathway. The manuscript is meaningful for developing production platform in plants. The authors have basically answered the questions involved in the comment of the reviewer #1. And the manuscript has been substantially revised following the reviewers' suggestions, including supplementing some new data.
In the past decade, some outstanding achievements have been made by other research groups. For example, as mentioned by the authors, Ajikumar, et al. achieved ~1 g taxadiene/L and ~60 mg taxadiene-5ɑ-ol/L, respectively, in an engineered E. coli strain [Ajikumar, et al. (2010) Isoprenoid pathway optimization for Taxol precursor overproduction in Escherichia coli, Science 330, 70-74]. This group also achieved ~570 mg oxygenated taxanes/L in E. coli [Biggs, et al.(2016) Overcoming heterologous protein interdependency to optimize P450-mediated Taxol precursor synthesis in Escherichia coli. Proc. Natl. Acad. Sci. 113, 3209–3214.].These represent the highest yields of the two taxanes produced by the metabolic engineering method. However, the two precursors are far from Taxol and even far from the two important intermediates 10-deacetylbaccatin III and baccatin III. Therefore, if the work to engineer the Taxol pathway into a heterologous host advances beyond the two precursors taxadiene and taxadiene-5ɑ-ol, it will be more compelling. Of course it is an arduous job.
I suggest the authors to reorganize the introduction to address these achievements in the yields, and in the discussion section, to compare the present yields with those previously reports.
Other comments can be found in the annotations of the manuscript.

Reviewers' comments:

Reviewer #1 (unavailable):

Reviewer #2 (Remarks to the Author):

Reviewer comments have been mostly addressed. The work comprising Figure S17 is an appreciated addition to the manuscript.
Previously there has been several groups that have successfully demonstrated the production of taxadiene in several different systems (plants and microbial) and have provided the NMR and C13-NMR. One of the innovation in this work is the next step of the synthesis of the taxane-5-ol. The GC-MS is not enough for characterization of the product. Even a proton NMR can be sufficient which requires 0.5 mg to 1mg of the compound or using a longer acquisition time. The reported yield in the paper is the following.
"After co-expression with TS, there was an increase in the amount of taxadiene to 9.9 μg/g FW (about 2.0-fold higher than engineering in the cytoplasm), and a remarkable increase in taxadiene-5α-ol (Supplementary Fig. S2) to 0.90 μg/g FW (almost 4.3-fold increase as compared to 228 yields from cytoplasmic engineering) in tobacco leaves"
If the reported yield is ~1 ug/g of leaves. The author will require 500g of leaves to get enough material to run a characterization. Despite the OCT being unstable and forming iso-OCT, it is worth to purify the downstream compounds for 1-NMR analysis even though C-13 NMR analysis is not possible.
As the focus of the paper is bioengineering a plant to produce high quantity of both taxadiene and taxadiene-5-alpha-ol, characterization of the products in important as any of the other hydroxylated products can be potentially formed.

Figures could still use some modification to improve their clarity. For example quality of Figure 5a is very poor, difficult to read and the figures should be remade. On Figure 5a, the rightmost subpanel has no header, unlike the other two subpanels within panel 5a.

Figure 6, for example: the colored print on panel 6a has a strange discoloration/shadow. Please remove this from the figure as it is distracting. These types of colorations make it difficult to distinguish the chemical structure from the rest of the schematic. Please change all chemical structure line settings to a line width of 0.0183 inches and bold all heteroatoms in all structures.

The manuscript reads better than the previous draft and provides a more cohesive narrative to recent research progress. In addition to the points made by the authors concerning the importance of this work, it would be worthwhile to emphasize the difficulty in working with non-model plant organisms.
Optimization of experimental procedures and growth conditions, as well as lack of sustainability and lifetime differences among non-model plants, are details, which researchers outside of plant specialty fields do not take into consideration.
To supplement the advantages provided in the text concerning the use of heterologous plant host systems, it should be emphasized how more efforts found in this manuscript must be pursued so that bottlenecks surrounding non-model plant systems can be circumvented and plant metabolic engineering efforts can progress and compete against microbe-based efforts.

Reviewer #3 (Remarks to the Author):

The authors have made a valent attempt to address my earlier concerns. However, my overall sentiment remains as before. The work is good, but not the conceptual leap forward the authors contend. All the experimental details still don't fall together to give a cogent picture. I tried to be subtle in my earlier review suggesting that the authors might not have captured the conceptual underpinnings of work proceeding their work, but can be more explicit now. But first, I will elaborate below on some of the technical issues related to the data.

Figure 2a depicting the immunoblot for TS expression exhibits multiple bands. Why? Slow processing of the protein in vivo? Assuming the larger band in the anti-flag blot corresponds to unprocess TS (n-terminal targeting signal sequence still retained), this raises the issue if the unprocessed protein in catalytically active. Is it? In comparison to n-terminal cleaved protein, how active? It is hard to see if the T5aH and CPR are also processed. Is there any evidence that either or both are actually n-terminal cleavage products? Are these catalytically active?

The terminology "Recombinant protein expression and committed taxadiene production …" are inappropriate. Figure 2 shows the accumulation of the TS, P450 and Reductase proteins in plants expressing the corresponding cDNA constructs (a). Panels b and c show the corresponding amounts of taxadiene accumulating in leaves after agroinfiltration with the TS construct (with the epitope-tag?).

Figure 3 is to convince readers that the proteins are processed and inserted into the chloroplast as defined by their targeting signal sequences. Okay, so based on the immunoblots in Fig. 2, I would expect about 50% of the tpTS tagged protein to get into the chloroplast and 50% not. What is evident in the TS-GFP panel is very intense punctate staining. When overlayed with the autofluoresence image, much of the GFP appears to be co-localized to the plastid compartment. But what about the other 50% of the TS protein that doesn't seem to be processed under these conditions (in Fig. 2)? Is it simply imported but not cleaved? Are both molecular species catalytically active?

Figure 4 depicts the taxane accumulation profiles for the 3 proteins targeted to the cytosol and plastid, and there is unexpected results here as well. When TS is targeted to the plastid, yet the P450 and reductase are directed to the cytosol, taxane accumulation is ~ 4 µg/g FW and oxidized taxane is about 1. When all three enzymes are directed to the cytosol, taxane level is boosted a bit as is the oxidized form. Then, when all three are targeted to the plastid, both the taxane and oxidized forms double in amounts. Why is this the case? Why doesn't targeting of the TS to the plastid itself able to yield taxane levels when all three proteins are targeted to the plastid?

Figure 5 provides evidence that over-expression of the DXS in combination with GGPS and TS boost taxane levels 10-fold. This is significant and important, but the precedence for this was well established by others engineering mono- and sesqui-terpene production into the plastids. Hence, the result is expected.

The final piece of evidence provided is when DXS, GGPS, TS, P450 and Reductase are all targeted to the plastid and the taxane profiles measured. Quite surprising here, the taxane level is reduced to about one-quarter of that when just targeting the TS protein to the plastid. But then, one might imagine that the level of oxidized products were correspondingly increased. But that too isn't quite the case. The level is marginally increased above that when the TS, P450 and Reductase are all targeted to the plastid (Fig. 4). So, if the results are as reproducible as the authors suggest in the rebuttal letter, then more than two-thirds of the

diterpene hydrocarbon remains unaccounted for in the experiment depicted in Fig. 6.

When I pointed out previously the prior research articles cited didn’t necessarily focus on diterpenes, I had hoped the authors would appreciate that this reviewer examined those papers closely as well. Perhaps I should have been more explicit. What is uncanny in the current manuscript is how the work in this manuscript follows upon that described by Wu et al. Including how figures are constructed and the strategies used to unravel the contribution of the MVA and MEP pathway to terpene metabolism installed in the plastid versus cytosol. True, that work focused on another class of terpenes, but the approaches and strategies employed in the current work are almost identical, yet portrayed as if originating with the current authors.

What is equally important to note, the Wu et al. work occurred long ago and there have been many additional efforts to install terpene synthases and decorating enzymes into the chloroplast to effect complex biosynthesis. Maybe the authors are unaware of these efforts. But it is not justified to portray the current work without due reference to the conceptual frontier provided by the work of others. The current work is good and important, but it is neither ground breaking nor precedent setting. It is the logical progression of solid scientific progress.

Lastly, the rebuttal letter and the revision leads to more questions than answers. For instance, the unknown “putative” triterpene that with mass fragments of 208 and 213. I’m not able to follow this logic. Such ions will be evident in sesqui-, di-, tri- and tetra-terpenes. This is just an example of the confusion created by the rebuttal letter - there are many others.

Reviewer #4 (Remarks to the Author):

The present work set up a metabolic pathway in the plant Nicotiana benthamiana for producing the precursors of Taxol. The authors demonstrated that the plant platform produced not only taxadiene but also taxadiene-5α-ol and the latter is the first report to be produced by the plant chasis. They also manifested that these precursors were mainly produced by the MEP pathway. The manuscript is meaningful for developing production platform in plants. The authors have basically answered the questions involved in the comment of the reviewer #1. And the manuscript has been substantially revised following the reviewers’ suggestions, including supplementing some new data.

In the past decade, some outstanding achievements have been made by other research groups. For example, as mentioned by the authors, Ajikumar, et al. achieved ~1 g taxadiene/L and ~60 mg taxadiene-5α-ol/L, respectively, in an engineered E. coli strain [Ajikumar, et al. (2010) Isoprenoid pathway optimization for Taxol precursor overproduction in Escherichia coli, Science 330, 70-74]. This group also achieved ~570 mg oxygenated taxanes/L in E. coli [Biggs, et al.(2016) Overcoming heterologous protein interdependency to optimize P450-mediated Taxol precursor synthesis in Escherichia coli. Proc. Natl. Acad. Sci. 113, 3209–3214.].These represent the highest yields of the two taxanes produced by the metabolic engineering method. However, the two precursors are far from Taxol and even far from the two important intermediates 10-deacetylbaccatin III and baccatin III. Therefore, if the work to engineer the Taxol pathway into a heterologous host advances beyond the two precursors taxadiene and taxadiene-5α-ol, it will be more compelling. Of course it is an arduous job.

I suggest the authors to reorganize the introduction to address these achievements in the yields, and in the discussion section, to compare the present yields with those previously reports.

Other comments can be found in the annotations of the manuscript.

Point-by-point response to reviewers' comments:

General Response

We would like to thank all the reviewers for their thorough evaluation of the manuscript and for the many constructive comments and suggestions. We have carefully considered all the comments and revised the manuscript accordingly. Detailed responses to the reviewers' comments are included below. Revised areas in the main manuscript and the supplementary data are in blue.

Reviewer #1 (unavailable):

Reviewer #2 (Remarks to the Author):

Comment: *Previously there has been several groups that have successfully demonstrated the production of taxadiene in several different systems (plants and microbial) and have provided the NMR and C13-NMR. One of the innovation in this work is the next step of the synthesis of the taxane-5-ol. The GC-MS is not enough for characterization of the product. Even a proton NMR can be sufficient which requires 0.5 mg to 1mg of the compound or using a longer acquisition time. The reported yield in the paper is the following.*

"After co-expression with TS, there was an increase in the amount of taxadiene to 9.9 μg/g FW (about 2.0-fold higher than engineering in the cytoplasm), and a remarkable increase in taxadiene-5 α-ol (Supplementary Fig. S2) to 0.90 μg/g FW (almost 4.3-fold increase as compared to yields from cytoplasmic engineering) in tobacco leaves"

If the reported yield is ~1 ug/g of leaves. The author will require 500g of leaves to get enough material to run a characterization. Despite the OCT being unstable and forming iso-OCT, it is worth to purify the downstream compounds for 1-NMR analysis even though C-13 NMR analysis is not possible.

As the focus of the paper is bioengineering a plant to produce high quantity of both taxadiene and taxadiene-5-alpha-ol, characterization of the products in important as any of the other hydroxylated products can be potentially formed.

Answer: We completely agree that NMR data of oxidized taxanes would strengthen the result of this contribution. As suggested by the reviewer, we have performed more production and elaborate purification of oxygenated taxanes from our engineered tobacco system using the DXS-GGPPS and TS-tp(TS)/trT5H/trCPR co-expressing leaves (see details added in supplementary material). However, this was not a trivial task due to the complex background of the plant tissue. We achieved high purity of each desired compound by GC-MS analysis, the new MS data has now been added as Supplementary Fig. S3, S4, S6, and S8. These data provide identity of the focal compounds, and the retention time of taxa-4,(5)-11(12)-diene(**1**), iso-OCT (**4**), OCT (**3**), and taxadiene-5α-ol (**2**) were 18.8 min, 19.5 min, 20.2min, and 20.6 min, respectively, agreeing with the peaks in MS spectra from our previous analyses. For a comparison of TIC and mass spectra of purified taxanes from transient plant tissue, see Figures R1-R4.

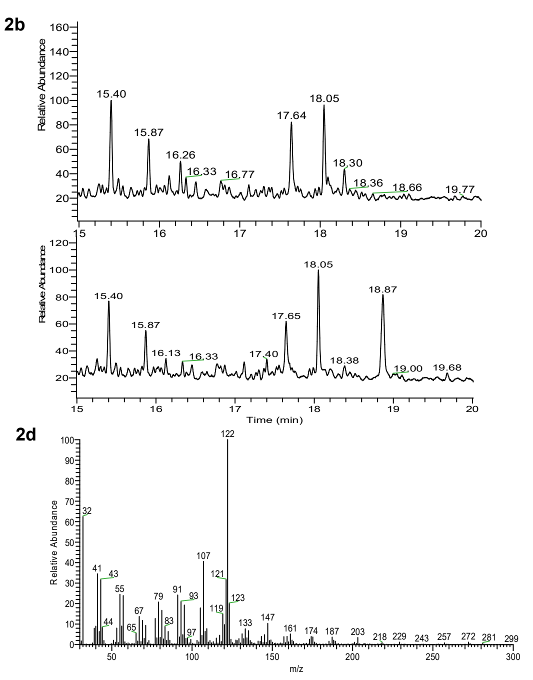

Fig2b, 2d. GC-MS analysis of committed taxadiene in transient tobacco and mass spectrum of taxa-4(5),11(12)-diene (**1**).

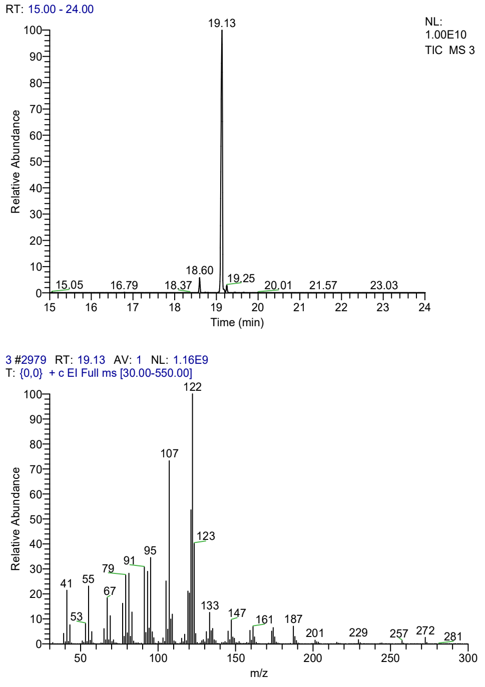

Figure S3. GC-MS analysis of purified taxa-4(5),11(12)-diene (**1**). Top panel shows the TIC of purified **1**. The peak at retention time 19.1 min corresponds to **1**. The lower panel shows the mass spectrum of **1**.

Figure R1. Comparison of TIC and mass spectra of taxa-4(4),11(12)-diene (**1**) (tR=18.8 min) produced by transient *N. benthamiana* with purified **1** from transient plant tissue. The retention time of **1** was delayed by 0.3 min due to column regeneration.

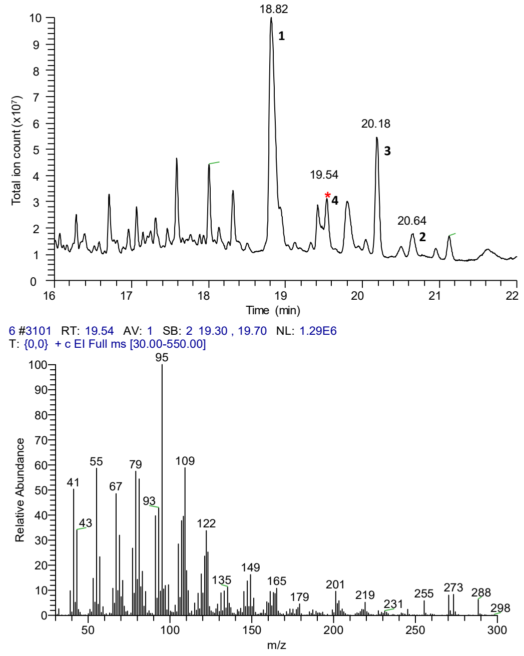

Figure S7. GC-MS analysis of 5(13)-oxa-3(11)-cyclotaxane (iso-OCT). The Top panel shows the total ion chromatogram (TIC) of hexane/ ethyl acetate (4:1) extracts from DXS- GGPPS and TS-tp(TS)/trT5H/trCPR co-transient *N. benthamiana* leaves. The peak at retention time 19.5 min corresponds to iso-OCT (**4**). The bottom panel shows the mass spectrum of iso-cyclotaxane **4**.

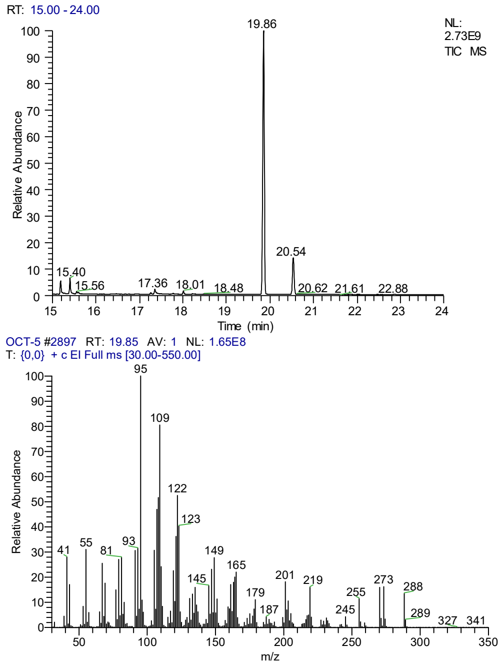

Figure S8. GC-MS analysis of purified iso-OCT. Top panel shows the TIC of purified iso-OCT and the peak at retention time 19.8 min corresponds to desired compound. The lower panel shows the mass spectrum of **4**.

Figure R2. Comparison of TIC and mass spectra of iso-OCT (**4**) (tR=19.5 min) produced by transient *N. benthamiana* with purified **4** from transient plant tissue. The retention time of **4** was delayed by 0.3 min due to column regeneration.

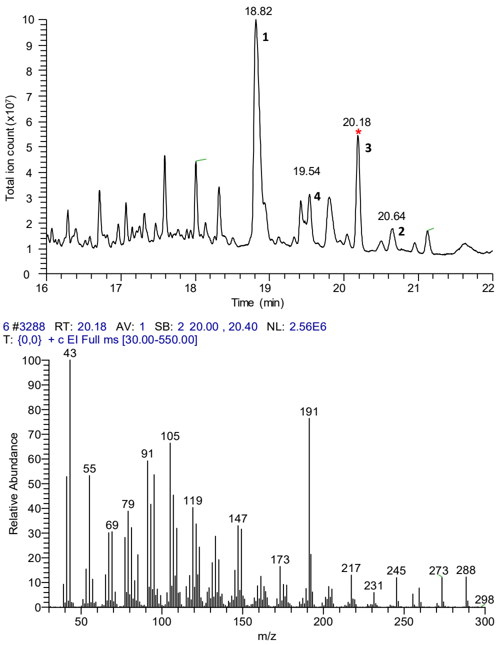

Figure S5. GC-MS analysis of 5(12)-oxa-3(11)-cyclotaxane (OCT). Top panel shows the total ion chromatogram (TIC) of hexane/ ethyl acetate (4:1) extracts from DXS- GGPPS and TS-tp(TS)/trT5H/trCPR co-transient *N. benthamiana* leaves. The peak at retention time 20.2 min corresponds to cyclic-cyclotaxane (OCT, **3**). The lower panel shows the mass spectrum of **3**.

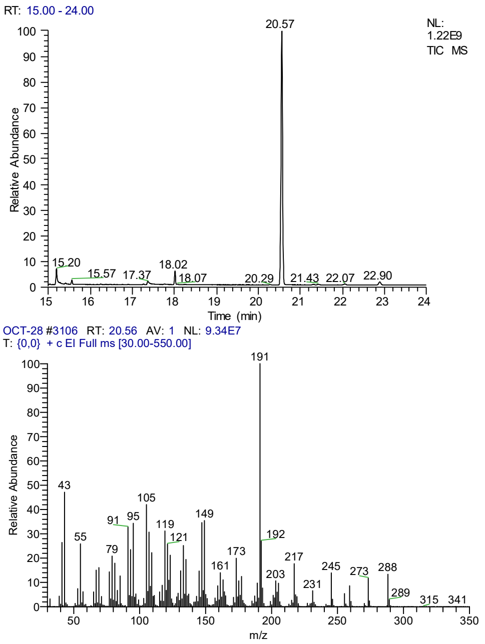

Figure S6. GC-MS analysis of purified OCT. Top panel shows the TIC of purified OCT and the peak at retention time 20.5 min corresponds to desired compound. The lower panel shows the mass spectrum of **3**.

Figure R3. Comparison of TIC and mass spectra of OCT (5) (tR=20.2 min) produced by transient *N. benthamiana* with purified 5 from transient plant tissue. The retention time of 5 was delayed by 0.4 min due to column regeneration.

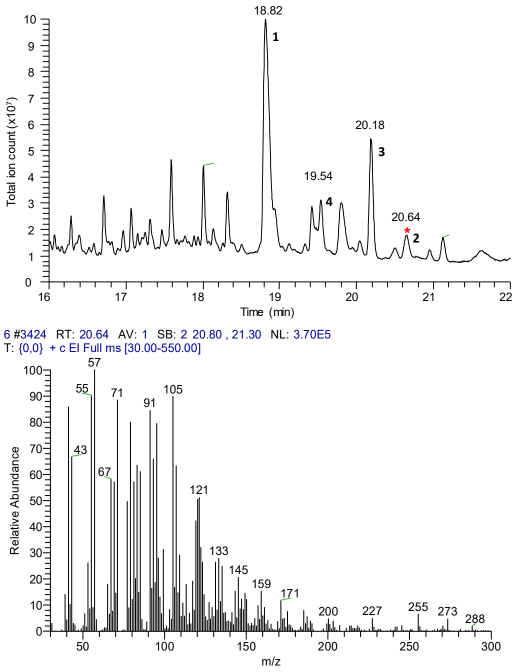

Figure S2. GC-MS analysis of taxadiene-5α-ol. Top panel shows the total ion chromatogram (TIC) of hexane/ ethyl acetate (4:1) extracts from DXS- GGPPS and TS-tp(TS)/trT5H/trCPR co-transient *N. benthamiana* leaves. The peak at retention time 20.6 min corresponds to taxadiene-5α-ol (**2**). The lower panel shows the mass spectrum of **2**.

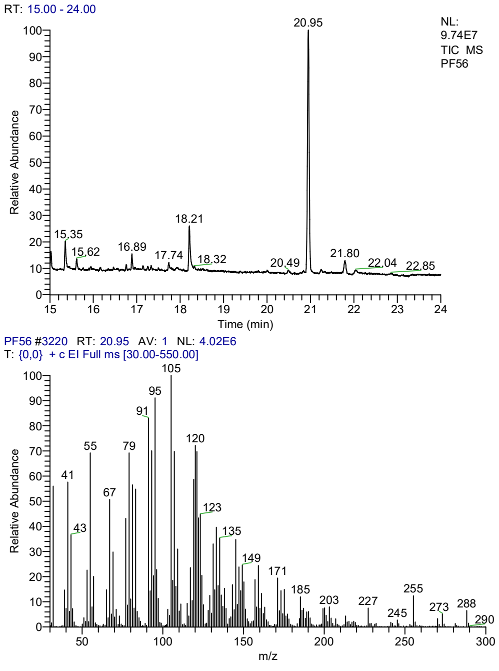

Figure S4. GC-MS analysis of purified taxadiene-5α-ol (**2**). Top panel shows the TIC of purified **2**. The peak at retention time 20.9 min corresponds to desired compound. The lower panel shows the mass spectrum of **2**.

Figure R4. Comparison of TIC and mass spectra of taxadiene-5α-ol (**2**) (tR=20.6 min) produced by transiently expressed *N. benthamiana* with the purified **2** from transient plant tissue. The retention time of 2 was delayed by 0.3 min due to column regeneration.

While we achieved chromatographically high pure compounds, however, the ^{1}H-NMR spectra of iso-OCT and taxadiene-5α-ol retained little traces of impurity peaks despite several and extensive attempts of purification. The ^{1}H-NMR of Iso-OCT gave three impurity signals at δ7.53 ppm, 7.35 ppm and 7.11 ppm (Figure S17).

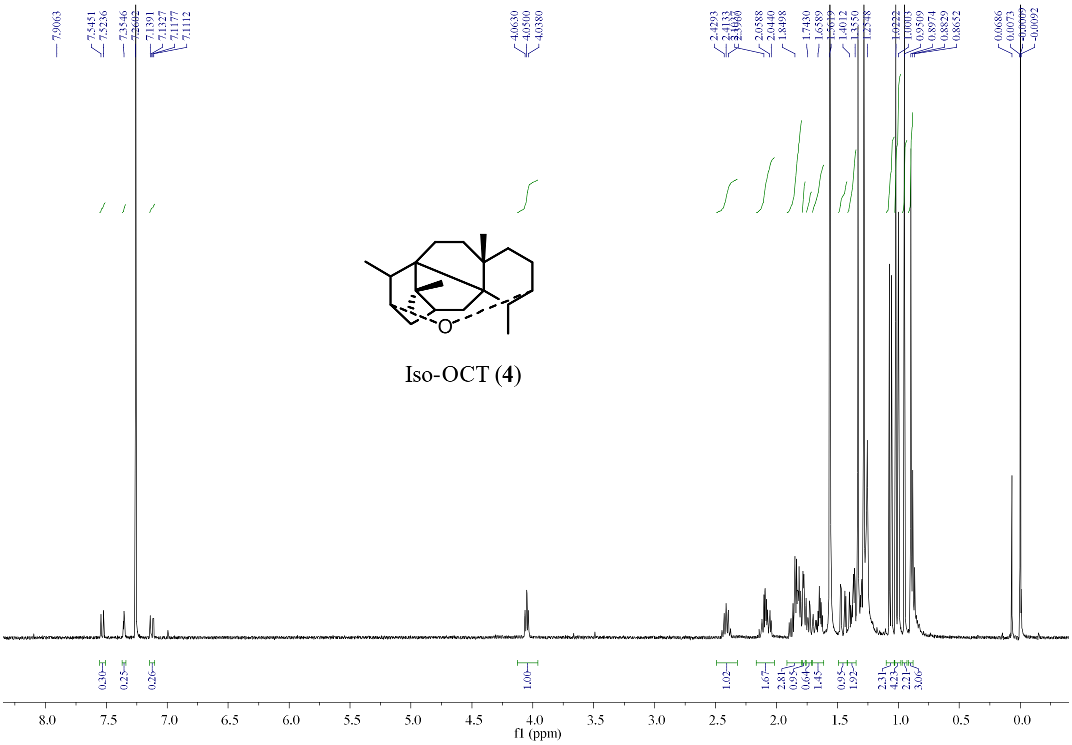

Figure S17. ^{1}H-NMR spectrum of purified iso-OCT (**4**) from transient plant tissues

The ^{1}H-NMR spectrum of purified OCT showed a mixture of OCT and iso-OCT due to structural instability of OCT. The same impurity can also be found in the spectrum (Figure S19).

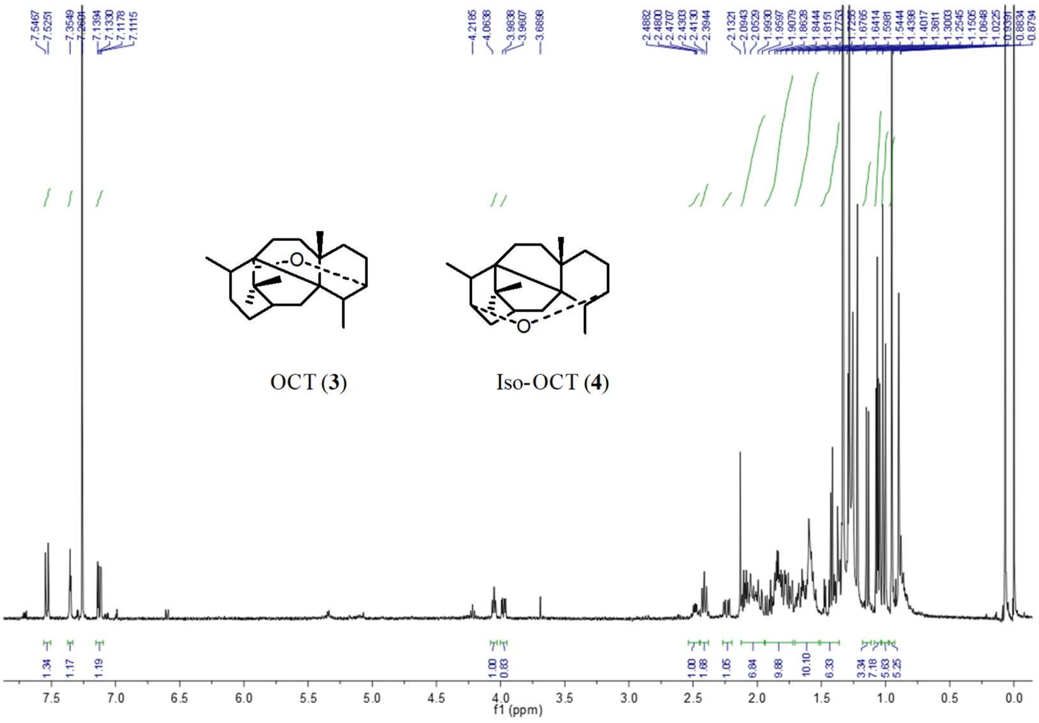

Figure S19. ^{1}H-NMR spectrum of purified OCT (**3**) from transient plant tissues

The ¹H-NMR of taxadiene-5α-ol gave three impurity signals at δ7.71 ppm, 7.54 ppm and 4.31 ppm (Figure S21).

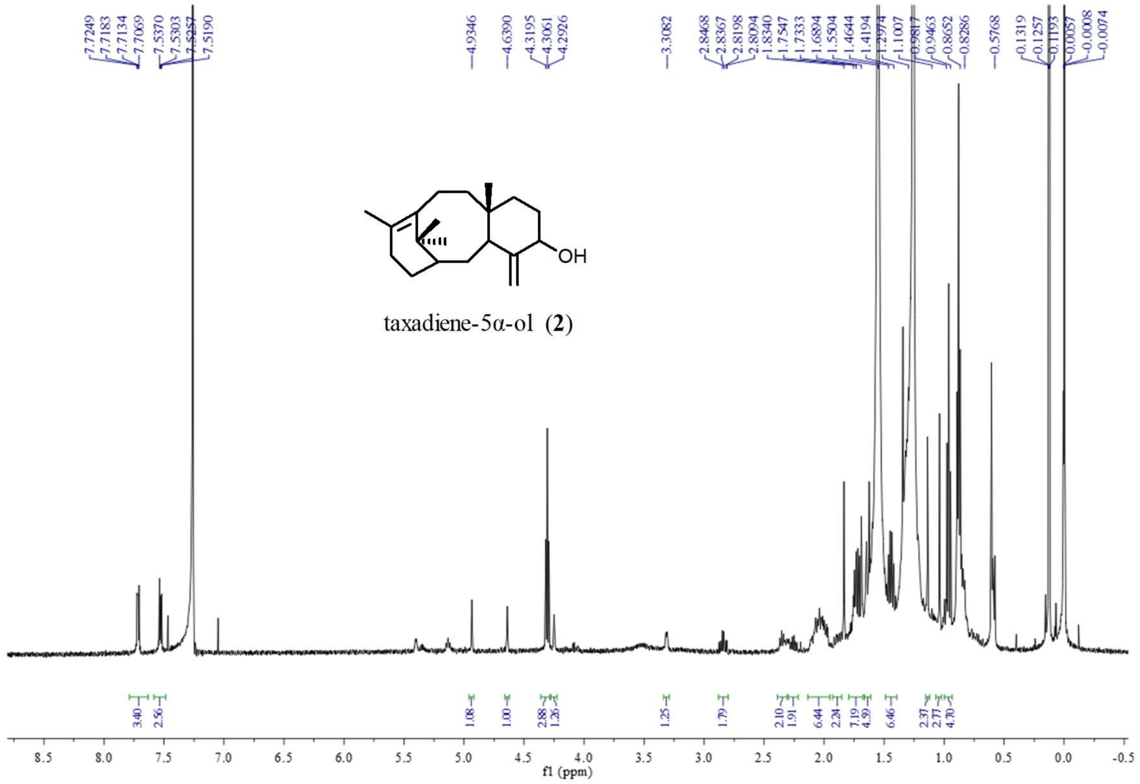

Figure S21. ¹H-NMR spectrum of purified taxadiene-5a-ol (**2**) from transient plant tissues

Comment: *Figures could still use some modification to improve their clarity. For example quality of Figure 5a is very poor, difficult to read and the figures should be remade. On Figure 5a, the rightmost subpanel has no header, unlike the other two subpanels within panel 5a.*
Figure 6, for example: the colored print on panel 6a has a strange discoloration/shadow. Please remove this from the figure as it is distracting. These types of colorations make it difficult to distinguish the chemical structure from the rest of the schematic. Please change all chemical structure line settings to a line width of 0.0183 inches and bold all heteroatoms in all structures.

Answer: Thanks for the comments. We have remade and improved the quality of all figures, including chemical structures.

Comment: *The manuscript reads better than the previous draft and provides a more cohesive narrative to recent research progress. In addition to the points made by the authors concerning the importance of this work, it would be worthwhile to emphasize the difficulty in working with non-model plant organisms.*
Optimization of experimental procedures and growth conditions, as well as lack of sustainability and lifetime differences among non-model plants, are details, which researchers outside of plant specialty fields do not take into consideration.
To supplement the advantages provided in the text concerning the use of heterologous plant host systems, it should be emphasized how more efforts found in this manuscript must be pursued so that bottlenecks surrounding non-model plant systems can be circumvented and plant metabolic engineering efforts can progress and compete against microbe-based efforts.

Answer: We thank the reviewer for positive evaluation of our work and for these excellent suggestions on improving the discussion. In general, plant-based systems are challenging in terms of genetic manipulation, optimization of engineered pathways, complexities in their biological background, tendency for undesirable downstream reactions and optimization of growth parameters of transgenic plants. Non-model plants are even more challenging due to lack of genetic tools, so in this work, we chose *Nicotiana benthamiana* as a host system for engineering taxanes. While we haven`t extended the discussion to non-model plants, we have added statements in the discussion to highlight the points raised by the reviewer (L498-L500, L512).

Reviewer #3 (Remarks to the Author):

Comment:*The authors have made a valent attempt to address my earlier concerns. However, my overall sentiment remains as before. The work is good, but not the conceptual leap forward the authors contend. All the experimental details still don't fall together to give a cogent picture. I tried to be subtle in my earlier review suggesting that the authors might not have captured the conceptual underpinnings of work proceeding their work, but can be more explicit now. But first, I will elaborate below on some of the technical issues related to the data.*

Answer: We appreciate the reviewer for raising these constructive criticisms, and have made efforts to address them as stated below.

Comment:*Figure 2a depicting the immunoblot for TS expression exhibits multiple bands. Why? Slow processing of the protein in vivo? Assuming the larger band in the anti-flag blot corresponds to unprocess TS (n-terminal targeting signal sequence still retained), this raises the issue if the unprocessed protein in catalytically active. Is it? In comparison to n-terminal cleaved protein, how active? It is hard to see if the T5aH and CPR are also processed. Is there any evidence that either or both are actually n-terminal cleavage products? Are these catalytically active?*

Answer: The reviewer raises a very valuable and interesting point on heterologous protein processing in plant hosts. Indeed, native TS is translated as a preprotein bearing N-terminal targeting sequences for localization to plastids. And as evident from fluorescence labelling in Fig. 3a, the protein completely localizes in the chloroplast in tobacco plants, as expected. That the bands reflect slow processing of the protein in tobacco plant cells is a possibility we also considered. To investigate further if the multiple bands are a manifestation of existence of TS with different versions of the signal peptide in tobacco cells, we have carried out an additional experiment with western blot of truncated TS (N terminal 58 amino acid removed). The new result showed only single bands (Figure R5, below) indicating that the multiple bands in Fig 3a may represent different version of the signal peptide. The result also confirms that TS processing exists in planta, as has been previously mentioned by the Croteau group [Williams, et al. (2000) Heterologous expression and characterization of a "pseudomature" form of taxadiene synthase involved in Paclitaxel (Taxol) biosynthesis and evaluation of a potential intermediate and inhibitors of the multistep diterpene cyclization reaction, Arch Biochem Biophys, 379, 137-146].

It is hard to determine the catalytic activity of the different versions of TS in plant cells. However, in the work mentioned above (Williams et al., 2000), a series of full length and truncated forms of TS were expressed and investigated on their kinetic properties, solubility, and stability in *E. coli*. Results demonstrated the full length preprotein and two truncated versions (60 and 79 amino acids truncated) were demonstrated to be catalytically active. In this study using a plant chassis, it is hard to distinguish the activity of full length and truncated protein variants after expressing the full-length version, since plants possess a native signal processing machinery. However, expression of a truncated TS (cytoplasmic engineering, Fig. 4 middle panel) clearly shows that it is active, but it is impossible to measure activity of a full-length TS in parallel for direct comparison.

Regarding T5αH and CPR, the native enzymes exhibited single bands (Fig. 2a), and since they are cytoplasmic proteins, N-truncation is expected in Fig 2a. However, the engineered protein

tp(TS)-trT5H/trCPR which is fused with the N terminal of TS in chloroplastic engineering might also be processed in a similar manner, resulting in different version of T5αH/CPR forms. On another note, post-translational processing could present a possible target for future optimization of our system.

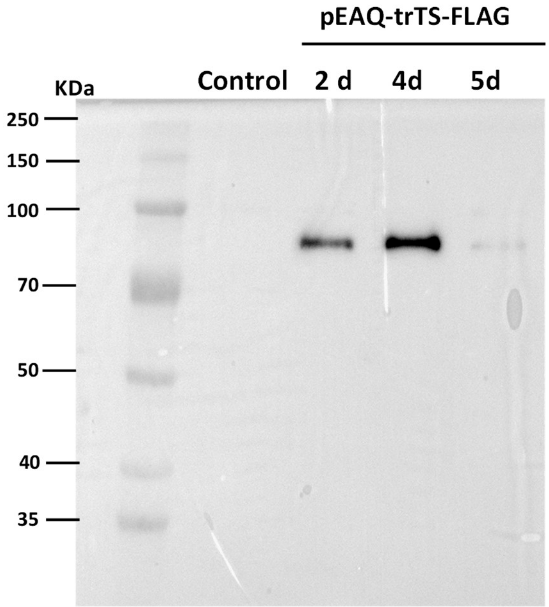

Figure R5. Western blotting analysis of truncated tr58TS

<u>Comment:</u> *The terminology "Recombinant protein expression and committed taxadiene production …" are inappropriate. Figure 2 shows the accumulation of the TS, P450 and Reductase proteins in plants expressing the corresponding cDNA constructs (a). Panels b and c show the corresponding amounts of taxadiene accumulating in leaves after agroinfiltration with the TS construct (with the epitope-tag?).*

<u>Answer:</u> The Figure legend has been revised. As is the case with all the other figure legends in the manuscript, the legend starts with an overall description (expression of heterologous proteins and accumulation of taxadiene), followed by the specifics a, b, c., etc. For metabolite production, we re-constructed and used the sequences without any epitope-tag.

<u>Comment</u>: *Figure 3 is to convince readers that the proteins are processed and inserted into the chloroplast as defined by their targeting signal sequences. Okay, so based on the immunoblots in Fig. 2, I would expect about 50% of the tpTS tagged protein to get into the chloroplast and 50% not. What is evident in the TS-GFP panel is very intense punctate staining. When overlayed with the autofluoresence image, much of the GFP appears to be co-localized to the plastid compartment. But what about the other 50% of the TS protein that doesn't seem to be processed under these conditions (in Fig. 2)? Is it simply imported but not cleaved? Are both molecular species catalytically active?*

<u>Answer</u>: We refer the reviewer to our response above. Fluorescence labelling in Fig. 3 offers a more reliable explanation about the localization of TS, and engineered P450 module in plant cells. The new blot with truncated TS carried out during this revision showed a single band (Fig. R5) supporting that there could be slow processing of TS preprotein in our tobacco leaf expression conditions. Though slow processing is a possibility to explain the three bands, fluorescence labelling gives more compelling evidence that most of the protein is localized in

plastids, which indirectly points to a scenario where most protein is imported, but there might be slow processing of the signal sequences. As mentioned above, we couldn't do further experiments to determine, with certainty, the versions of TS proteins in the cells, though in *E. coli*, there is evidence that the full length and two other truncated versions of TS are soluble and catalytically comparable to some extent. Overally, the engineering of TS, T5H, and CPR in chloroplasts significantly improved the production of oxygenated taxanes showing that these heterologous enzymes are active.

As stated above, this is very interesting, and might add another engineering target to improve activity of chloroplast expressed proteins via targeting post-transcriptional processing machinery. These are some of the possibilities we aim to pursue in improving yields and advancing engineering of the challenging taxol pathway in plants. Thanks again for the valuable comments.

Comment:*Figure 4 depicts the taxane accumulation profiles for the 3 proteins targeted to the cytosol and plastid, and there is unexpected results here as well. When TS is targeted to the plastid, yet the P450 and reductase are directed to the cytosol, taxane accumulation is ~ 4 µg/g FW and oxidized taxane is about 1. When all three enzymes are directed to the cytosol, taxane level is boosted a bit as is the oxidized form. Then, when all three are targeted to the plastid, both the taxane and oxidized forms double in amounts. Why is this the case? Why doesn't targeting of the TS to the plastid itself able to yield taxane levels when all three proteins are targeted to the plastid?*

Answer: Mismatches in the recovered product yield between individually expressed TS and when the additional oxygenation module has been introduced are precedented in bacterial systems [Ajikumar, *et al*. (2010) Isoprenoid pathway optimization for Taxol precursor overproduction in Escherichia coli, Science 330, 70-74]. In that groundbreaking work, a carefully optimized *E. coli* strain accumulated nearly 1000 mg/L taxadiene but could only achieve a combined 50mg/L of the two major oxidized taxanes, without much residual taxadiene (~ 98% conversion rate) following the introduction of T5aH and CPR. In bacterial systems, this is attributed to disruption of the balance between upstream and downstream pathways due to introduction of the P450/CPR module. Further investigations revealed that the effect was not at transcript level, but at protein level where introduction of T5aH/CPR drastically reduced levels of TS, GGPPS and even upstream MEP pathway genes [Biggs, et al.(2016) Overcoming heterologous protein interdependency to optimize P450-mediated Taxol precursor synthesis in Escherichia coli. Proc. Natl. Acad. Sci. 113, 3209–3214.]. In plant systems, taxadiene-5a-ol has not yet been detected, but there were also some imbalances reported in the yield of OCT after introduction of T5aH in *Nicotiana Sylvestris* trichome cells compared to individually expressed TS that accumulated higher amounts (20ug/g) of taxadiene [Rontein, *et al*. (2008) CYP725A4 from yew catalyzes complex structural rearrangement of taxa-4(5),11(12)-diene into the cyclic ether 5(12)-oxa-3(11)-cyclotaxane. *J. Biol. Chem.***283**, 6067–6075]. We believe the imbalances in the yields of our constructs here can be explained, in part, by the differences in the stability of the P450 modules in different constructs that could have impacted upstream modules including TS activity. In addition, the introduction of TS modulated upstream transcript levels including ispD, ispE and ispF (see figure 5a) giving further weight to the interdependency of the modules, explaining the discrepancy in different constructs. Though not further investigated, we speculate that the increase in taxadiene yield when TS, T5aH and CPR were all targeted to the chloroplast compared to when TS was individually expressed in the chloroplast is explained by disruption of the upstream pathway balance depending on the stability of the P450/CPR module.

Comment: *The final piece of evidence provided is when DXS, GGPS, TS, P450 and Reductase are all targeted to the plastid and the taxane profiles measured. Quite surprising here, the taxane level is reduced to about one-quarter of that when just targeting the TS protein to the plastid. But then, one might imagine that the level of oxidized products were correspondingly increased. But that too isn’t quite the case. The level is marginally increased above that when the TS, P450 and Reductase are all targeted to the plastid (Fig. 4). So, if the results are as reproducible as the authors suggest in the rebuttal letter, then more than two-thirds of the diterpene hydrocarbon remains unaccounted for in the experiment depicted in Fig. 6.*

Answer: As noted by the reviewer, targeting all heterologous genes (DXS, GGPPS, TS, T5aH and CPR) in our final chloroplast compartmentalized construct yielded 15.2 ug/g FW taxadiene (reduced by about one-quarter compared to DXS-GGPPS-TS in Fig 5b). Indeed, the expected ideal result in the final chloroplast construct was to achieve maximal conversion of taxadiene to oxidized taxanes, thus the yield of oxidized taxanes was expected to correspondingly increase in Fig. 6. The two transformations are a bit different; we used two separate strains, GV3101-pEAQ-DXS-GGPPS and GV3101-pEAQ-TS-tp(TS)/trT5H/trCPR to complete the pathway engineering, using a final OD adjusted to 0.5 for the mixture, while the GV3101-pEAQ-DXS-GGPPS-TS was a single strain (OD = 0.5 also) for taxadiene production. Thus, the single strain was more efficiently expressed, and had a higher OD compared to the co-transformed strains which have lower protein levels. Increasing cell concentration of the double strains to 1.0 was not very helpful as it lead to drooping and wilting of leaves.

Comment: *When I pointed out previously the prior research articles cited didn’t necessarily focus on diterpenes, I had hoped the authors would appreciate that this reviewer examined those papers closely as well. Perhaps I should have been more explicit. What is uncanny in the current manuscript is how the work in this manuscript follows upon that described by Wu et al. Including how figures are constructed and the strategies used to unravel the contribution of the MVA and MEP pathway to terpene metabolism installed in the plastid versus cytosol. True, that work focused on another class of terpenes, but the approaches and strategies employed in the current work are almost identical, yet portrayed as if originating with the current authors. What is equally important to note, the Wu et al. work occurred long ago and there have been many additional efforts to install terpene synthases and decorating enzymes into the chloroplast to effect complex biosynthesis. Maybe the authors are unaware of these efforts. But it is not justified to portray the current work without due reference to the conceptual frontier provided by the work of others. The current work is good and important, but it is neither ground breaking nor precedent setting. It is the logical progression of solid scientific progress.*

Answer: We greatly appreciate the reviewers’ time and attention to detail and we appreciate that these concerns are raised at this point in time. Thanks for raising and clarifying this point, we sincerely apologize for missing it in our last revision. Wu et al work [Wu, S. *et al*. (2006) Redirection of cytosolic or chloroplastic isoprenoid precursors elevates terpene production in plants. Nature Biotechnology. 24, 1441–1447] is one of the pioneering and most important report on compartmentalized engineering of terpenoids and indeed, we carefully read the paper, but there are differences in the strategies we used to engineer the complex taxol pathway. As is very clear in our narrative in this manuscript, this contribution is not claiming to originate the concept of compartmentalized metabolic engineering in plant chassis. We agree that great precedent strategies had been reported for the conceptual framework, and have been demonstrated in various class of terpenoids. We cited and acknowledged the pioneering

work of Wu et al in compartmentalized metabolic engineering and also mentioned this strategy is key to overcoming the challenges of native compartmentalisation in plant cells. Given the complexity of the taxol pathway, the decades-long challenges in engineering even the first two committed steps, and the involvement of nearly nine P450s in the pathway, we leveraged compartmentalized engineering to successfully construct the early pathway in *Nicotiana benthamiana*.

The novelty of this contribution is in advancing taxane engineering in a plant system, presenting an alternative system to microbial systems that have demonstrated several drawbacks on expression of plant-based P450s in catalytically active forms. There is no deliberate attempt to downplay precedent work that laid the conceptual framework of the strategies leveraged here. That said, in addition to the different class of terpenes, there are differences between the work of Wu. et al and this contribution, including enhancement of precursor pathways that we believe are of interest in not only taxane pathway engineering, but metabolic engineering in general. That precedent work utilized plastidic or cytosolic targeting to gain access to higher precursor environments and to evade endogenous organelle-specific pathway regulatory mechanisms. In our case, the taxol biosynthetic pathway is catalyzed by enzymes that are natively distributed in different compartments, making it nearly impossible to engineer the pathway in plants without targeting to a particular compartment.
As mentioned by the reviewer, since the Wu et al work, many efforts, including very recent work like Sadre et al. [Sadre et al., Cytosolic lipid droplets as engineered organelles for production and accumulation of terpenoid biomaterials in leaves. ***Nature Communications*** (2019)] have been published describing targeting different terpenes into plant compartments, highlighting the versatility and potential of the strategy in utilization of plants as chassis. We are very much aware of improvements in targeting genes into the chloroplasts, but again, it has been more than a decade since the first attempt to construct the taxol pathway in a plant system has been published, and the lack of huge leaps in both improvement of yields and advancement of the pathway is proof of the myriad of challenges in engineering this particular pathway. These are definitely areas worth pursuing to improve engineering of the taxane pathway in plants. As for the presentation of Figures 4 and 6, we considered a layout that makes it easier and simpler for readers to follow the logic and quickly visualize the engineering efforts reported here.

Comment:*Lastly, the rebuttal letter and the revision leads to more questions than answers. For instance, the unknown "putative" triterpene that with mass fragments of 208 and 213. I'm not able to follow this logic. Such ions will be evident in sesqui-, di-, tri- and tetra-terpenes. This is just an example of the confusion created by the rebuttal letter - there are many others.*

Answer: We apologise for this imprecise description of the unknown peak; yes, those ions will be evident in a broad class of terpenes, not just triterpenes. Based on the fact that the compound accumulated in tobacco leaves individually expressing truncated HMGR but with no TS, we considered the compound to be a native tobacco terpene with no relationship to the engineered taxane pathway, thus not very critical for optimization and purification for NMR analyses. Nonetheless, during the course of this revision, we attempted isolating enough amounts of the metabolite from tHMGR transient tobacco for NMR identification. Though the compound seemed to be pure in GC analysis (Figure R7), only less than 1 mg was isolated and the chemical structure could not be precisely determined by NMR (Figure R8). We have revised the description of the compound in the manuscript to now read: "We could not purify enough amounts of the compound for structural analysis by NMR, but based on the mass spectra ions (m/z of 218 and 203), we speculated it to be a terpene."

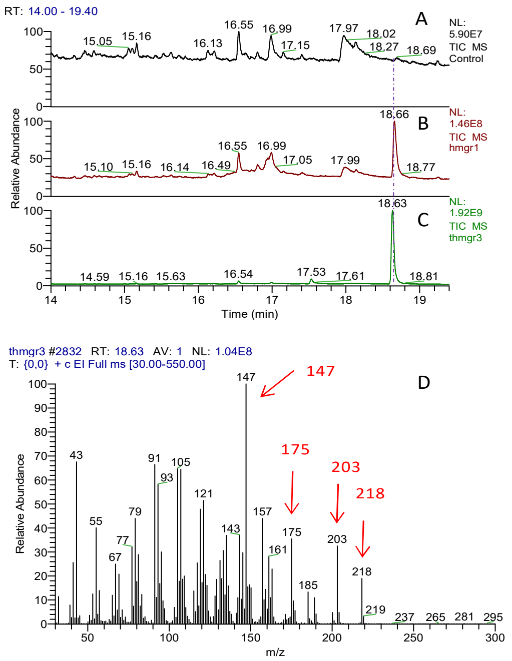

Figure S30. GC-MS analysis of metabolites in transient HMGR and tHMGR expression and in wild-type tobacco leaves. (A) TIC of extracts from wild type tobacco; (B) TIC of extracts from HMGR expressing tobacco; (C) TIC of extracts from tHMGR expressing tobacco. A trace amount of the unknown peak that eluted together with taxadiene could be detected in wild-type plants and the yield improved with the expression of HMGR. Overexpressing tHMGR drastically improved the peak; (D) Mass spectrum of the unknown compound.

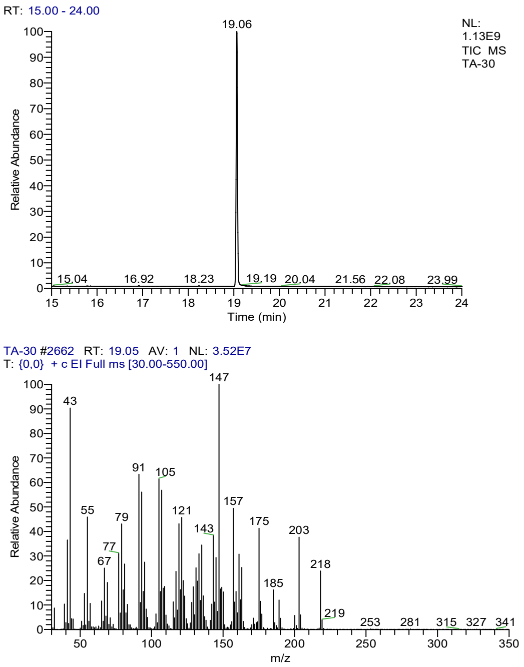

Figure R6. GC-MS analysis of metabolism from tobacco transient with tHMGR. Top panel showed the TIC of purified metabolism; Down panel showed the mass spectrum of purified metabolism.

Figure R7. Comparison of TIC and mass spectra of unknown compound (tR=18.63 min) produced by tHMGR transiently expressed *N. benthamiana* with the purified compound from transient plant tissue. The retention time of the compound was delayed by 0.4 min due to column regeneration.

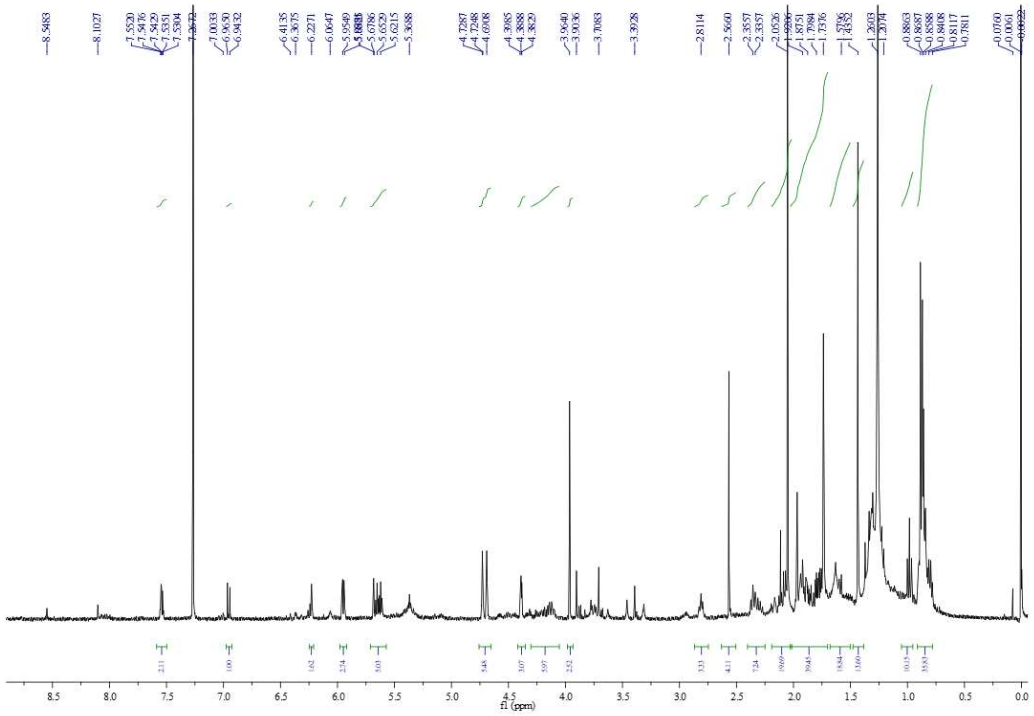

Figure R8 ^{1}H-NMR spectrum of the unknown compound.

Reviewer #4 (Remarks to the Author):

Comment: *The present work set up a metabolic pathway in the plant Nicotiana benthamiana for producing the precursors of Taxol. The authors demonstrated that the plant platform produced not only taxadiene but also taxadiene-5α-ol and the latter is the first report to be produced by the plant chasis. They also manifested that these precursors were mainly produced by the MEP pathway. The manuscript is meaningful for developing production platform in plants. The authors have basically answered the questions involved in the comment of the reviewer #1. And the manuscript has been substantially revised following the reviewers' suggestions, including supplementing some new data.*

Answer: We thank the reviewer for positive evaluation of our work.

Comment: *In the past decade, some outstanding achievements have been made by other research groups. For example, as mentioned by the authors, Ajikumar, et al. achieved ~1 g taxadiene/L and ~60 mg taxadiene-5α-ol/L, respectively, in an engineered E. coli strain [Ajikumar, et al. (2010) Isoprenoid pathway optimization for Taxol precursor overproduction in Escherichia coli, Science 330, 70-74]. This group also achieved ~570 mg oxygenated taxanes/L in E. coli [Biggs, et al.(2016) Overcoming heterologous protein interdependency to optimize P450-mediated Taxol precursor synthesis in Escherichia coli. Proc. Natl. Acad. Sci. 113, 3209–3214.].These represent the highest yields of the two taxanes produced by the metabolic engineering method. However, the two precursors are far from Taxol and even far from the two important intermediates 10-deacetylbaccatin III and baccatin III. Therefore, if the work to engineer the Taxol pathway into a heterologous host advances beyond the two precursors taxadiene and taxadiene-5α-ol, it will be more compelling. Of course it is an arduous job.*
I suggest the authors to reorganize the introduction to address these achievements in the yields, and in the discussion section, to compare the present yields with those previously reports.

Answer: The work by Ajikumar et al., (2010) and the more recent Biggs et al., (2016) represent the highest reported yields of taxadiene and oxygenated taxanes. Though these great contributions were described in our previous version of the manuscript, we have revised the manuscript to highlight these milestone achievements in the introduction and the discussion sections. It must be noted, however, that it is hard to directly compare the yields from these microbial systems to the ones reported here in a plant system with no added carbon feedstocks.

Comment: *Other comments can be found in the annotations of the manuscript.*

Answer: Thanks, the issues have been modified in the revised manuscript (L26, L37, L38, L95, L119, etc).

Reviewers' comments:

Reviewer #2 (Remarks to the Author):

The authors have addressed the concern and has now included 1H-NMR of taxadiene-5α-ol which gave three impurity signals at δ7.71 ppm, 7.54 ppm and 4.31 ppm (Figure S21). The impurity were expected and the authors should state that in the manuscript that impurity was expected but the the products spectra still matched to the product.

Reviewer #3 (Remarks to the Author):

The authors have again revised the manuscript to address the reviewers' comments. And I must say they have gone a long way in addressing many of the comments. But this reviewer has steadfastly questioned the overarching significance/importance/impact of the work and is yet convinced of such. The issue boils down to the following question, "Does the engineering of a terpene synthase and a complementary hydroxylase, a cytochrome P450 enzyme, into the plastid compartment and the observation of a small amount of a diterpene alcohol constitute a technical and conceptual breakthrough?" Maybe, if it were presented in a more wholistic context. I will once again present a perspective that isn't fully captured in the current manuscript, a conceptual one, that if captured might give me greater confidence in the significance of the work.

Are there nuclear encoded P450s that are natively targeted to the chloroplast? Yes, these include carotenoid hydroxylases and others associated with unique biochemical transformations of fatty acids. However, there are outstanding questions concerning these enzymes. Are they inserted into the plastid compartment? Do they utilize a typical N-terminal, plastid targeting signal sequence? In some cases the answer appears to be yes. In others, the P450s appear to be targeted to the outer thylakoid membrane. Why is this important? Because it speaks to the functional assembly of the P450 with a heme moiety when the pre-protein is imported into the stroma compartment and folded into a catalytically active enzyme. The bottom line here is that all the necessary machinery for import and assembly of catalytically active P450s exists natively in chloroplasts and it makes perfect sense that heterologously expressed P450s targeted to the stroma would be catalytically functional.

A second and equally important component for any P450 mediated reaction is its reducing partner. P450s can utilize a variety of electron donating partners, but these are often paired. P450s associated with the ER membrane often utilize the cytochrome P450 reductase partners that don't exhibit much discrimination for the P450 they reduce. And, we know that the taxadiene-5-hydroxylase does utilize a typical reductase associated with the cytoplasmic compartment. Hence, it isn't surprising to target both the P450 and the reductase to the plastid compartment in order to assure the P450 has all the necessary machinery to function. It also is key that the energy for the reductase is provided by NADPH, an abundant and plentiful cofactor generated by the photosynthetic cycle occurring in the chloroplast.

The work at another level consists essentially of building a scaffold within the plastid compartment, then decorating it. Are there other examples of such engineering? Yes, hydroxylations and methylations are two such examples of decorating plastid produced scaffolds, but these precedents don't seem to be recognized in the current manuscript.

If the selling point of the current work is building alternative production platforms for complex molecules like taxol, shouldn't the authors also be capturing some of the innovative chemical engineering efforts with Taxus plant materials as well? In particular, the work from the Loake laboratory almost 10 years ago described the isolation of cambial stem cells from Taxus that actually produce the complete taxol molecule in yields that dwarf the N. benthamiana platform by orders of magnitude.

I’ve read and re-read this manuscript many times now and know my criticisms above are harsh. I’m not worried about the chemical identification of products like another reviewer. The authors can clearly purify molecules that accumulate in miniscule amounts and provide NMR characterization. I am, however, more focused on truly how innovative is this work and how deep is the investigation. Another example of the later – did they ever try constructs without the reductase fused to the P450? One might not expect this to work, but then installing a conventional P450 into the plastid compartment and evaluating if other reducing partners might provide the necessary reducing equivalents might a perspective on the evolutionary relationships between P450s and their reducing partners. I recognize this is derivative from the main point of the current manuscript, but this is an example of the depth of appreciation I would expect for a paper to appear in a Nature journal.

My last point relates to what I suspect is a minor point in the current manuscript. The authors identify a metabolite that overlaps with taxadiene that seems to originate natively from the N. benthamiana. Yep, this too isn’t surprising. The defense responses of tobacco are known to be induced by agrobacterium and some of the phytoalexins are terpenoid derived.

Reviewers' comments:

Reviewer #2 (Remarks to the Author):

The authors have addressed the concern and has now included 1H-NMR of taxadiene-5α-ol which gave three impurity signals at δ7.71 ppm, 7.54 ppm and 4.31 ppm (Figure S21). The impurity were expected and the authors should state that in the manuscript that impurity was expected but the products spectra still matched to the product.

Reviewer #3 (Remarks to the Author):

The authors have again revised the manuscript to address the reviewers' comments. And I must say they have gone a long way in addressing many of the comments. But this reviewer has steadfastly questioned the overarching significance/importance/impact of the work and is yet convinced of such. The issue boils down to the following question, "Does the engineering of a terpene synthase and a complementary hydroxylase, a cytochrome P450 enzyme, into the plastid compartment and the observation of a small amount of a diterpene alcohol constitute a technical and conceptual breakthrough?" Maybe, if it were presented in a more wholistic context. I will once again present a perspective that isn't fully captured in the current manuscript, a conceptual one, that if captured might give me greater confidence in the significance of the work.

Are there nuclear encoded P450s that are natively targeted to the chloroplast? Yes, these include carotenoid hydroxylases and others associated with unique biochemical transformations of fatty acids. However, there are outstanding questions concerning these enzymes. Are they inserted into the plastid compartment? Do they utilize a typical N-terminal, plastid targeting signal sequence? In some cases the answer appears to be yes. In others, the P450s appear to be targeted to the outer thylakoid membrane. Why is this important? Because it speaks to the functional assembly of the P450 with a heme moiety when the pre-protein is imported into the stroma compartment and folded into a catalytically active enzyme. The bottom line here is that all the necessary machinery for import and assembly of catalytically active P450s exists natively in chloroplasts and it makes perfect sense that heterologously expressed P450s targeted to the stroma would be catalytically functional.

A second and equally important component for any P450 mediated reaction is its reducing partner. P450s can utilize a variety of electron donating partners, but these are often paired. P450s associated with the ER membrane often utilize the cytochrome P450 reductase partners that don't exhibit much discrimination for the P450 they reduce. And, we know that the taxadiene-5-hydroxylase does utilize a typical reductase associated with the cytoplasmic compartment. Hence, it isn't surprising to target both the P450 and the reductase to the plastid compartment in order to assure the P450 has all the necessary machinery to function. It also is key that the energy for the reductase is provided by NADPH, an abundant and plentiful cofactor generated by the photosynthetic cycle occurring in the chloroplast.

The work at another level consists essentially of building a scaffold within the plastid compartment, then decorating it. Are there other examples of such engineering? Yes, hydroxylations and methylations are two such examples of decorating plastid produced scaffolds, but these precedents don't seem to be recognized in the current manuscript.

If the selling point of the current work is building alternative production platforms for complex molecules like taxol, shouldn't the authors also be capturing some of the innovative chemical engineering efforts with Taxus plant materials as well? In particular, the work from the Loake laboratory almost 10 years ago described the isolation of cambial stem cells from Taxus that actually produce the complete taxol molecule in yields that dwarf the N. benthamiana platform by orders of magnitude.
I've read and re-read this manuscript many times now and know my criticisms above are harsh. I'm not worried about the chemical identification of products like another reviewer. The authors can clearly purify molecules that accumulate in miniscule amounts and provide NMR characterization. I am, however, more focused on truly how innovative is this work and how deep is the investigation. Another example of the later – did they ever try constructs without the reductase fused to the P450? One might not expect this to work, but then installing a conventional P450 into the plastid compartment and evaluating if other reducing partners might provide the necessary reducing equivalents might a perspective on the evolutionary relationships between P450s and their reducing partners. I recognize this is derivative from the main point of the current manuscript, but this is an example of the depth of appreciation I would expect for a paper to appear in a Nature journal.

My last point relates to what I suspect is a minor point in the current manuscript. The authors identify a metabolite that overlaps with taxadiene that seems to originate natively from the N. benthamiana. Yep, this too isn't surprising. The defense responses of tobacco are known to be induced by agrobacterium and some of the phytoalexins are terpenoid derived.

Point-by-point response to reviewers' comments:

Reviewer #2 (Remarks to the Author):

Comment: *The authors have addressed the concern and has now included 1H-NMR of taxadiene-5α-ol which gave three impurity signals at δ7.71 ppm, 7.54 ppm and 4.31 ppm (Figure S21). The impurity were expected and the authors should state that in the manuscript that impurity was expected but the the products spectra still matched to the product.*

Answer: Thanks for this advice. We have added the NMR description and details on the impurity signals in the results section (Line 239-243) and in the supplementary experimental section.

Reviewer #3 (Remarks to the Author):

***__Comment__*:** *The authors have again revised the manuscript to address the reviewers' comments. And I must say they have gone a long way in addressing many of the comments. But this reviewer has steadfastly questioned the overarching significance/importance/impact of the work and is yet convinced of such. The issue boils down to the following question, "Does the engineering of a terpene synthase and a complementary hydroxylase, a cytochrome P450 enzyme, into the plastid compartment and the observation of a small amount of a diterpene alcohol constitute a technical and conceptual breakthrough?" Maybe, if it were presented in a more wholistic context.*

__Answer__: We once again appreciate the valuable time of the Reviewer on improving our manuscript, and we welcome these positive criticisms and well-thought suggestions. The major thrust of this contribution is construction of an alternative synthetic production platform of a high value natural product like paclitaxel, by engineering the pathway in chloroplasts. We believe this work is more than just engineering of a terpene synthase and a cytochrome P450 enzyme into the plastid compartment. There are still a lot of outstanding questions surrounding taxol biosynthesis in plant cells, as evident from the fact that despite decades of research, not much is known about the regulation of the pathway, and that even the complete pathway enzymes are yet to be discovered. Our engineering efforts were designed to overcome a classical hurdle in the native taxol pathway where pathway enzymes are natively localized in different compartments and the internal transport of metabolites (and possibly proteins) in native *Taxus* cells is still unclear. Despite the development of *Eschericha col*i and yeast systems in the previous years, plant-based synthetic platforms are more promising for improved P450 chemistry as they might open previously inaccessible possibilities in the complex pathway. So, this work sets the stage for expansion of the work in plant systems as the involvement of nearly nine plant-originated P450s in taxol synthesis present an unsurmountable barrier in microbial chassis. In addition to presentation of tools for developing plant-based systems, the study also explores transient protein expression landscape, assesses contribution of precursor pathways, improves precursor supply and explores gene expression of key genes in the context of heterologous taxol biosynthesis. Compared to microbial-based systems that are already optimally developed and that use added sugars and cofactors, and taking into consideration that taxadiene still accumulated even after detection of oxygenated taxanes in our tobacco system, we believe the strategies are very promising, and the yields achieved here are remarkable.

I will once again present a perspective that isn't fully captured in the current manuscript, a conceptual one, that if captured might give me greater confidence in the significance of the work. Are there nuclear encoded P450s that are natively targeted to the chloroplast? Yes, these include carotenoid hydroxylases and others associated with unique biochemical transformations of fatty acids. However, there are outstanding questions concerning these enzymes. Are they inserted into the plastid compartment? Do they utilize a typical N-terminal, plastid targeting signal sequence? In some cases the answer appears to be yes. In others, the P450s appear to be targeted to the outer thylakoid membrane. Why is this important? Because it speaks to the functional assembly of the P450 with a heme moiety when the pre-protein is imported into the stroma compartment and folded into a catalytically active enzyme. The bottom line here is that all the necessary machinery for import and assembly of catalytically

active P450s exists natively in chloroplasts and it makes perfect sense that heterologously expressed P450s targeted to the stroma would be catalytically functional.

Answer: The reviewer raises a very interesting conceptual perspective that we also considered during the design of this study. Yes, there are P450s that are natively targeted to the chloroplast, with prominent examples as carotenoid hydroxylases, allene oxide synthase and hydroperoxide lyases (Froehlich et al., 2001; Tian et al., 2001). Engineering P450 chemistry in plastids, thus, should learn from these native P450s. As mentioned by the reviewer, chloroplast import mechanisms and intraorganellar routing and processing of nuclear encoded proteins is highly complex, and there are still many questions surrounding these P450s. Despite this structural intricacy, the mere existence of these P450s natively targeted to the chloroplast makes the chloroplast an attractive site for targeting heterologous P450s. Actually, our engineering efforts in this study were inspired by this fact, and the realization that the machinery for import and assembly of catalytically active P450s exist in plastids. We agree it would be far more informative to explore the posttranslational processing and intricate intra-chloroplastic targeting of our engineered taxadiene-5a-ol-CPR proteins, as this will provide more details compared to fluorescence microscopy localization evidence. Moreover, tethering the P450 to the outer thylakoid membrane can ensure that the P450 acquire electrons directly from photosystem I, rather than from CPR, as has been demonstrated from studies in the Jensen group (de Jesus et al., 2017). However, this specific targeting has its own challenges regarding the protein scaffold itself and optimizations needed on complex pathways like the taxol pathway. We realized this complexity during the design of this study, and decided to leverage the native targeting signal peptide of taxadiene synthase (TS) to engineer the T5H-CPR fusion protein. Given the yet unclear processing events of TS itself in the chloroplast, we deliberately used the transit peptide from TS to engineer the P450 and CPR fusion to ensure similar targeting, post-translational processing and intra-chloroplastic routing of the pathway proteins to achieve maximal optimization. Though not yet tested, we acknowledge that more precise thylakoid membrane anchoring of this scaffold could potentially improve pathway optimizations by by-passing the cytochrome P450 reductase, but the use of the native TS signal peptide was considered more optimal in this design. However, more in depth investigations of TS processing (so as to engineer P450 chemistry to be closer to native TS in the plastid) and the use of the pH-dependent twin-arginine translocation (Tat) complex (for more elaborate thylakoid membrane anchoring of the protein scaffold) are definitely experiments we are considering for the future. We have added discussion of this interesting perspective in our revised manuscript, together with relevant citations and believe this is very valuable to the readers (L413-416, L421-427).

Comment: *The work at another level consists essentially of building a scaffold within the plastid compartment, then decorating it. Are there other examples of such engineering? Yes, hydroxylations and methylations are two such examples of decorating plastid produced scaffolds, but these precedents don't seem to be recognized in the current manuscript.*

Answer: Thanks for this comment. We have acknowledged and cited key hydroxylation examples in the manuscript, such as dhurrin pathway engineering in chloroplast by de Jesus (de Jesus et al., 2017), and we have now added a description of this precedent development and cited this key publication in the revised manuscript (Line 413-416). But we apologize for missing methylation decoration examples of plastid produced scaffolds.

If the selling point of the current work is building alternative production platforms for complex molecules like taxol, shouldn't the authors also be capturing some of the innovative chemical

engineering efforts with Taxus plant materials as well? In particular, the work from the Loake laboratory almost 10 years ago described the isolation of cambial stem cells from Taxus that actually produce the complete taxol molecule in yields that dwarf the N. benthamiana platform by orders of magnitude.

Answer: We tried to capture as much chemical engineering efforts of taxol and other terpenoids as much as possible in our previous version of the manuscript and we apologize for missing the key work from the Laoke group (Lee et al., 2010). We have now added this key citation (Line 62). Indeed, in last decades, many researchers set effort on the plant cell culture for taxol production (Lee et al., 2010; Wang et al., 2018; Son et al., 2000). Although the final taxol molecular can be generated in this way, this platform still encountered several significant limitations including: a relatively slow growth rate, instability of product yield, generation of low concentration. Most importantly, this synthetic biology platform with taxus stem-cell is restricted by the rarely understandings of the genetic background of taxus plant cells and genetic manipulation technology. We then turned to transfer the taxol pathway into well-established hosts and threw our eager to explore the possibility of taxol production.

Comment: *I've read and re-read this manuscript many times now and know my criticisms above are harsh. I'm not worried about the chemical identification of products like another reviewer. The authors can clearly purify molecules that accumulate in miniscule amounts and provide NMR characterization. I am, however, more focused on truly how innovative is this work and how deep is the investigation. Another example of the later – did they ever try constructs without the reductase fused to the P450? One might not expect this to work, but then installing a conventional P450 into the plastid compartment and evaluating if other reducing partners might provide the necessary reducing equivalents might a perspective on the evolutionary relationships between P450s and their reducing partners. I recognize this is derivative from the main point of the current manuscript, but this is an example of the depth of appreciation I would expect for a paper to appear in a Nature journal.*

Answer: We thank the reviewer for this suggestion. We carefully considered the reductase partner for taxadiene-5α-hydroxylase, and initially excluded the reductase partner in the hope of leveraging the native tobacco reductases, but unfortunately no oxidised taxane product was detected. Rontein et al., (2008) attempted a similar engineering strategy utilizing taxadiene synthase and T5H without a cytochrome P450 reductase partner (though the two enzymes were not targeted to the same compartment) and could only detect 5(12)-oxa-3(11)-cyclotaxane, and not the expected taxadiene-5α-ol in *Nicotiana Sylvestris*. While the detected activity of a P450 with its reductase partner was not surprising in our system, the major challenge is in optimization and maintenance of balance in the system. Together with the other chloroplast advantages mentioned by the reviewer and in our response above, we believe the chloroplast, with its abundance of NADPH, is ideal for engineering complicated pathways that include a lot of P450s like the taxol pathway, as this seems to be the main limitation that have stalled progress for decades in both microbial and plant systems.

Comment: *My last point relates to what I suspect is a minor point in the current manuscript. The authors identify a metabolite that overlaps with taxadiene that seems to originate natively from the N. benthamiana. Yep, this too isn't surprising. The defense responses of tobacco are known to be induced by agrobacterium and some of the phytoalexins are terpenoid derived.*

Answer: We thank the reviewer for this suggestion. We have added a few lines to supplement our description of the metabolite (L297-301).

References：

1. Tian, L. & DellaPenna, D. (2001) Characterization of a second carotenoid b-hydroxylase gene from Arabidopsis and its relationship to the LUT1 locus. *Plant Mol. Biol.* 47, 379–388.

2. Froehlich, J.E., Itoh, A., & Howe, G.A. (2001) Tomato allene oxide synthase and fatty acid hydroperoxide lyase, two cytochrome P450s involved in oxylipin metabolism, are targeted to different membranes of chloroplast envelope. *Plant Physiol.* 125, 306-317.

3. de Jesus, M.P.R.H., Nielson, A.Z., Mellor, S.B., Matthes, A., Burow, M., Robinson, C., & Jensen, P.E. (2017) Tat proteins as novel thylakoid membrane anchors organize a biosynthetic pathway in chloroplasts and increase product yield 5-fold. *Metab. Eng.* 44, 108-116.

4. Rontein, D. et al. CYP725A4 from yew catalyzes complex structural rearrangement of taxa-4(5),11(12)-diene into the cyclic ether 5(12)-oxa-3(11)-cyclotaxane. J. Biol. Chem. 283, 6067–6075 (2008).

5. Lee, E.-K., Jin, Y.-W., Park, J.H., Yoo, Y.M., Hong, S.M., Amir, R., Yan, Z., Kwon, E., Elfick, A., Tomlinson, S., Halbritter, F., Waibel, T., Yun, B.-Y., Loake, G.J. Cultured cambial meristematic cells as a source of plant natural products. Nat. Biotechnol. 2010, 28, 1213-1217.

6. Wang, S., Wang, H., Li, T., Li, C., Zhou, Y., Zhong, X. The selection and stability analysis of stable and high Taxol-producing cell lines from Taxus cuspidate. J. Forestry Res. 2018, 29, 65-71.

7. Son, S.H., Choi, S.M., Lee, Y.H., Choi, K.B., Yun, S.R., Kim, J.K., Park, H.J., Kwon, O.W., Noh, E.W., Seon, J.H., Park, Y.G. Large-scale growth and taxane production in cell cultures of Taxus cuspidate (Japanese yew) using a novel bioreactor. Plant Cell Rep. 2000, 19, 628-633.

REVIEWERS' COMMENTS:

Reviewer #3 (Remarks to the Author):

I appreciate the authors responses to my previous concerns. What I've argued in my previous reviews is that the work doesn't represent either the technical or conceptual breakthrough that I expect in a Nature article. The work presented is great. I've just not been convinced that it is transformational and will lead a community of scientists to follow this path.

The authors use the term remarkable in the manuscript and one should rightly question such claims. Is µg quantities of a compound per gm fresh weight remarkable? There is some old technoeconomic analyses that suggest that plant production platforms must be at the 4 to 5% level in order to be economically viable. That translates to 40 to 50 mg per gm fresh weight, which is more than a 1,000 fold greater than that reported here. So, is the authors' claim of "remarkable" correct from a technical perspective?

I suspect that pharmaceutical companies won't find such platforms attractive without a cost-benefit of at least 10-fold better than traditional fermentation platforms. Think about this, what would compel you to stop using an infrastructure that cost you 10s if not 100s of millions of dollars to build and to switch to another production platform? Wouldn't you want to recoup any investment in building a new platform within ~10 years, and be able to see that this new platform would offer 2nd generation to an infinite number of new product opportunities?

If the work doesn't pass the technical threshold of transformational, is it conceptually transformation? Perhaps. The notion of installing the biosynthetic machinery into a compartment that can channel intermediates to building complex molecules is the challenge here. Putting a terpene biosynthetic pathway into the plastid compartment is not new, as noted by several of the cited references. But designing the intra-organellar architecture for this might be. And, is this what the authors are providing? The following statement is directly from the manuscript and it starts to get to this point.

"Despite the development of Eschericha coli and yeast systems in the previous years, plant-based synthetic platforms are more promising for improved P450 chemistry as they might open previously inaccessible possibilities in the complex pathway."

I don't understand this comment and don't appreciate how any advantages of expressing P450s in plants wouldn't be the same or better in E. coli and yeast. I could be missing something here, but could the authors provide some scientific evidence/rationale for such a statement and point to some experimental evidence in the current work which supports their thesis?

There are a few grammatical issues. Spelling of E. coli (as above) for instance. Isn't CFP appended to the carboxy terminus of the T5alphaH construct? Need to provide references for the medically relevant proteins produced in plants. An editorial review to remove superlatives would also much appreciated.

Reviewers' comments:

Reviewer #3 (Remarks to the Author):

I appreciate the authors responses to my previous concerns. What I've argued in my previous reviews is that the work doesn't represent either the technical or conceptual breakthrough that I expect in a Nature article. The work presented is great. I've just not been convinced that it is transformational and will lead a community of scientists to follow this path.

The authors use the term remarkable in the manuscript and one should rightly question such claims. Is µg quantities of a compound per gm fresh weight remarkable? There is some old technoeconomic analyses that suggest that plant production platforms must be at the 4 to 5% level in order to be economically viable. That translates to 40 to 50 mg per gm fresh weight, which is more than a 1,000 fold greater than that reported here. So, is the authors' claim of "remarkable" correct from a technical perspective?

I suspect that pharmaceutical companies won't find such platforms attractive without a cost-benefit of at least 10-fold better than traditional fermentation platforms. Think about this, what would compel you to stop using an infrastructure that cost you 10s if not 100s of millions of dollars to build and to switch to another production platform? Wouldn't you want to recoup any investment in building a new platform within ~10 years, and be able to see that this new platform would offer 2nd generation to an infinite number of new product opportunities?

If the work doesn't pass the technical threshold of transformational, is it conceptually transformation? Perhaps. The notion of installing the biosynthetic machinery into a compartment that can channel intermediates to building complex molecules is the challenge here. Putting a terpene biosynthetic pathway into the plastid compartment is not new, as noted by several of the cited references. But designing the intra-organellar architecture for this might be. And, is this what the authors are providing? The following statement is directly from the manuscript and it starts to get to this point.

"Despite the development of Eschericha coli and yeast systems in the previous years, plant-based synthetic platforms are more promising for improved P450 chemistry as they might open previously inaccessible possibilities in the complex pathway."

I don't understand this comment and don't appreciate how any advantages of expressing P450s in plants wouldn't be the same or better in E. coli and yeast. I could be missing something here, but could the authors provide some scientific evidence/rationale for such a statement and point to some experimental evidence in the current work which supports their thesis?

There are a few grammatical issues. Spelling of E. coli (as above) for instance. Isn't CFP appended to the carboxy terminus of the T5alphaH construct? Need to provide references for the medically relevant proteins produced in plants. An editorial review to remove superlatives would also much appreciated.

Point-by-point response to reviewer's comments:

Reviewer #3 (Remarks to the Author):

***Comment**:* *The authors use the term remarkable in the manuscript and one should rightly question such claims. Is µg quantities of a compound per gm fresh weight remarkable? There is some old technoeconomic analyses that suggest that plant production platforms must be at the 4 to 5% level in order to be economically viable. That translates to 40 to 50 mg per gm fresh weight, which is more than a 1,000 fold greater than that reported here. So, is the authors' claim of "remarkable" correct from a technical perspective?*

I suspect that pharmaceutical companies won't find such platforms attractive without a cost-benefit of at least 10-fold better than traditional fermentation platforms. Think about this, what would compel you to stop using an infrastructure that cost you 10s if not 100s of millions of dollars to build and to switch to another production platform? Wouldn't you want to recoup any investment in building a new platform within ~10 years, and be able to see that this new platform would offer 2nd generation to an infinite number of new product opportunities?

Answer: We once again thank the Reviewer for their valuable time and these very insightful perspectives, comments and constructive criticisms that have really helped us to improve our manuscript.
We apologize for the use of superlatives and subjective terms in the manuscript. We have carefully revised the entire manuscript and deleted such words and phrases.
The yield in tobacco platform as reported in this work might not be enough for industrial application yet, but it is our hope that this platform, together with compartmentalized engineering strategy, sets the stage for further optimization to improve yields not only of taxol and its intermediates, but of other complex natural products as well. In the context of taxol, the cytochrome P450 expression challenge poses the biggest obstacle, necessitating exploration of alternative platforms to overcome the hurdle.
The point on industrial application and adoption of plant production systems is an interesting discussion. Low start-up cost is one of the attractive advantages of plant platforms over microbial-based platforms for synthetic production of plant natural products. This, combined with the fact that plant platforms require less carbon substrates and cofactor inputs is expected to attract their use as alternative production systems. However, as discussed in the manuscript, plant-based platforms still suffer from their own shortcomings, mainly around the complexity of engineering pathways and balancing these with normal plant growth and defense, channeling of metabolites to desired products, etc., but in general, once some of these challenges are addressed, the concept of harvesting CO_2 and energy from the sun must be attractive enough for industrial applications. We argue that the huge number of specialized cytochrome P450s in the taxol pathway presents non trivial hurdles for successful development of biosynthetic platforms as highlighted by studies with microbial-based platforms so far, hence the urgent need to develop alternative platforms that could be more compatible with plant P450 chemistry.

Comment: *Putting a terpene biosynthetic pathway into the plastid compartment is not new, as noted by several of the cited references. But designing the intra-organellar architecture for this might be. And, is this what the authors are providing? The following statement is directly from the manuscript and it starts to get to this point.*

“Despite the development of Eschericha coli and yeast systems in the previous years, plant-based synthetic platforms are more promising for improved P450 chemistry as they might open previously inaccessible possibilities in the complex pathway.”

I don’t understand this comment and don’t appreciate how any advantages of expressing P450s in plants wouldn’t be the same or better in E. coli and yeast. I could be missing something here, but could the authors provide some scientific evidence/rationale for such a statement and point to some experimental evidence in the current work which supports their thesis?

.

Answer: We have attempted to highlight the engineering strategies undertaken in this study in the manuscript, and forwarded arguments on the need for such strategies in the development of platforms to tackle complex natural products.

The statement referred to above, included in our last rebuttal letter, pertains to advantages of engineering plants to be alternative production systems for plant-originated genes and pathways. Indeed, microbial systems are well studied and have already been commercialized for the vast majority of natural products. As mentioned in our response above, the common bottleneck that emerged from attempts to engineer the taxol pathway in microbial hosts is functional expression of plant cytochrome P450s. As these P450s are of plant origin, plant cells are expected to be more compatible with their successful functional expression, as has been demonstrated by many other studies (Reviewed by Ikram et al., 2015). Plants seem to have inherent advantages over microbes in supporting plant cytochrome P450 functional expression since they support correct mRNA and protein processing as well as proper compartmentalization. Also, they already have many of the necessary metabolic precursors and cofactors, thus extensive engineering of host pathways can be avoided. Several plant P450s have been reported difficult to functionalize in microbial hosts. The current study did not directly compare functional expression landscapes of enzymes in plant and microbial platforms, thus, without direct experimental evidence in the current work, the statements to support inherent advantages of plant systems in P450 chemistry in the manuscript were inferred from literature. We have added a citation (Ikram et al., 2015) in the revised manuscript to support the statement and improve clarity (see Introduction - L93 and Discussion L382).

Comment: *There are a few grammatical issues. Spelling of E. coli (as above) for instance. Isn’t CFP appended to the carboxy terminus of the T5alphaH construct? Need to provide references for the medically relevant proteins produced in plants. An editorial review to remove superlatives would also much appreciated.*

Answer: We apologize for grammatical mistakes and typos. We have carefully revised the manuscript and cleared all grammatical issues.

Yes, CFP is appended to the carboxy terminus of T5αH, not the N-terminus. We have corrected this in text and figures.

Following Editorial review requirements to shorten the length of the manuscript, we have now removed the extended discussion on molecular pharming for production of medically relevant proteins, though these represent most of the success stories of plant synthetic production platforms to date.

References

1. Ikram, N.K.B.K., Zhan, X., Pan, X., King, B. C. & Simonsen, H.T. Stable heterologous expression of biologically active terpenoids in green plant cells. *Front. Plant. Sci*. **6**:129 (2015).
